# Supplementary figures and images for: Predicting knee osteoarthritis progression using neural network with longitudinal MRI radiomics, and biochemical biomarkers: A modeling study
Source: PLoS Med. 2025 Aug 21;22(8):e1004665. doi: 10.1371/journal.pmed.1004665 (PMC12370028; doi:10.1371/journal.pmed.1004665)

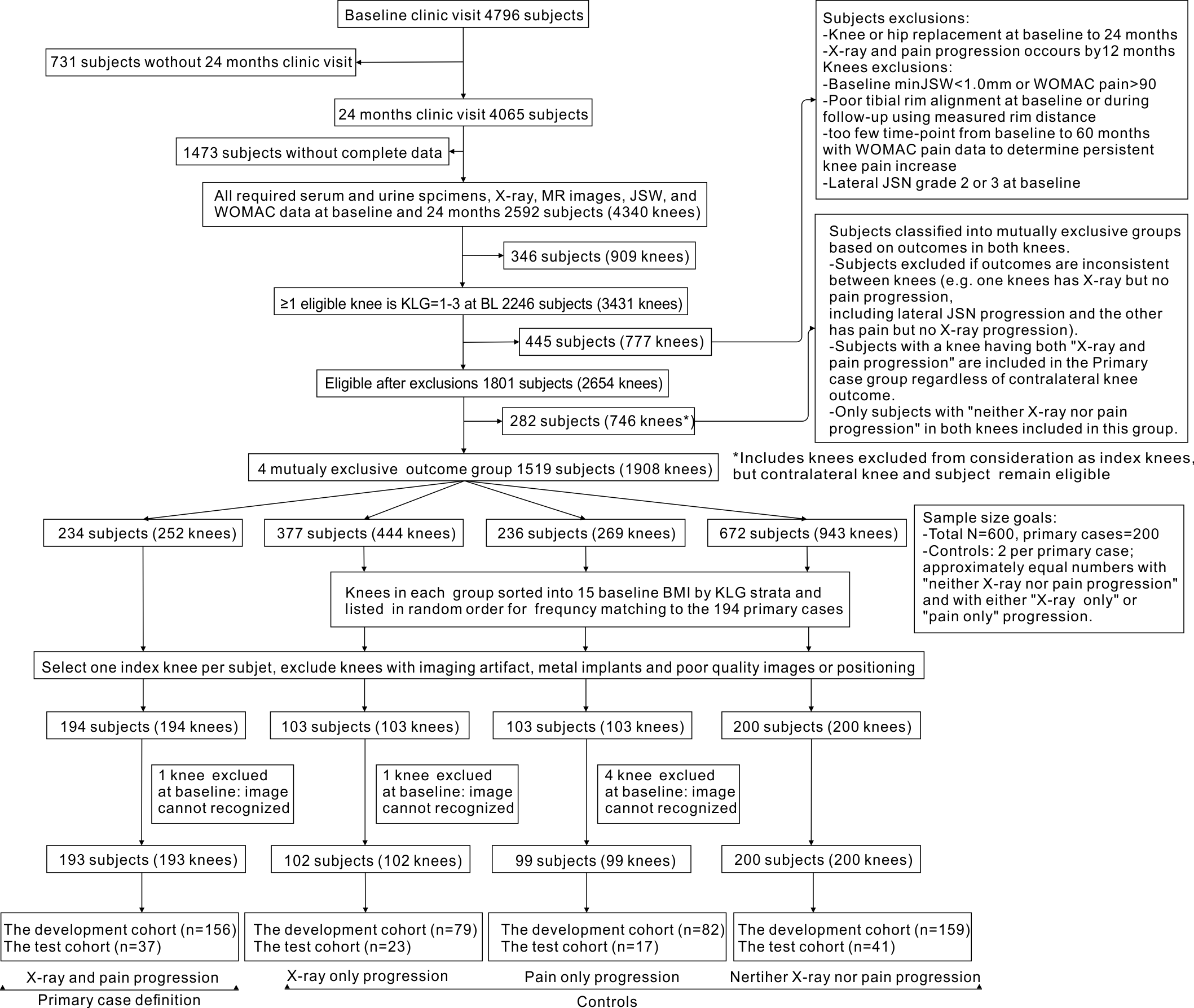

Supplement: S1 Fig — Flow chart of FNIH OA Biomarkers Consortium cohort study inclusion. FNIH OA Biomarkers Consortium cohort: Foundation of the NIH OsteoArthritis Biomarkers Consortium cohort, MR: Magnetic Resonance, JSW: Joint Space Width, WOMAC: Western Ontario and McMaster Universities Arthritis Index, KLG: Kellgren-Lawrence Grade, BMI: Body Mass Index, BL: Baseline. (TIF) [file pmed.1004665.s001.tif]

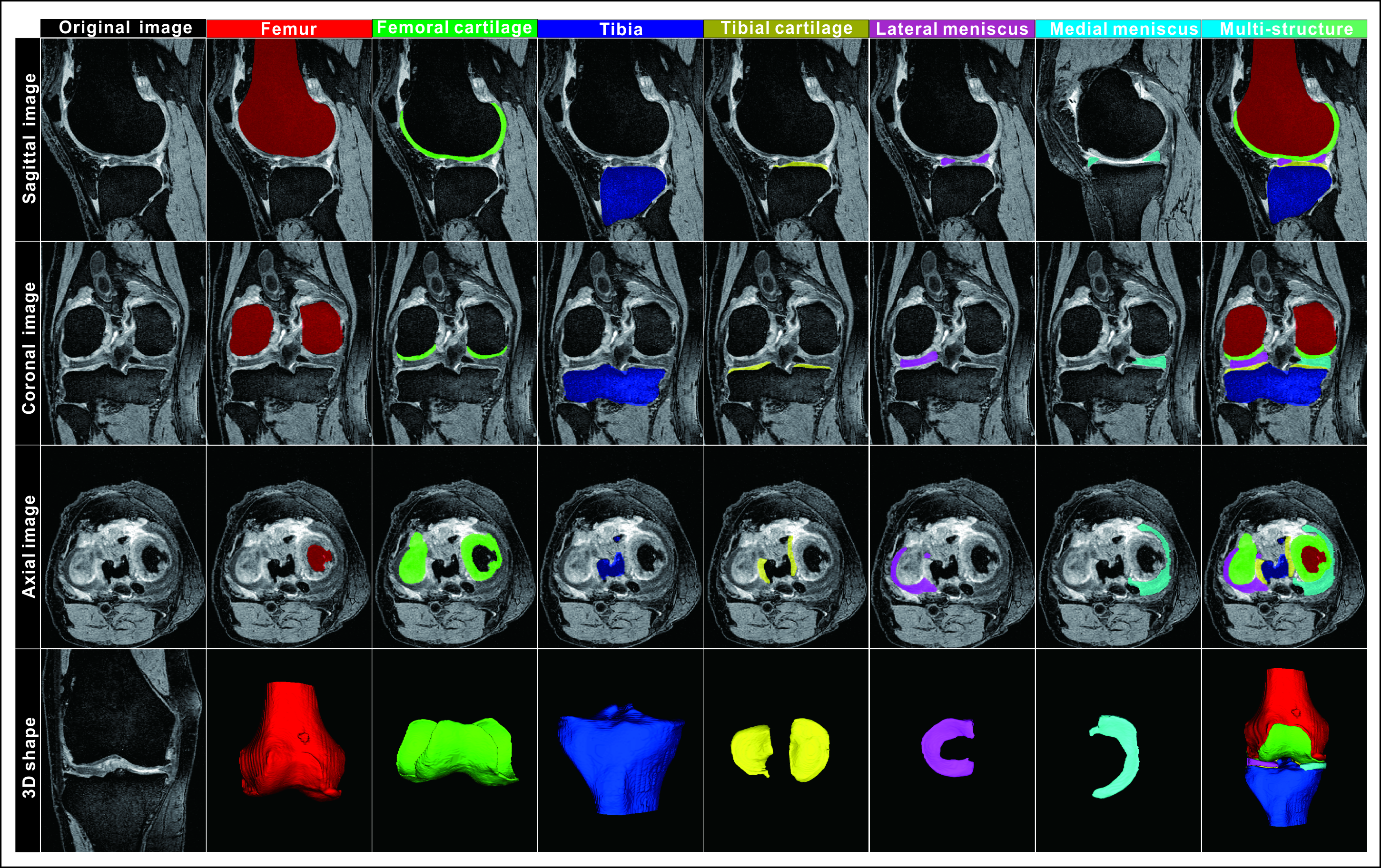

Supplement: S2 Fig — The MRI segmentation scheme in our study. MRI: Magnetic Resonance Image. (TIF) [file pmed.1004665.s002.tif]

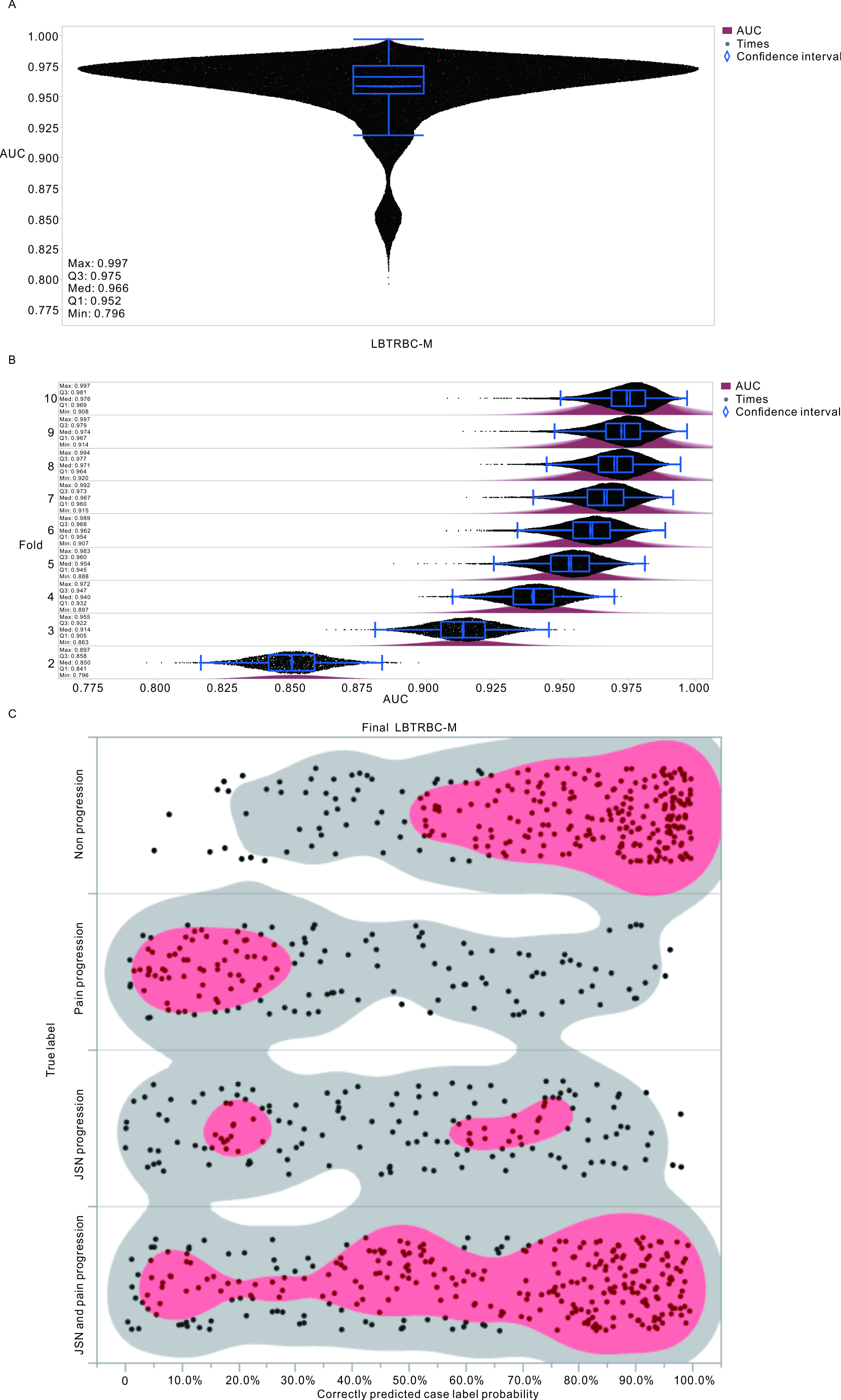

Supplement: S3 Fig — The results of 10-fold cross-validation to predict knee osteoarthritis progression and contour plot of predicted label probability under actual labels. The quartile of AUC was shown in A. The quartile of AUC in each fold was shown in B. Contour plot of predicted label probability under actual labels in final LBTRBC-M was shown in C. The 10-fold cross-validation were repeated 100 interactions. AUC: Area Under receiver operating characteristic Curve. JSN: Joint Space Narrowing, LBTRBC-M: Load-Bearing Tissue Radiomic plus Biochemical biomarker and Clinical variable Model. (TIF) [file pmed.1004665.s003.tif]

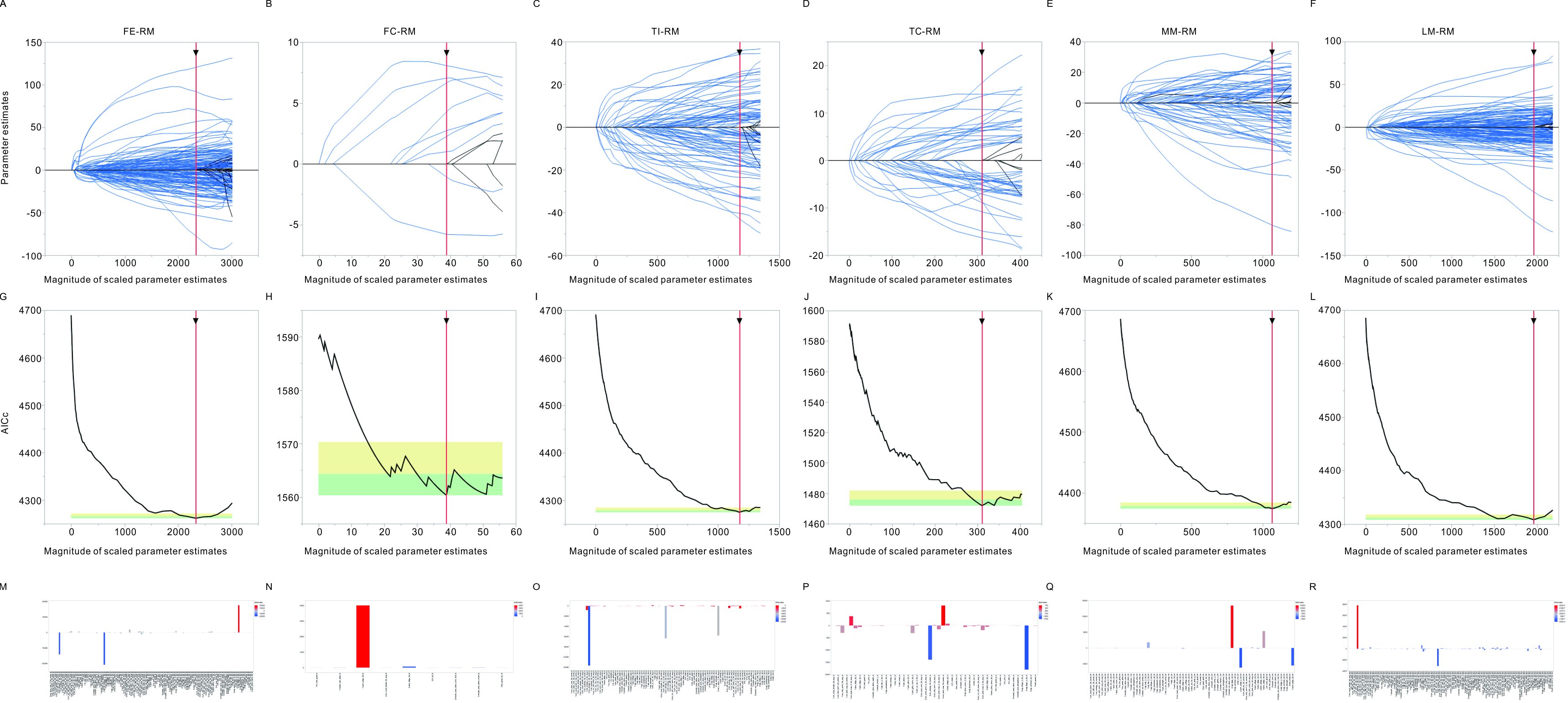

Supplement: S4 Fig — Feature selection process by LASSO regression in single-structure MRI radiomic models. Panel (A) to (F) show the magnitude of scaled parameter estimates for each model (FE-RM, FC-RM, TI-RM, TC-RM, LM-RM, and MM-RM), which indicate the importance of each MRI radiomic feature in predicting KOA progression. Panels (A), (B), (C), (D), (E), and (F) represent the magnitude of scaled parameter estimates for FE-RM, FC-RM, TI-RM, TC-RM, LM-RM, and MM-RM, respectively. Panels (G) to (L) present the scaled parameter estimates of the same models using the Akaike Information Criterion (AICc) for feature selection, providing an additional measure of model performance and fit. Panels (M) to (R) illustrate the weight of features for each model, showing the relative contribution of each selected feature to the overall predictive power of the model. These results highlight the most influential features in each MRI radiomic model for predicting KOA progression. FE-RM: Femur Radiomic Model, FC-RM: Femoral Cartilage Radiomic Model, TI-RM: Tibia Radiomic Model, TC-RM: Tibial Cartilage Radiomic Model, LM-RM: Lateral Meniscal Radiomic Model, MM-RM: Medial Meniscal Radiomic Model, AICc: Akaike Information Criterion, corrected. (TIF) [file pmed.1004665.s004.tif]

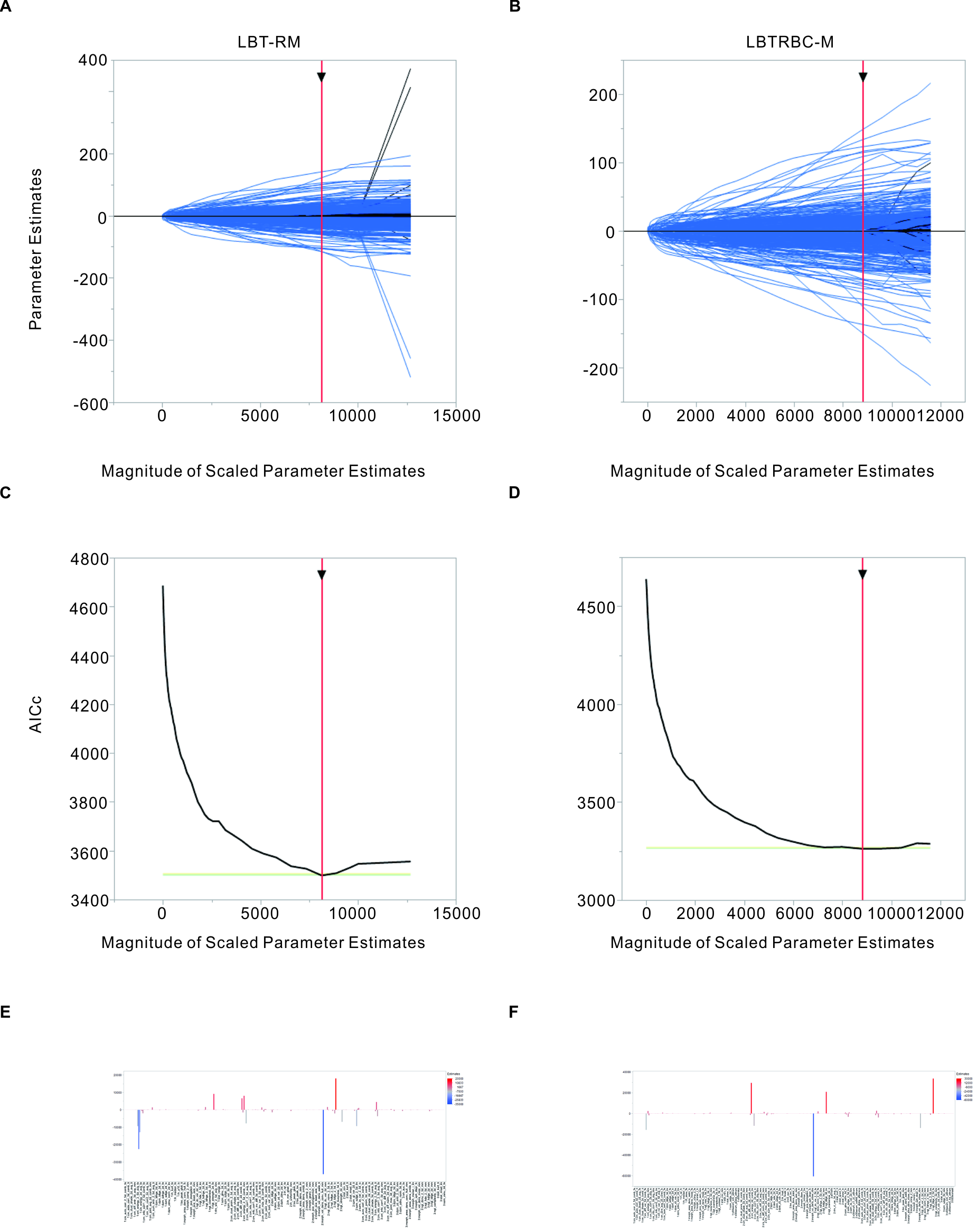

Supplement: S5 Fig — Feature selection process by LASSO regression in the LBT-RM and LBTRBC-M models. Panel (A) and (B) show the magnitude of scaled parameter estimates for the LBT-RM and the LBTRBC-M, respectively. Panels (C) and (D) represent the AICc-based selection of the most important features for both models, offering an alternative approach to assess the performance and fit of the models. Panels (E) and (F) show the weight of features in LBT-RM and LBTRBC-M, respectively, demonstrating how individual features contribute to the predictive power of each model. These visualizations offer a clearer understanding of the key features selected by LASSO regression and their impact on model performance for predicting KOA progression. LBT-RM: Load-Bearing Tissue Radiomic Model, LBTRBC-M: Load-Bearing Tissue Radiomic plus Biochemical Biomarker and Clinical Variable Model, AICc: Akaike Information Criterion, corrected. (TIF) [file pmed.1004665.s005.tif]

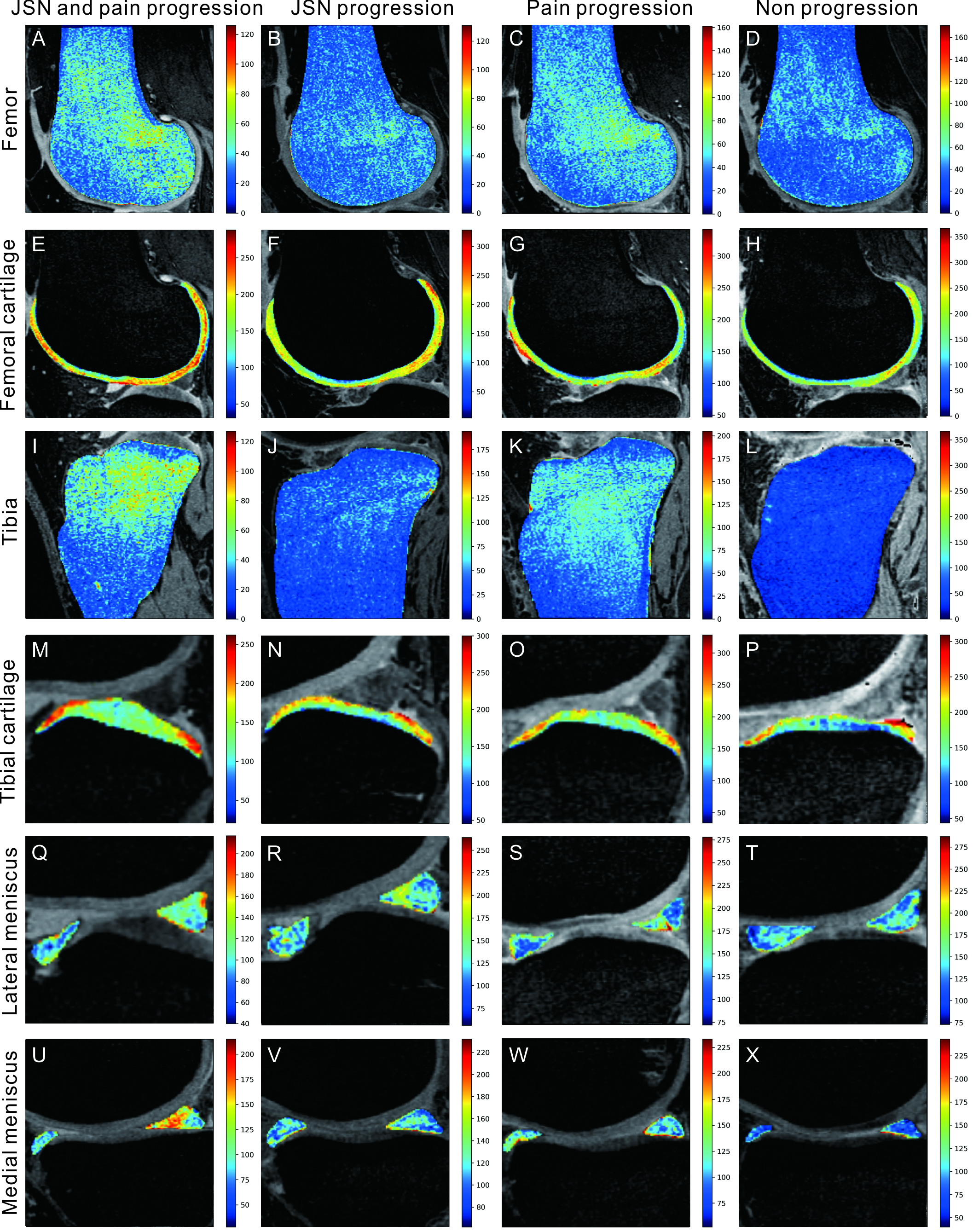

Supplement: S6 Fig — The DESS signal feature maps of load-bearing tissues in different groups. DESS signal intensity maps of femur (A–D), femoral cartilage (E–H), tibia (I–L), tibial cartilage (M–P), lateral meniscus (Q–T), medial meniscus (U–X) were developed in four groups. The high values of the femur and tibia were detected in JSN and pain progression, and pain progression group. The high value of femoral cartilage, tibial cartilage, lateral meniscus, and medial meniscus were detected in JSN and pain progression, and JSN progression group. JSN: Joint Space Narrowing, DESS: Double Echo Steady-State. (TIF) [file pmed.1004665.s006.tif]

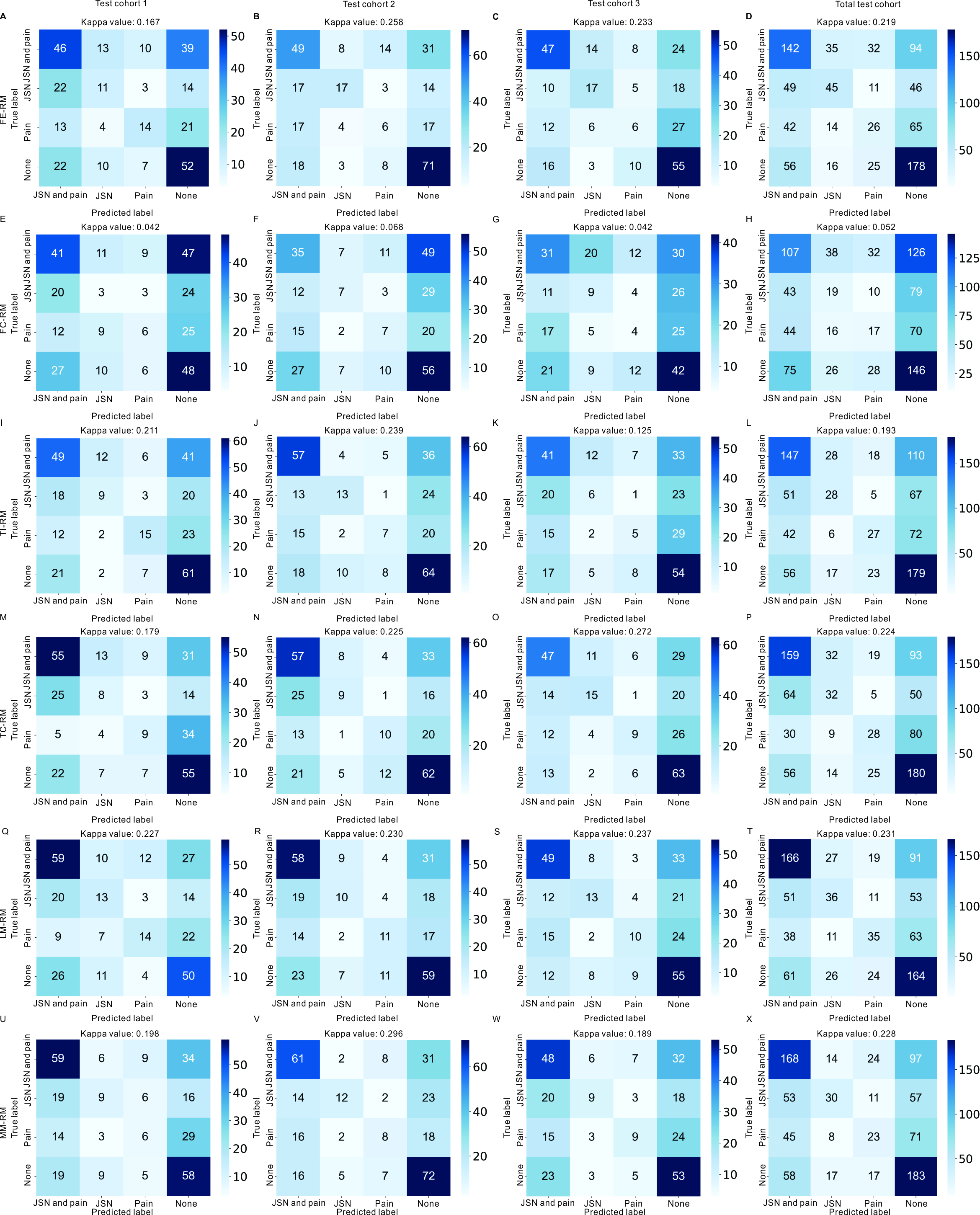

Supplement: S7 Fig — The confusion matrix results of single-structure MRI radiomic models in the test cohorts. The confusion matrix of FE-RM (A–D), FC-RM (E–H), TI-RM (I–L), TC-RM (M–P), LM-RM (Q–T), MM-RM (U–X) in the test cohort 1–3 and the total test cohort. The results of test cohort 1, test cohort 2, test cohort 3, and the total test cohort corresponded to baseline, 1-years follow-up, 2-year follow-up, and encompassed the aforementioned follow-up time points. FE-RM: Femur Radiomic Model, FC-RM: Femoral Cartilage Radiomic Model, TI-RM: Tibia Radiomic Model, TC-RM: Tibial Cartilage Radiomic Model, LM-RM: Lateral Meniscal Radiomic Model, MM-RM: Medial Meniscal Radiomic Model. (TIF) [file pmed.1004665.s007.tif]

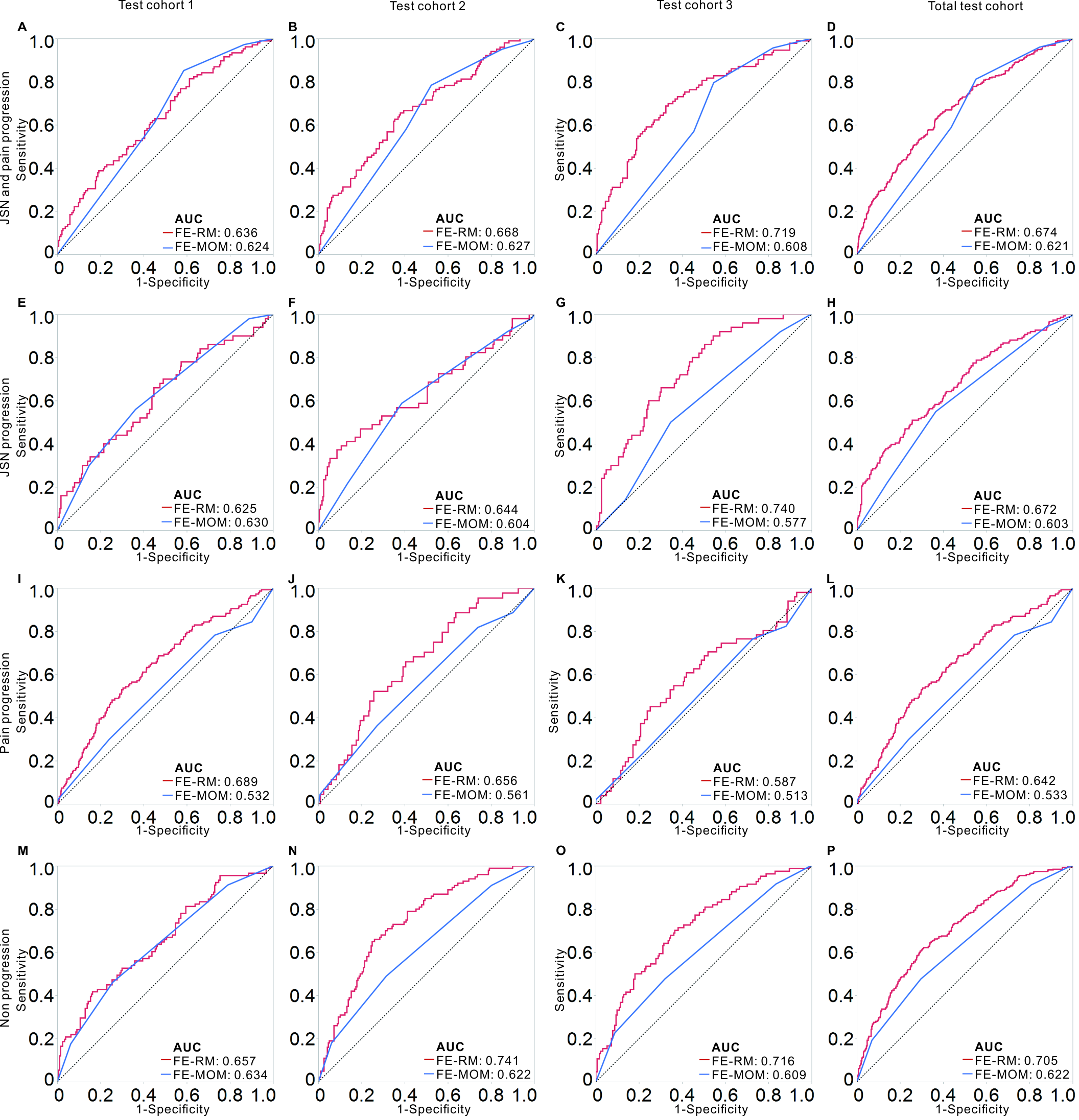

Supplement: S8 Fig — The comparations of AUC between FE-RM and FE-MOM in predicting KOA progression. The performance of predicting JSN and pain progression (A–D), JSN progression (E–H), pain progression (I–L), and non progression (M–P) in FE-RM and FE-MOM in the test cohort 1–3 and the total test cohort. The results of test cohort 1, test cohort 2, test cohort 3, and the total test cohort corresponded to baseline, 1-years follow-up, 2-year follow-up, and encompassed the aforementioned follow-up time points. FE-RM: Femur Radiomic Model, FE-MOM: Femur MOAKS Model, AUC: Area Under receiver operating characteristic Curve, MOAKS: Magnetic resonance imaging OsteoArthritis Knee Score. (TIF) [file pmed.1004665.s008.tif]

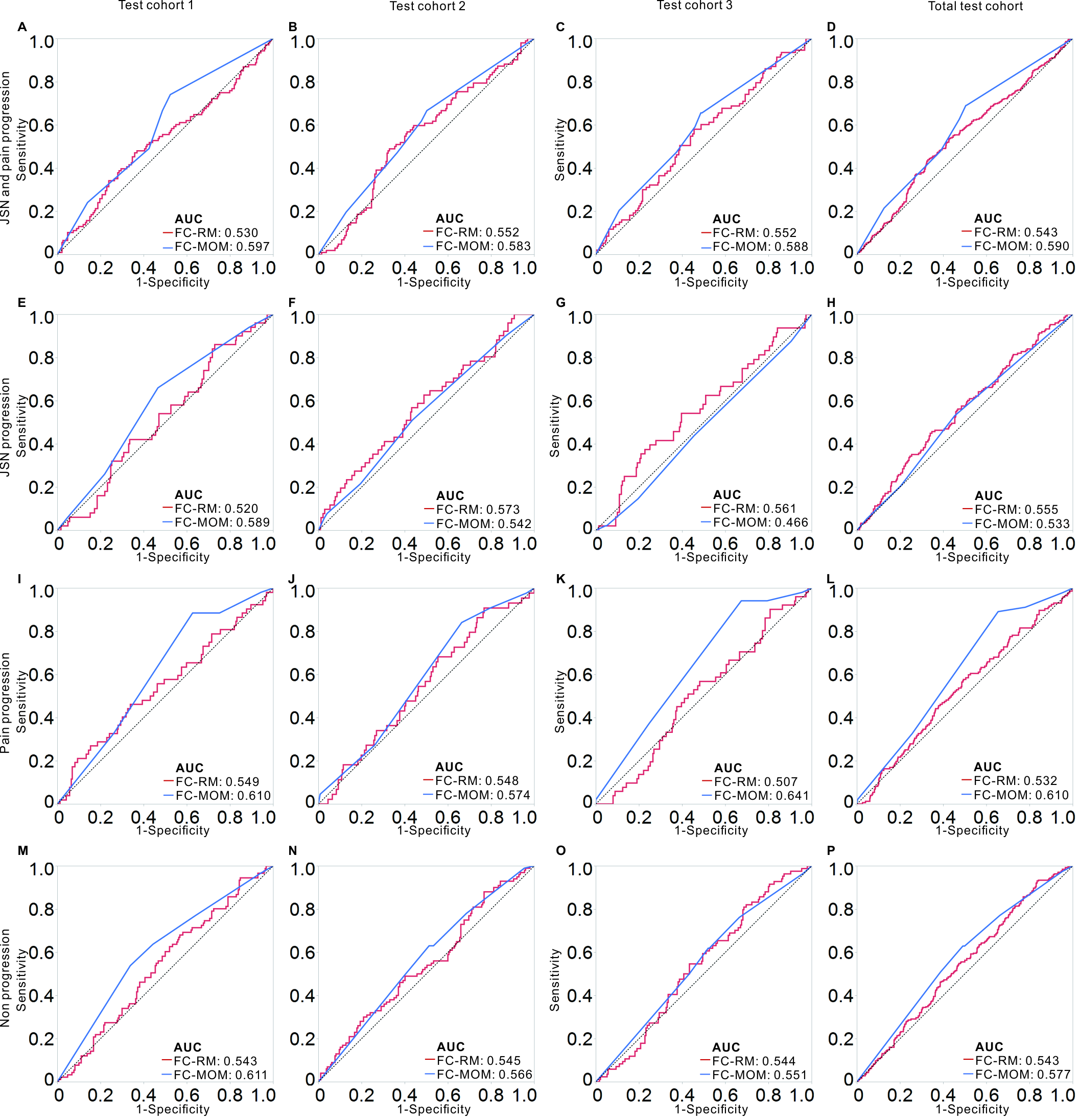

Supplement: S9 Fig — The comparations of AUC between FC-RM and FC-MOM in predicting KOA progression. The performance of predicting JSN and pain progression (A–D), JSN progression (E–H), pain progression (I–L), and non progression (M–P) in FC-RM and FC-MOM in the test cohort 1–3 and the total test cohort. The results of test cohort 1, test cohort 2, test cohort 3, and the total test cohort corresponded to baseline, 1-years follow-up, 2-year follow-up, and encompassed the aforementioned follow-up time points. FC-RM: Femoral Cartilage Radiomic Model, FC-MOM: Femoral Cartilage MOAKS Model, AUC: Area Under receiver operating characteristic Curve, MOAKS: Magnetic resonance imaging OsteoArthritis Knee Score. (TIF) [file pmed.1004665.s009.tif]

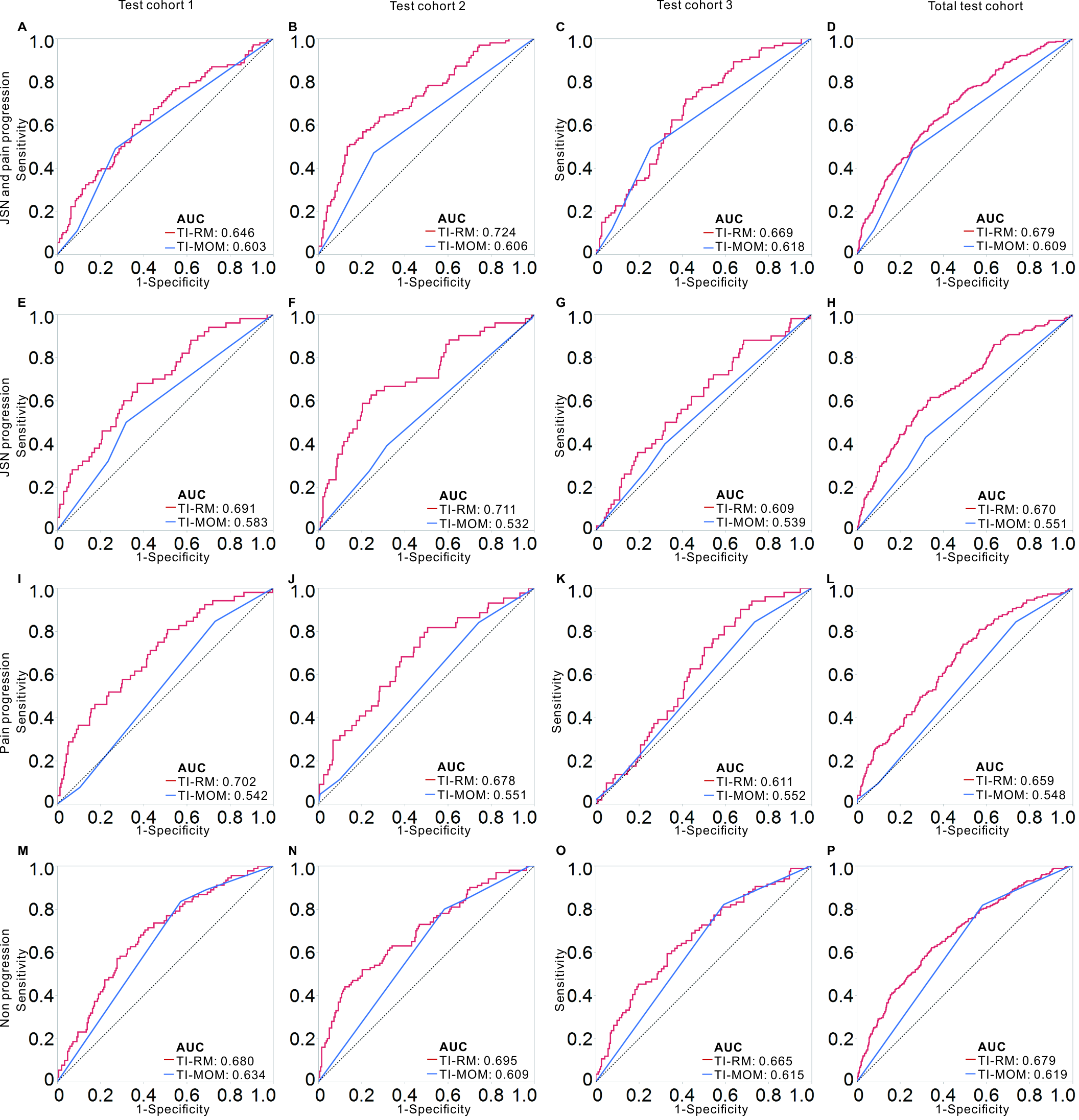

Supplement: S10 Fig — The comparations of AUC between TI-RM and TI-MOM in predicting KOA progression. The performance of predicting JSN and pain progression (A–D), JSN progression (E–H), pain progression (I–L), and non progression (M–P) in TI-RM and TI-MOM in the test cohort 1–3 and the total test cohort. The results of test cohort 1, test cohort 2, test cohort 3, and the total test cohort corresponded to baseline, 1-years follow-up, 2-year follow-up, and encompassed the aforementioned follow-up time points. TI-RM: Tibia Radiomic Model, TI-MOM: Tibia MOAKS model, AUC: Area Under receiver operating characteristic Curve, MOAKS: Magnetic resonance imaging OsteoArthritis Knee Score. (TIF) [file pmed.1004665.s010.tif]

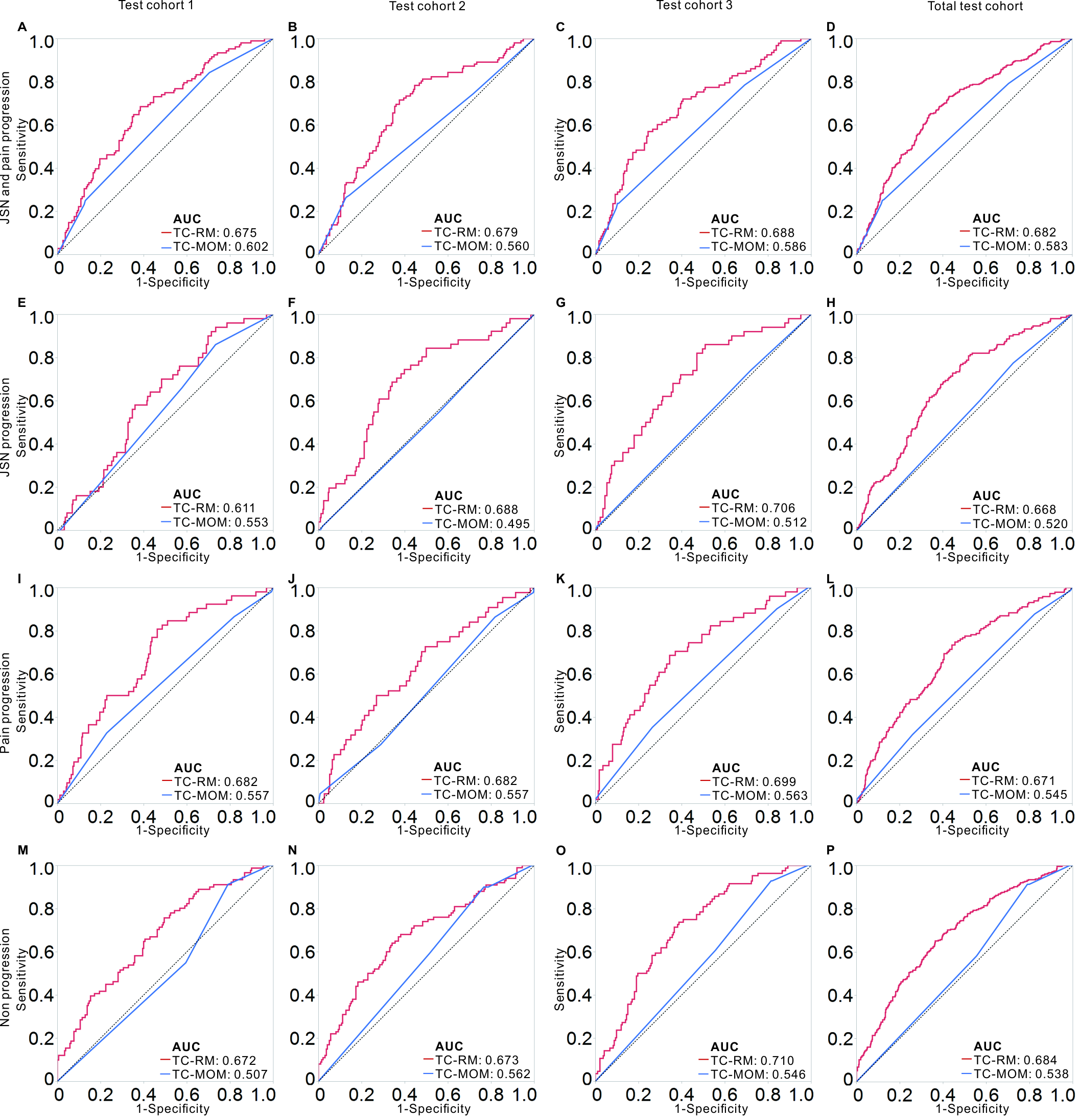

Supplement: S11 Fig — The comparations of AUC between TC-RM and TC-MOM in predicting KOA progression. The performance of predicting JSN and pain progression (A–D), JSN progression (E–H), pain progression (I–L), and non progression (M–P) in TC-RM and TC-MOM in the test cohort 1–3 and the total test cohort. The results of test cohort 1, test cohort 2, test cohort 3, and the total test cohort corresponded to baseline, 1-years follow-up, 2-year follow-up, and encompassed the aforementioned follow-up time points. TC-RM: Tibial Cartilage Radiomic Model, TC-MOM: Tibial Cartilage MOAKS model, AUC: Area Under receiver operating characteristic Curve, MOAKS: Magnetic resonance imaging OsteoArthritis Knee Score. (TIF) [file pmed.1004665.s011.tif]

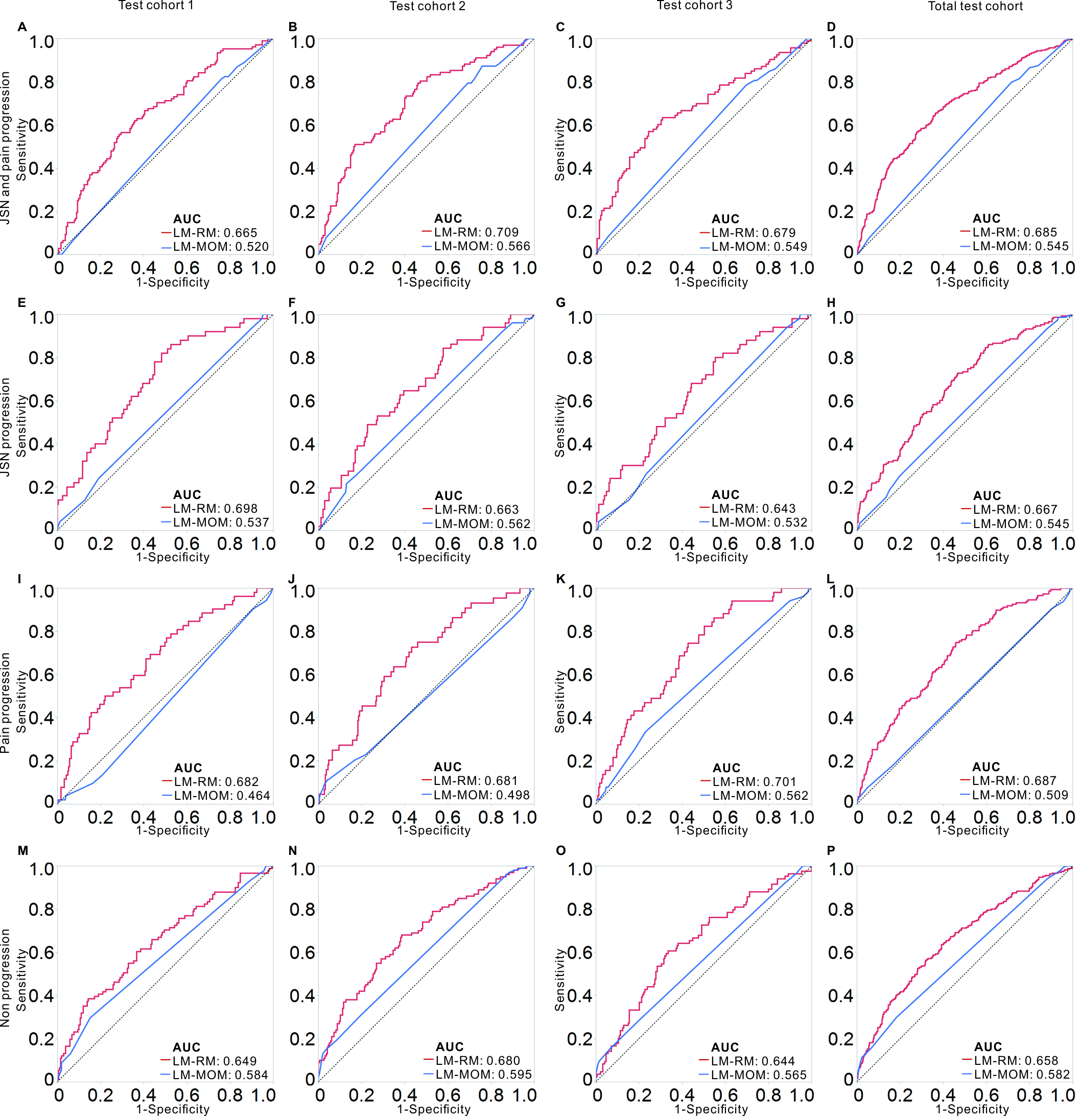

Supplement: S12 Fig — The comparations of AUC between LM-RM and LM-MOM in predicting KOA progression. The performance of predicting JSN and pain progression (A–D), JSN progression (E–H), pain progression (I–L), and non progression (M–P) in LM-RM and LM-MOM in the test cohort 1–3 and the total test cohort. The results of test cohort 1, test cohort 2, test cohort 3, and the total test cohort corresponded to baseline, 1-years follow-up, 2-year follow-up, and encompassed the aforementioned follow-up time points. LM-RM: Lateral meniscus radiomic model, LM-MOM: Lateral meniscus MOAKS model, AUC: Area Under receiver operating characteristic Curve, MOAKS: Magnetic resonance imaging OsteoArthritis Knee Score. (TIF) [file pmed.1004665.s012.tif]

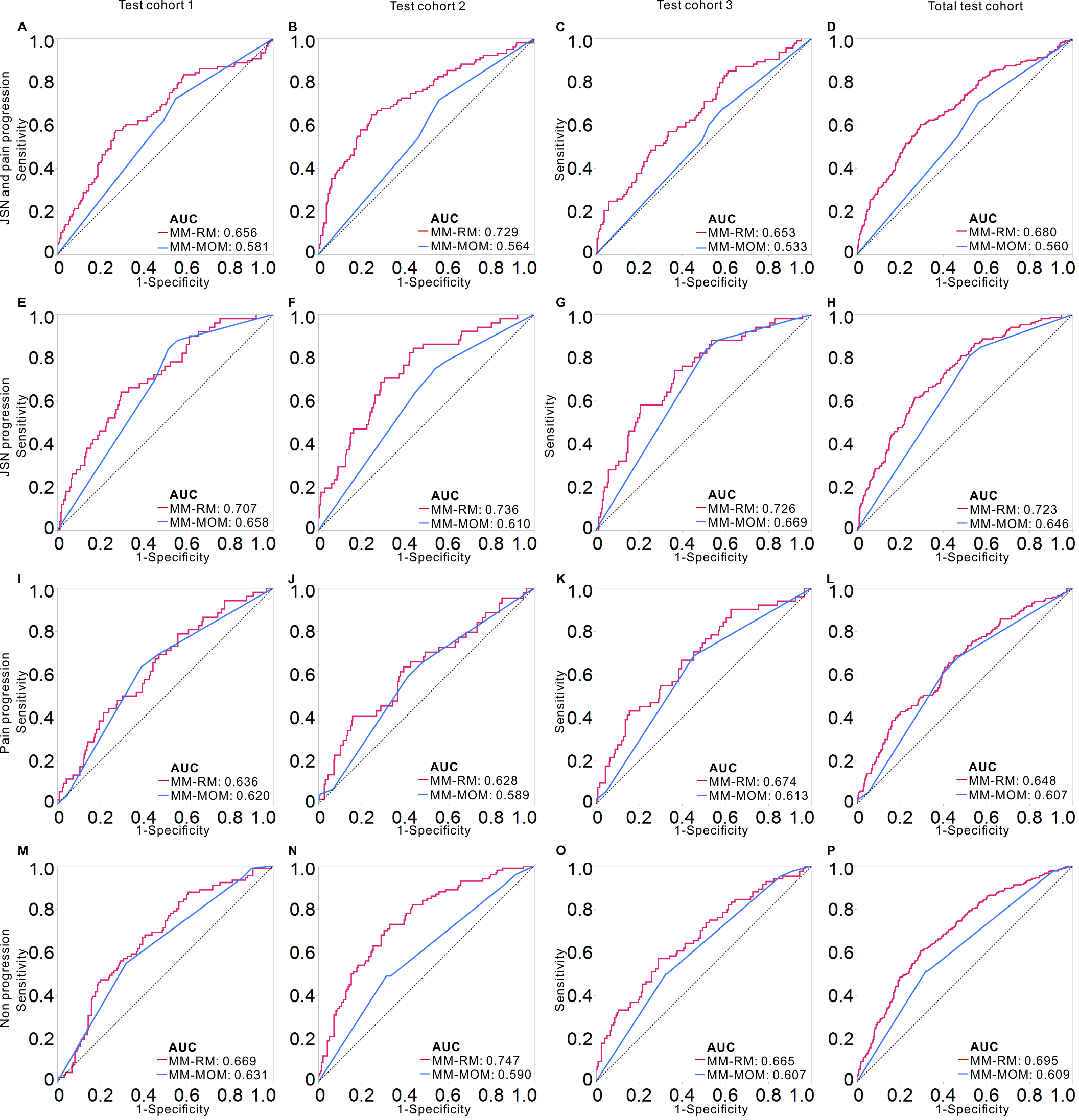

Supplement: S13 Fig — The comparations of AUC between MM-RM and MM-MOM in predicting KOA progression. The performance of predicting JSN and pain progression (A–D), JSN progression (E–H), pain progression (I–L), and non progression (M–P) in MM-RM and MM-MOM in the test cohort 1–3 and the total test cohort. The results of test cohort 1, test cohort 2, test cohort 3, and the total test cohort corresponded to baseline, 1-years follow-up, 2-year follow-up, and encompassed the aforementioned follow-up time points. MM-RM: Medial meniscus radiomic model, MM-MOM: Medial meniscus MOAKS model, AUC: Area Under receiver operating characteristic Curve, MOAKS: Magnetic resonance imaging OsteoArthritis Knee Score. (TIF) [file pmed.1004665.s013.tif]

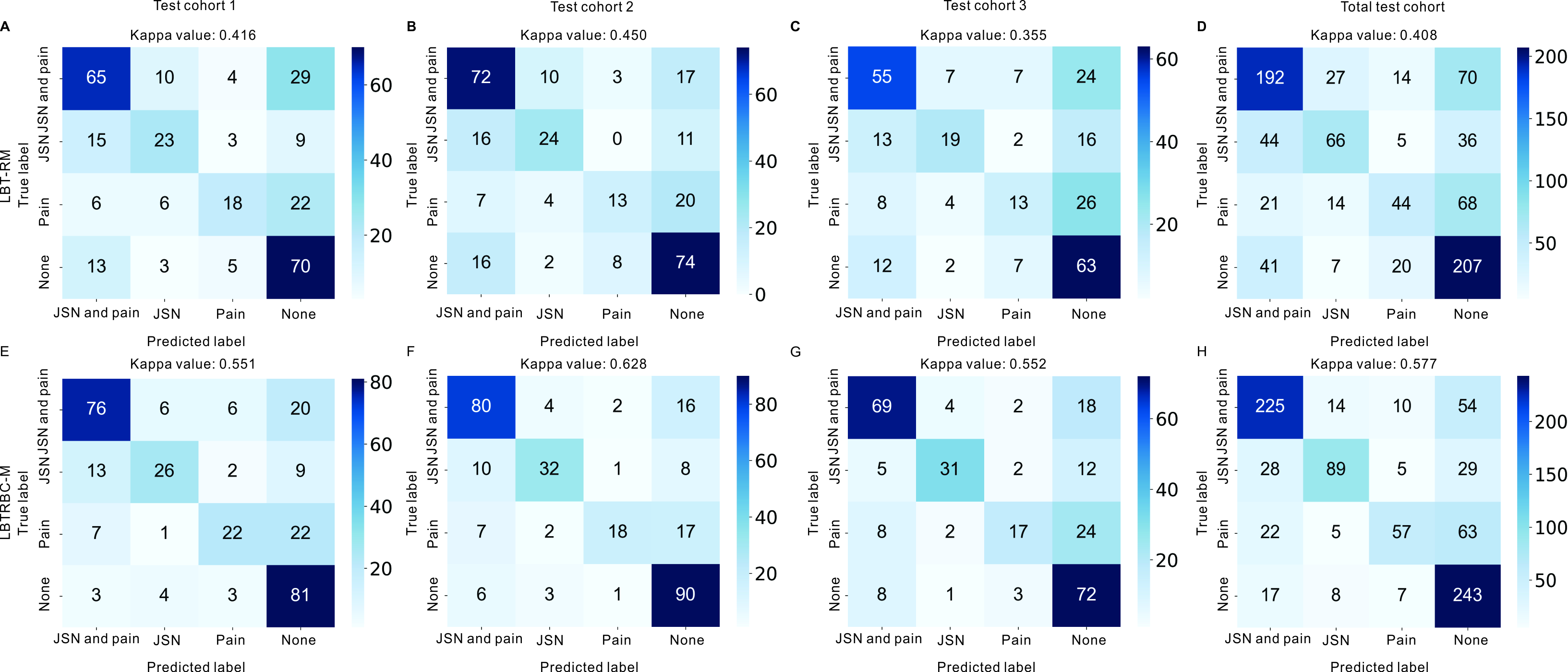

Supplement: S14 Fig — The confusion matrix results of load-bearing tissue MRI radiomic models in the test cohorts. The confusion matrix of LBT-RM (A–D) and LBTRBC-M (E–H) in the test cohort 1–4 and the total test cohort. The results of test cohort 1, test cohort 2, test cohort 3, and the total test cohort corresponded to baseline, 1-years follow-up, 2-year follow-up, and encompassed the aforementioned follow-up time points. LBT-RM: Load-Bearing Tissue Radiomic Model, LBTRBC-M: Load-Bearing Tissue Radiomic plus Biochemical biomarker and Clinical variable Model. (TIF) [file pmed.1004665.s014.tif]

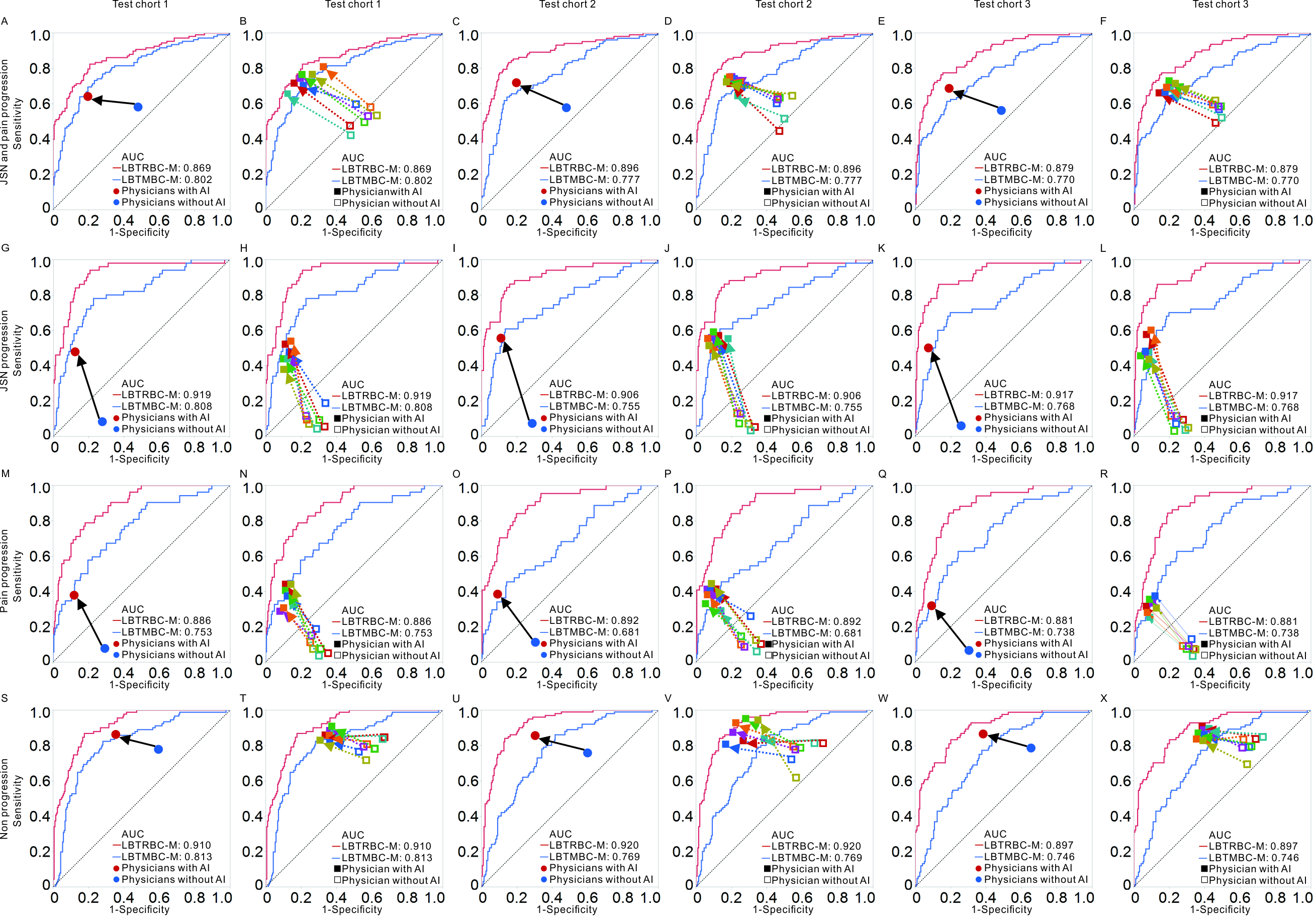

Supplement: S15 Fig — Performance of resident physicians and models in predicting the KOA progression in different time point test cohorts. AUC for predicting JSN and pain progression (A–F), JSN progression (G–L), pain progression (M–R), and non progression (S–X) among the LBTRBC-M, LBTMBC-M, and average performance of all resident physicians without (blue dot) and with (red dot) the support of LBTRBC-M in the test cohort 1–3 was demonstrated. As shown in A, C, E, G, I, K, M, O, Q, S, U, and W, both the sensitivity and specificity of resident physicians were improved when aid was supported by LBTRBC-M (black arrow) in the test cohort 1–3. As shown in B, D, F, H, J, L, N, P, R, T, V, and X, the individual performance of resident physicians was represented by open shapes (without LBTRBC-M aid) and filled shapes (with LBTRBC-M aid). The results of test cohort 1, test cohort 2, and test cohort 3 corresponded to baseline, 1-years follow-up, and 2-year follow-up time points. The colored dotted line of yellow, orange, purple, green, blue, teal, and red represented the predictive performance change of Liu, Zhao, Cao, J Li, Chen, X Wang, Dang, and M Zhang, respectively. AUC: Area Under receiver operating characteristic Curve, LBTRBC-M: Load-Bearing Tissue Radiomic plus Biochemical biomarker and Clinical variable Model, LBTMBC-M: Load-Bearing Tissue MOAKS plus Biochemical biomarker and Clinical variable Model, MOAKS: Magnetic resonance imaging OsteoArthritis Knee Score, AI: Artificial Intelligence. (TIF) [file pmed.1004665.s015.tif]

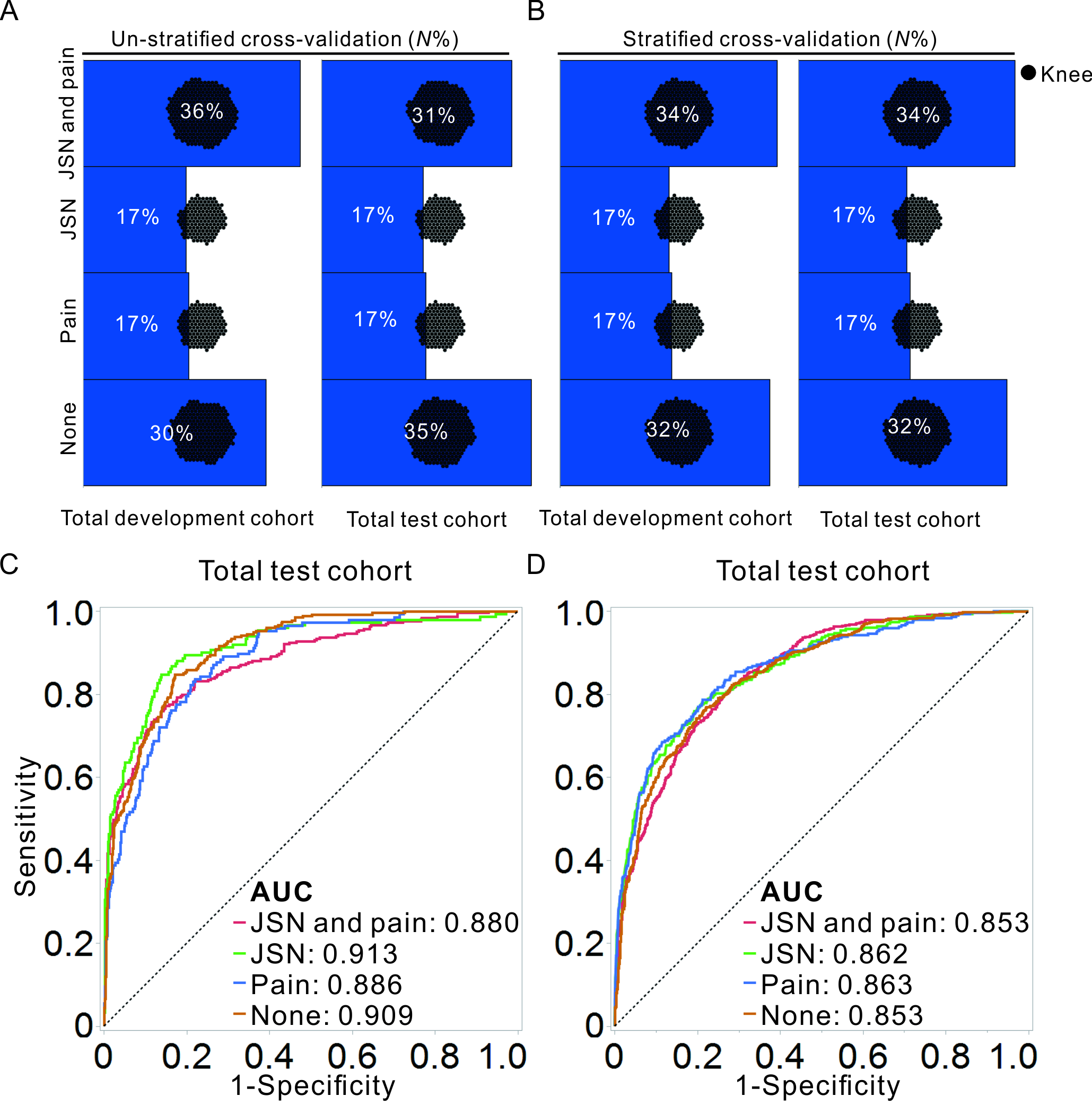

Supplement: S16 Fig — The predictive performance of the LBTRBC-M model using/without using the stratified cross-validation in the total test cohort. (A–B) We implemented a stratified cohort split for the LBTRBC-M model to ensure proportional representation of each KOA progression subtype, maintaining an approximate 2:1:1:2 ratio of JSN and pain progression, JSN progression, pain progression, and non-progression. (C–D) The AUC of LBTRBC-M model using/without using the stratified cross-validation was displayed in the total test cohort. AUC: Area Under receiver operating characteristic Curve, LBTRBC-M: Load-Bearing Tissue Radiomic plus Biochemical biomarker and Clinical variable Model, JSN: Joint Space Narrowing, KOA: Knee Osteoarthritis. (TIF) [file pmed.1004665.s016.tif]
