# Supplementary material for: Predicting knee osteoarthritis progression using neural network with longitudinal MRI radiomics, and biochemical biomarkers: A modeling study
Source: PLoS Med. 2025 Aug 21;22(8):e1004665. doi: 10.1371/journal.pmed.1004665 (PMC12370028; doi:10.1371/journal.pmed.1004665)
Supplement: S1 Table — MRI protocol details. (DOCX) [file pmed.1004665.s017.docx]

**Table S1. MRI Protocol Details.**

| **MRI parameters** | **SAG-3D-DESS-WE** |
| --- | --- |
| Pulse Sequence | de3d1 |
| Plane | Sagittal |
| Fat Sat | WE |
| Matrix (phase) | 307 |
| Matrix (freq) | 384 |
| No. of slices | 160 |
| FOV (mm) | 140 |
| Slice thickness (mm) | 0.7 |
| Skip (mm) | 0 |
| Flip Angle (deg) | 25 |
| TE/TI (ms) | 4.7 |
| TR (ms) | 16.3 |
| BW (Hz/pixel) | 183 |
| Chemical Shift | 0 |
| NAV (NEX) | 1 |
| Echo train length | 1 |
| Phase Encode Axis | A/P |
| Phase Partial Fourier | 1 |
| Readout Partial Fourier | 1 |
| Slice Partial Fourier | 0.75 |
| Distance Factor (%) | 20 |
| Phase Oversampling | 0 |
| Slice Oversampling | 10 |
| Phase Resolution | 80 |
| X-Resolution (mm) | 0.365 |
| Y-Resolution (mm) | 0.456 |
| Scan time (min) | 10.5 |

SAG-3D-DESS-WE: Sagittal-3-Dimensional Double Echo Steady-State with selective Water Excitation.
