# Supplementary material for: Predicting knee osteoarthritis progression using neural network with longitudinal MRI radiomics, and biochemical biomarkers: A modeling study
Source: PLoS Med. 2025 Aug 21;22(8):e1004665. doi: 10.1371/journal.pmed.1004665 (PMC12370028; doi:10.1371/journal.pmed.1004665)
Supplement: S2 Table — Baseline characteristics of participants in the development cohort 1 and test cohort 1. (DOCX) [file pmed.1004665.s018.docx]

**Table S2. Baseline characteristics of participants in the development cohort 1 and test cohort 1.**

|  | **Biological process indicate** | **Development cohort 1 (n=293)** | **Test cohort 1 (n=301)** | ***p* value** |
| --- | --- | --- | --- | --- |
| Age (year)^a^ | - | 62.2±8.9 | 61.0±8.9 | 0.114 |
| Female^b^ | - | 178 (61%) | 171 (57%) | 0.330 |
| BMI (kg/m^2^)^a^ | - | 30.5±4.6 | 30.9±4.9 | 0.362 |
| Pain medication^b^ | - | 90 (31%) | 85 (28%) | 0.508 |
| Knee injury^b^ | - | 95 (33%) | 117 (39%) | 0.101 |
| Knee surgery^b^ | - | 53 (18%) | 53 (18%) | 0.879 |
| KLG^c^ | - |  |  | 0.985 |
| 1 | - | 33 (11%) | 42 (14%) |  |
| 2 | - | 156 (53%) | 146 (49%) |  |
| 3 | - | 104 (36%) | 113 (37%) |  |
| Minimum JSW (mm)^a^ | - | 3.9±1.1 | 3.8±1.2 | 0.100 |
| WOMAC pain score^c^ | - | 1 (0, 4) | 1 (0, 4) | 0.899 |
| WOMAC stiffness score^c^ | - | 1 (0, 2) | 1 (0, 3) | 0.173 |
| WOMAC disability score^c^ | - | 3 (0, 12) | 5 (0, 16) | 0.753 |
| sCOMP (ng/mL)^a^ | Cartilage degradation | 839.6±736.7 | 764.1±300.1 | 0.101 |
| sHA (ng/mL)^a^ | Osteophyte burden, synovitis | 62.2±34.0 | 63.6±37.9 | 0.643 |
| sPⅡANP (ng/mL)^a^ | Type II collagen synthesis | 2620.6±763.3 | 2664.0±758.4 | 0.488 |
| sCTXⅠ (ng/mL)^a^ | Bone resorption | 0.4±0.2 | 0.4±0.2 | 0.358 |
| sCS846 (ng/mL)^a^ | Cartilage aggrecan synthesis/turnover | 110.4±35.4 | 121.8±131.8 | 0.155 |
| sMMP-3 (ng/mL)^a^ | Total (active and inactive) metalloprotease involved with joint tissue degradation | 17.8±17.3 | 18.0±13.1 | 0.889 |
| sC2C (ng/mL)^a^ | Type II collagen degradation | 207.9±49.7 | 212.7±56.1 | 0.278 |
| sC1, 2C (ng/mL)^a^ | Types I and II collagen degradation | 0.4±0.1 | 0.4±0.1 | 0.950 |
| sCPⅡ (pg/mL)^a^ | Type II collagen synthesis | 938.5±383.8 | 965.0±461.6 | 0.450 |
| sNTXⅠ (nmol BCE)^a^ | Bone resorption | 15.1±4.7 | 15.2±5.7 | 0.838 |
| sColl2_1_NO2 (nM)^a^ | Type II collagen degradation and inflammation | 9.0±5.2 | 9.1±7.5 | 0.833 |
| uCTXⅠ-α (ng/mL)^a^ | Turnover of newly formed bone | 0.7±0.6 | 0.7±0.7 | 0.553 |
| uCTXⅠ-β (ug/L)^a^ | bone resorption | 2.6±1.7 | 2.6±1.9 | 0.982 |
| uNTXⅠ (nM BCE)^a^ | Bone resorption | 33.0±16.2 | 33.7±19.0 | 0.636 |
| uC2C (pg/mL)^a^ | Type II collagen degradation | 163.6±86.2 | 165.6±109.8 | 0.802 |
| uC1, 2C (ug/mL)^a^ | Types I and II collagen degradation | 0.02±0.01 | 0.02±0.01 | 0.845 |
| uColl2_1_NO2 (nM)^a^ | Type II collagen degradation and inflammation | 0.02±0.01 | 0.03±0.02 | 0.063 |
| uCTXⅡ (ug/L)^a^ | Type II collagen degradation | 331.6±208.5 | 331.1±213.3 | 0.978 |

Data are mean±SD, number (%), or median (interquartile range).

^a^ unpaired t-tests are used for differences between means.

^b^ χ2 tests are used for differences between proportions.

^c^ Mann Whitney tests are used for differences between ranks.

The results of development cohort 1 and test cohort 1 corresponded to baseline follow-up. BMI: Body Mass Index, KLG: Kellgren and Lawrence Grade, JSW: Joint Space Width, WOMAC: the Western Ontario and McMaster Universities Arthritis Index, SD: Standard Deviation, sCOMP: serum Cartilage Oligomeric Matrix Protein, sHA: serum Hyaluronic Acid, sPⅡANP: serum type IIA Procollagen Amino terminal Propeptide, sCTXⅠ: serum type I collagen C-terminal Telopeptide, sCS846: serum aggrecan Chondroitin Sulfate 846 epitope, sMMP-3: serum Matrix MetalloProteinase-3, sC2C: serum Cleavage neoepitope of type II Collagen, sC1, 2C: serum type II Collagen neoepitope, sCPⅡ: serum C-Propeptide of type II collagen, sNTXⅠ: serum N-terminal Telopeptide of type I collagen, sColl2_1_NO2: serum triple helix of type II Collagen, uCTXⅠ-α: urine C-terminal cross-linked Telopeptide of type I collagen-α, uCTXⅠ-β: urine urine C-terminal cross-linked Telopeptide of type I collagen-β, uNTXⅠ: urine N-terminal cross-linked Telopeptide of type I collagen, uC2C: urine Cleavage neoepitope of type II Collagen, uC1, 2C: urine type II Collagen neoepitope, uColl2_1_NO2: urine triple helix of type II Collagen, uCTXⅡ: urine C-telopeptide fragment of type II collagen.
