# Supplementary material for: Predicting knee osteoarthritis progression using neural network with longitudinal MRI radiomics, and biochemical biomarkers: A modeling study
Source: PLoS Med. 2025 Aug 21;22(8):e1004665. doi: 10.1371/journal.pmed.1004665 (PMC12370028; doi:10.1371/journal.pmed.1004665)
Supplement: S3 Table — Baseline biochemical biomarker levels of participants in the development cohort 1 and test cohort 1. (DOCX) [file pmed.1004665.s019.docx]

**Table S3. Baseline biochemical biomarker levels of participants in the development cohort 1 and test cohort 1.**

|  | **Development cohort 1 (n=293)** | | | | |  | **Test cohort 1 (n=301)** | | | | |
| --- | --- | --- | --- | --- | --- | --- | --- | --- | --- | --- | --- |
| **Biomarkers (unit)** | **JSN and pain (n=85)** | **JSN (n=52)** | **Pain (n=47)** | **Non (n=109)** | ***p* value** |  | **JSN and pain (n=108)** | **JSN (n=50)** | **Pain (n=52)** | **Non (n=91)** | ***p* value** |
| sCOMP (ng/mL) | 844.3±480.6 | 975.5±1547.0 | 770.3±293.4 | 801.1±329.4 | 0.480 |  | 732.9±281.5 | 791.5±305.0 | 763.9±296.7 | 785.9±321.3 | 0.564 |
| sHA (ng/mL) | 64.3±30.8 | 60.9±26.1 | 69.3±56.7 | 58.2±25.7 | 0.265 |  | 61.9±24.7 | 62.4±23.9 | 68.1±52.9 | 63.7±46.4 | 0.808 |
| sPⅡANP (ng/mL) | 2540.5±836.2 | 2554.5±730.7 | 2619.1±696.1 | 2715.2±746.1 | 0.393 |  | 2612.1±745.7 | 2524.3±718.1 | 2644.1±686.1 | 2813.0±819.5 | 0.124 |
| sCTXⅠ (ng/mL) | 0.4±0.2 | 0.4±0.2 | 0.4±0.3 | 0.4±0.2 | 0.344 |  | 0.4±0.2 | 0.4±0.2 | 0.4±0.3 | 0.4±0.2 | 0.791 |
| sCS846 (ng/mL) | 109.5±38.7 | 111.7±42.2 | 109.9±27.9 | 110.8±32.3 | 0.985 |  | 115.1±47.7 | 103.4±13.1 | 124.7±96.6 | 138.0±221.7 | 0.452 |
| sMMP-3 (ng/mL) | 17.0±10.4 | 19.7±14.5 | 15.6±9.8 | 18.5±24.1 | 0.641 |  | 18.0±11.9 | 21.2±18.4 | 17.3±10.5 | 16.6±12.4 | 0.244 |
| sC2C (ng/mL) | 211.0±54.8 | 200.6±36.7 | 201.1±50.6 | 212.0±50.4 | 0.383 |  | 217.2±70.9 | 215.6±51.9 | 209.8±40.3 | 207.4±45.5 | 0.620 |
| sC1, 2C (ng/mL) | 0.4±0.2 | 0.4±0.1 | 0.4±0.1 | 0.4±0.1 | 0.610 |  | 0.4±0.1 | 0.4±0.1 | 0.4±0.1 | 0.4±0.2 | 0.918 |
| sCPⅡ (pg/mL) | 917.3±320.0 | 870.9±312.1 | 975.7±427.7 | 971.3±436.5 | 0.377 |  | 967.7±395.2 | 866.2±250.5 | 1042.1±518.2 | 971.9±573.3 | 0.289 |
| sNTXⅠ (nmol BCE) | 16.0±4.6 | 14.1±3.9 | 15.4±4.6 | 14.8±5.0 | 0.119 |  | 15.1±5.1 | 15.6±5.1 | 14.5±4.5 | 15.4±7.2 | 0.782 |
| sColl2_1_NO2 (nM) | 9.2±5.6 | 8.4±4.1 | 8.6±4.0 | 9.2±5.7 | 0.747 |  | 8.8±4.4 | 8.1±3.7 | 9.2±6.8 | 9.9±11.3 | 0.535 |
| uCTXⅠ-α (ng/mL) | 0.9±0.8 | 0.6±0.4 | 0.6±0.4 | 0.7±0.6 | 0.054 |  | 0.7±0.5 | 0.6±0.4 | 0.8±1.2 | 0.7±0.6 | 0.537 |
| uCTXⅠ-β (ug/L) | 3.0±2.0 | 2.4±1.3 | 2.5±1.4 | 2.5±1.7 | 0.110 |  | 2.6±1.5 | 2.5±1.4 | 2.8±2.9 | 2.6±1.8 | 0.874 |
| uNTXⅠ (nM BCE) | 35.3±15.0 | 29.5±11.9 | 35.1±14.7 | 32.0±19.1 | 0.147 |  | 34.6±17.8 | 33.4±13.1 | 34.6±27.0 | 32.4±17.9 | 0.857 |
| uC2C (pg/mL) | 175.3±90.5 | 153.9±73.0 | 171.9±102.3 | 155.5±80.7 | 0.306 |  | 171.1±104.6 | 174.0±80.2 | 176.4±181.8 | 148.3±67.1 | 0.344 |
| uC1, 2C (ug/mL) | 0.02±0.02 | 0.02±0.01 | 0.02±0.01 | 0.02±0.01 | 0.602 |  | 0.02±0.01 | 0.02±0.01 | 0.02±0.02 | 0.02±0.01 | 0.310 |
| uColl2_1_NO2 (nM) | 0.02±0.02 | 0.02±0.01 | 0.02±0.01 | 0.02±0.01 | 0.762 |  | 0.02±0.01 | 0.03±0.02 | 0.03±0.03 | 0.03±0.02 | 0.564 |
| uCTXⅡ (ug/L) | 377.3±234.5 | 304.1±248.1 | 349.1±183.4 | 301.4±168.2 | 0.054 |  | 353.4±212.1 | 361.1±198.8 | 311.8±300.7 | 299.1±152.0 | 0.200 |

Data are mean±SD and one-way ANOVA tests are used for differences between means.

The results of development cohort 1 and test cohort 1 corresponded to baseline follow-up. JSN: Joint Space Narrowing, SD: Standard Deviation, ANOVA: ANalysis Of VAriance, sCOMP: serum Cartilage Oligomeric Matrix Protein, sHA: serum Hyaluronic Acid, sPⅡANP: serum type IIA Procollagen Amino terminal Propeptide, sCTXⅠ: serum type I collagen C-terminal Telopeptide, sCS846: serum aggrecan Chondroitin Sulfate 846 epitope, sMMP-3: serum Matrix MetalloProteinase-3, sC2C: serum Cleavage neoepitope of type II Collagen, sC1, 2C: serum type II Collagen neoepitope, sCPⅡ: serum C-Propeptide of type II collagen, sNTXⅠ: serum N-terminal Telopeptide of type I collagen, sColl2_1_NO2: serum triple helix of type II Collagen, uCTXⅠ-α: urine C-terminal cross-linked Telopeptide of type I collagen-α, uCTXⅠ-β: urine urine C-terminal cross-linked Telopeptide of type I collagen-β, uNTXⅠ: urine N-terminal cross-linked Telopeptide of type I collagen, uC2C: urine Cleavage neoepitope of type II Collagen, uC1, 2C: urine type II Collagen neoepitope, uColl2_1_NO2: urine triple helix of type II Collagen, uCTXⅡ: urine C-telopeptide fragment of type II collagen.
