# Supplementary material for: Predicting knee osteoarthritis progression using neural network with longitudinal MRI radiomics, and biochemical biomarkers: A modeling study
Source: PLoS Med. 2025 Aug 21;22(8):e1004665. doi: 10.1371/journal.pmed.1004665 (PMC12370028; doi:10.1371/journal.pmed.1004665)
Supplement: S4 Table — DSCs for the CNNs automated segmentation and manual adjustment segmentation. (DOCX) [file pmed.1004665.s020.docx]

**Table S4. DSCs for the CNNs automated segmentation and manual adjustment segmentation.**

| **Segmentation** | **DSCs** | |
| --- | --- | --- |
|  | **Reader 1 (n=20)** | **Reader 2 (n=20)** |
| Femur | 0.832±0.014 | 0.823±0.016 |
| Femoral cartilage | 0.807±0.012 | 0.812±0.011 |
| Tibia | 0.823±0.034 | 0.831±0.005 |
| Tibial cartilage | 0.811±0.004 | 0.814±0.021 |
| Lateral meniscus | 0.807±0.005 | 0.827±0.004 |
| Medial meniscus | 0.841±0.012 | 0.810±0.006 |

Data are mean±SD.

DSCs: Dice Similarity Coefficients, CNNs: Convolutional Neural Networks, SD: Standard Deviation.

DSCs were good (>0.800).
