# Supplementary material for: Predicting knee osteoarthritis progression using neural network with longitudinal MRI radiomics, and biochemical biomarkers: A modeling study
Source: PLoS Med. 2025 Aug 21;22(8):e1004665. doi: 10.1371/journal.pmed.1004665 (PMC12370028; doi:10.1371/journal.pmed.1004665)
Supplement: S5 Table — Selected features of predictive models in total development cohort. (DOCX) [file pmed.1004665.s021.docx]

**Table S5. Selected features of predictive models in total development cohort.**

| **Femur Radiomic Model (n=101)**‡ |
| --- |
| dzm_lde_2_5d_FE |
| morph_diam_FE |
| ih_skew_FE |
| morph_pca_flatness_FE |
| cm_info_corr1_2_5d_comb_FE |
| ih_kurt_FE |
| morph_area_dens_conv_hull_FE |
| ngl_dcnu_2d_FE |
| ngt_complexity_2d_FE |
| szm_lze_2d_FE |
| ngl_dc_var_3d_FE |
| morph_comp_2_FE |
| dzm_sde_2d_FE |
| dzm_zdnu_norm_3d_FE |
| morph_geary_c_FE |
| cm_clust_prom_2_5d_avg_FE |
| morph_vol_dens_mvee_FE |
| cm_joint_var_2_5d_comb_FE |
| ngl_dcnu_norm_2_5d_FE |
| szm_zsnu_2d_FE |
| ngl_hde_2_5d_FE |
| cm_inv_diff_mom_2_5d_comb_FE |
| cm_info_corr2_2d_avg_FE |
| szm_szhge_3d_FE |
| cm_info_corr2_3d_comb_FE |
| cm_diff_var_2d_avg_FE |
| ngl_ldhge_2_5d_FE |
| ih_iqr_FE |
| ngt_strength_2d_FE |
| cm_energy_2d_avg_FE |
| cm_clust_shade_3d_comb_FE |
| cm_info_corr1_2_5d_avg_FE |
| cm_auto_corr_2_5d_comb_FE |
| cm_clust_prom_3d_comb_FE |
| ngl_dc_energy_2_5d_FE |
| stat_median_FE |
| ngl_lde_2_5d_FE |
| cm_clust_prom_2d_comb_FE |
| dzm_ldhge_3d_FE |
| ngl_hdhge_2d_FE |
| ngl_dc_var_2_5d_FE |
| morph_area_dens_aabb_FE |
| morph_vol_dens_aabb_FE |
| szm_zs_var_2_5d_FE |
| cm_inv_var_3d_comb_FE |
| ih_p10_FE |
| stat_skew_FE |
| ih_rmad_FE |
| cm_sum_var_2d_comb_FE |
| cm_auto_corr_2d_comb_FE |
| rlm_rl_var_2d_comb_FE |
| szm_szhge_2_5d_FE |
| cm_info_corr1_2d_comb_FE |
| dzm_ldlge_2d_FE |
| cm_inv_diff_2d_avg_FE |
| cm_energy_2_5d_avg_FE |
| szm_zsnu_norm_3d_FE |
| dzm_ldlge_2_5d_FE |
| ngl_hdlge_3d_FE |
| cm_sum_entr_2d_comb_FE |
| cm_joint_max_3d_avg_FE |
| szm_szlge_2d_FE |
| stat_qcod_FE |
| morph_area_dens_aee_FE |
| cm_info_corr1_2d_avg_FE |
| cm_clust_shade_2d_avg_FE |
| stat_range_FE |
| cm_auto_corr_2d_avg_FE |
| ngl_ldlge_2_5d_FE |
| ih_min_grad_FE |
| loc_peak_glob_FE |
| morph_area_dens_ombb_FE |
| ngt_busyness_3d_FE |
| cm_diff_var_2d_comb_FE |
| cm_joint_max_3d_comb_FE |
| ngt_contrast_2_5d_FE |
| morph_pca_elongation_FE |
| cm_joint_max_2_5d_comb_FE |
| cm_inv_var_2d_avg_FE |
| ngl_dc_energy_2d_FE |
| dzm_ldhge_2_5d_FE |
| szm_zs_entr_2_5d_FE |
| ngl_hdlge_2_5d_FE |
| ivh_auc_FE |
| morph_area_dens_mvee_FE |
| dzm_ldhge_2d_FE |
| ngt_busyness_2d_FE |
| ih_max_grad_g_FE |
| ngl_glnu_2d_FE |
| cm_joint_max_2_5d_avg_FE |
| ih_max_grad_FE |
| ngl_hdlge_2d_FE |
| ngt_complexity_2_5d_FE |
| morph_moran_i_FE |
| ih_median_FE |
| stat_cov_FE |
| cm_energy_2d_comb_FE |
| loc_peak_loc_FE |
| cm_energy_2_5d_comb_FE |
| cm_info_corr2_2_5d_comb_FE |
| dzm_sdhge_2d_FE |
| **Femoral Cartilage Radiomic Model (n=9)**‡ |
| morph_sph_dispr_FC |
| szm_szlge_2d_FC |
| morph_area_dens_mvee_FC |
| loc_peak_loc_FC |
| ih_max_grad_FC |
| cm_clust_shade_2d_avg_FC |
| dzm_ldlge_3d_FC |
| ih_iqr_FC |
| morph_area_dens_conv_hull_FC |
| **Tibia Radiomic Model (n=74)**‡ |
| morph_pca_elongation_TI |
| stat_iqr_TI |
| ngt_busyness_3d_TI |
| loc_peak_glob_TI |
| cm_inv_diff_mom_norm_3d_avg_TI |
| cm_auto_corr_3d_comb_TI |
| loc_peak_loc_TI |
| morph_area_dens_mvee_TI |
| ih_median_TI |
| morph_vol_dens_aabb_TI |
| stat_kurt_TI |
| cm_inv_diff_2d_avg_TI |
| stat_min_TI |
| ngt_contrast_2_5d_TI |
| morph_pca_least_axis_TI |
| cm_sum_entr_2_5d_avg_TI |
| cm_inv_diff_mom_2_5d_avg_TI |
| cm_clust_prom_2_5d_comb_TI |
| cm_energy_2_5d_comb_TI |
| ngt_strength_2d_TI |
| cm_clust_shade_2_5d_comb_TI |
| cm_energy_2d_comb_TI |
| cm_inv_var_2d_comb_TI |
| stat_rmad_TI |
| cm_inv_diff_3d_comb_TI |
| cm_sum_entr_3d_comb_TI |
| cm_joint_max_2_5d_comb_TI |
| ngl_dc_entr_3d_TI |
| ngt_busyness_2d_TI |
| morph_area_dens_aee_TI |
| cm_energy_3d_avg_TI |
| rlm_rl_var_3d_avg_TI |
| morph_area_dens_aabb_TI |
| rlm_r_perc_2_5d_avg_TI |
| cm_clust_shade_2d_avg_TI |
| szm_glnu_norm_2d_TI |
| ih_min_grad_g_TI |
| cm_inv_var_2_5d_comb_TI |
| ih_mad_TI |
| stat_energy_TI |
| cm_energy_2_5d_avg_TI |
| cm_contrast_3d_comb_TI |
| cm_inv_diff_mom_2d_avg_TI |
| cm_inv_var_2_5d_avg_TI |
| cm_clust_shade_3d_avg_TI |
| dzm_ldlge_2d_TI |
| ngl_hdlge_2_5d_TI |
| ih_mode_TI |
| morph_vol_dens_conv_hull_TI |
| ih_max_grad_g_TI |
| ngl_hdhge_2_5d_TI |
| cm_sum_var_2d_avg_TI |
| cm_clust_shade_2d_comb_TI |
| ivh_auc_TI |
| morph_vol_dens_aee_TI |
| cm_inv_var_3d_comb_TI |
| cm_diff_var_2_5d_avg_TI |
| morph_area_dens_ombb_TI |
| morph_geary_c_TI |
| ngt_coarseness_2d_TI |
| szm_gl_var_2d_TI |
| ngt_strength_2_5d_TI |
| ngt_contrast_2d_TI |
| cm_auto_corr_2d_comb_TI |
| ngl_hdlge_3d_TI |
| ih_kurt_TI |
| ih_min_grad_TI |
| ngl_lde_2_5d_TI |
| ngl_hde_2_5d_TI |
| dzm_ldlge_3d_TI |
| ngl_hdhge_2d_TI |
| cm_sum_var_2d_comb_TI |
| dzm_ldlge_2_5d_TI |
| ngt_complexity_3d_TI |
| **Tibial Cartilage Radiomic Model (n=42)**‡ |
| cm_joint_avg_2d_comb_TC |
| stat_min_TC |
| stat_cov_TC |
| cm_joint_max_3d_comb_TC |
| cm_info_corr1_2d_avg_TC |
| cm_clust_prom_2_5d_avg_TC |
| cm_info_corr1_2d_comb_TC |
| morph_area_dens_ombb_TC |
| morph_area_dens_aabb_TC |
| cm_info_corr2_3d_avg_TC |
| cm_joint_max_2_5d_avg_TC |
| ngl_ldlge_2_5d_TC |
| dzm_zdnu_norm_3d_TC |
| szm_zs_var_2_5d_TC |
| ih_p10_TC |
| szm_glnu_norm_3d_TC |
| morph_pca_elongatcon_TC |
| cm_inv_diff_norm_2d_avg_TC |
| ih_mode_TC |
| dzm_ldlge_2d_TC |
| cm_clust_shade_3d_avg_TC |
| ngt_contrast_2d_TC |
| stat_qcod_TC |
| dzm_ldlge_3d_TC |
| cm_auto_corr_2d_avg_TC |
| ngt_complexity_3d_TC |
| ngl_lde_2_5d_TC |
| loc_peak_loc_TC |
| dzm_sdlge_2d_TC |
| ih_max_grad_g_TC |
| ngl_dcnu_norm_2_5d_TC |
| ngt_busyness_2d_TC |
| stat_rmad_TC |
| morph_com_TC |
| szm_glnu_2d_TC |
| stat_energy_TC |
| ngl_ldhge_2_5d_TC |
| ih_p90_TC |
| ih_max_grad_TC |
| stat_skew_TC |
| morph_moran_i_TC |
| cm_energy_3d_comb_TC |
| **Lateral Meniscus Radiomic Model (n=59)**‡ |
| dzm_zd_var_2d_LM |
| morph_vol_dens_aee_LM |
| morph_vol_dens_conv_hull_LM |
| dzm_zdnu_norm_2d_LM |
| ngl_ldlge_3d_LM |
| ngl_ldhge_2_5d_LM |
| morph_area_dens_mvee_LM |
| dzm_ldlge_2_5d_LM |
| dzm_ldhge_2d_LM |
| ngl_lde_2_5d_LM |
| stat_energy_LM |
| stat_var_LM |
| dzm_ldhge_3d_LM |
| dzm_ldlge_3d_LM |
| morph_area_dens_aee_LM |
| dzm_sdhge_3d_LM |
| cm_clust_prom_2d_comb_LM |
| ih_min_grad_LM |
| ngt_complexity_3d_LM |
| dzm_sdhge_2d_LM |
| szm_lzlge_3d_LM |
| morph_comp_2_LM |
| szm_szlge_2_5d_LM |
| cm_info_corr1_2d_comb_LM |
| ih_max_grad_LM |
| loc_peak_glob_LM |
| stat_min_LM |
| dzm_zd_entr_2d_LM |
| cm_clust_prom_3d_comb_LM |
| dzm_zdnu_2d_LM |
| ngt_complexity_2d_LM |
| morph_pca_elongalmon_LM |
| morph_geary_c_LM |
| dzm_sdhge_2_5d_LM |
| stat_rmad_LM |
| morph_vol_dens_ombb_LM |
| morph_com_LM |
| szm_szlge_2d_LM |
| morph_diam_LM |
| ngt_contrast_2d_LM |
| rlm_rl_var_2d_comb_LM |
| ngl_hdlge_2_5d_LM |
| cm_diff_var_2d_comb_LM |
| loc_peak_loc_LM |
| ngt_strength_2d_LM |
| dzm_sdlge_3d_LM |
| ngt_busyness_3d_LM |
| cm_joint_max_3d_avg_LM |
| cm_info_corr2_2d_avg_LM |
| ih_min_grad_g_LM |
| cm_energy_2d_comb_LM |
| cm_info_corr1_2_5d_comb_LM |
| ih_mode_LM |
| morph_moran_i_LM |
| cm_joint_avg_2d_avg_LM |
| ngl_hde_2_5d_LM |
| cm_joint_max_2d_avg_LM |
| dzm_zd_var_3d_LM |
| morph_pca_maj_axis_LM |
| **Medial Meniscus Radiomic Model (n=103)**‡ |
| stat_var_MM |
| morph_vol_dens_mvee_MM |
| ngl_hdhge_2_5d_MM |
| morph_vol_dens_conv_hull_MM |
| cm_info_corr1_3d_avg_MM |
| loc_peak_glob_MM |
| morph_pca_maj_axis_MM |
| cm_clust_shade_2d_avg_MM |
| morph_pca_elongammon_MM |
| szm_lzlge_2_5d_MM |
| morph_area_dens_aee_MM |
| cm_inv_diff_mom_2d_avg_MM |
| ngt_complexity_3d_MM |
| morph_pca_flatness_MM |
| ngt_complexity_2d_MM |
| ngl_dc_var_2_5d_MM |
| cm_energy_3d_avg_MM |
| morph_comp_2_MM |
| dzm_zd_entr_2_5d_MM |
| cm_inv_var_2d_avg_MM |
| ih_cov_MM |
| morph_pca_least_axis_MM |
| cm_diff_avg_2d_comb_MM |
| ngl_hde_2_5d_MM |
| cm_info_corr1_2d_comb_MM |
| dzm_zdnu_2d_MM |
| cm_inv_var_2_5d_avg_MM |
| ngl_dcnu_2_5d_MM |
| morph_com_MM |
| cm_diff_var_3d_comb_MM |
| ngt_busyness_2_5d_MM |
| rlm_sre_2_5d_avg_MM |
| dzm_zd_var_2d_MM |
| cm_inv_diff_2d_avg_MM |
| ivh_auc_MM |
| ngt_strength_2d_MM |
| ngl_hdlge_2d_MM |
| szm_lze_2_5d_MM |
| cm_energy_2_5d_comb_MM |
| szm_szlge_2_5d_MM |
| morph_diam_MM |
| ngl_dc_var_2d_MM |
| cm_info_corr1_2d_avg_MM |
| ngl_dc_var_3d_MM |
| cm_info_corr2_2_5d_avg_MM |
| cm_diff_var_2d_avg_MM |
| dzm_zdnu_norm_3d_MM |
| morph_geary_c_MM |
| szm_zs_var_3d_MM |
| cm_inv_diff_norm_2d_comb_MM |
| ngl_ldlge_2d_MM |
| ngl_ldlge_2_5d_MM |
| stat_cov_MM |
| cm_corr_2_5d_avg_MM |
| ngl_dc_energy_3d_MM |
| dzm_zd_var_2_5d_MM |
| dzm_ldlge_3d_MM |
| szm_zs_var_2d_MM |
| cm_joint_max_2d_comb_MM |
| szm_glnu_norm_2_5d_MM |
| szm_glnu_norm_3d_MM |
| cm_joint_max_3d_comb_MM |
| cm_joint_max_2_5d_comb_MM |
| stat_p10_MM |
| ngl_lde_2_5d_MM |
| cm_diff_entr_3d_comb_MM |
| dzm_sdlge_2_5d_MM |
| cm_info_corr2_3d_avg_MM |
| ngl_ldhge_2_5d_MM |
| cm_inv_diff_mom_2_5d_avg_MM |
| ih_max_grad_MM |
| cm_sum_entr_2_5d_comb_MM |
| szm_szhge_3d_MM |
| cm_joint_avg_2d_comb_MM |
| cm_diff_entr_3d_avg_MM |
| ngl_dcnu_norm_2_5d_MM |
| morph_area_dens_mvee_MM |
| dzm_zd_var_3d_MM |
| morph_area_dens_conv_hull_MM |
| dzm_sdhge_2d_MM |
| cm_clust_shade_2d_comb_MM |
| szm_szlge_2d_MM |
| ngt_busyness_2d_MM |
| cm_joint_max_3d_avg_MM |
| ih_max_grad_g_MM |
| ngt_complexity_2_5d_MM |
| ih_min_grad_g_MM |
| ih_mode_MM |
| ngl_hdlge_2_5d_MM |
| szm_lzhge_2d_MM |
| cm_auto_corr_2_5d_avg_MM |
| cm_inv_var_2d_comb_MM |
| ngl_hdhge_3d_MM |
| loc_peak_loc_MM |
| cm_joint_max_2d_avg_MM |
| szm_zs_entr_2_5d_MM |
| cm_info_corr1_3d_comb_MM |
| cm_corr_2d_avg_MM |
| ngl_hdlge_3d_MM |
| morph_vol_dens_aabb_MM |
| ih_entropy_MM |
| cm_sum_entr_3d_comb_MM |
| ih_uniformity_MM |
| **Load-Bearing Tissue Radiomic Model (n=229)**‡ |
| stat_cov_TC |
| dzm_sdhge_2_5d_LM |
| dzm_zd_var_3d_LM |
| morph_area_dens_mvee_TI |
| dzm_ldhge_2d_LM |
| ngl_hde_2_5d_MM |
| cm_info_corr1_3d_avg_MM |
| ih_skew_FE |
| morph_pca_elongation_TI |
| stat_rmad_LM |
| dzm_ldlge_3d_LM |
| cm_energy_3d_avg_MM |
| morph_area_dens_ombb_TI |
| ngl_hde_2_5d_LM |
| morph_com_TC |
| dzm_ldhge_2_5d_FE |
| morph_pca_least_axis_TI |
| szm_glnu_2d_TC |
| stat_rmad_TC |
| dzm_sdhge_2d_FE |
| stat_range_FE |
| morph_vol_dens_conv_hull_LM |
| morph_diam_FE |
| cm_auto_corr_2_5d_comb_FE |
| cm_auto_corr_2d_comb_FE |
| morph_comp_2_MM |
| ngl_dcnu_norm_2_5d_MM |
| cm_info_corr1_2_5d_avg_FE |
| morph_area_dens_aabb_FE |
| ngl_hdhge_2_5d_MM |
| ngt_busyness_3d_TI |
| morph_vol_dens_aabb_TI |
| dzm_sdhge_2d_LM |
| morph_area_dens_aabb_TI |
| morph_area_dens_aee_FE |
| cm_joint_avg_2d_comb_TC |
| cm_clust_prom_3d_comb_FE |
| dzm_zd_var_2d_LM |
| ivh_auc_MM |
| cm_auto_corr_2d_avg_FE |
| stat_var_MM |
| stat_rmad_TI |
| cm_energy_2_5d_comb_TI |
| ngl_dcnu_norm_2_5d_TC |
| cm_info_corr2_2d_avg_LM |
| morph_sph_dispr_FC |
| ngl_ldlge_3d_LM |
| cm_clust_shade_2d_avg_MM |
| szm_szhge_2_5d_FE |
| ih_mad_TI |
| cm_auto_corr_3d_comb_TI |
| ih_uniformity_MM |
| ih_kurt_TI |
| stat_skew_TC |
| stat_qcod_FE |
| ngl_dc_var_3d_FE |
| dzm_ldlge_2d_TC |
| szm_gl_var_2d_TI |
| ngt_contrast_2_5d_TI |
| dzm_zdnu_norm_3d_TC |
| dzm_sdhge_2d_MM |
| ih_p10_TC |
| cm_info_corr2_2d_avg_FE |
| cm_corr_2_5d_avg_MM |
| cm_info_corr1_2d_comb_MM |
| cm_sum_entr_2_5d_avg_TI |
| ngl_ldhge_2_5d_FE |
| morph_com_MM |
| dzm_zdnu_norm_3d_FE |
| cm_contrast_3d_comb_TI |
| ngl_hdlge_2d_MM |
| dzm_ldhge_3d_LM |
| cm_inv_diff_2d_avg_MM |
| dzm_zdnu_2d_LM |
| dzm_sdlge_2d_TC |
| ngl_dcnu_norm_2_5d_FE |
| ih_median_TI |
| stat_min_TC |
| cm_inv_diff_mom_2d_avg_MM |
| ih_kurt_FE |
| ih_max_grad_FC |
| cm_info_corr1_2d_comb_FE |
| cm_info_corr1_2_5d_comb_LM |
| ngl_hdhge_2_5d_TI |
| dzm_zdnu_norm_2d_LM |
| cm_inv_diff_mom_2_5d_comb_FE |
| stat_cov_MM |
| dzm_lde_2_5d_FE |
| cm_clust_prom_2_5d_comb_TI |
| morph_moran_i_LM |
| stat_qcod_TC |
| szm_szlge_2_5d_LM |
| morph_geary_c_MM |
| ngt_complexity_3d_MM |
| stat_median_FE |
| cm_joint_max_2_5d_avg_FE |
| dzm_ldlge_2d_TI |
| szm_lzlge_2_5d_MM |
| ih_min_grad_LM |
| morph_area_dens_ombb_TC |
| cm_joint_max_3d_avg_LM |
| morph_area_dens_aee_LM |
| ivh_auc_FE |
| cm_diff_entr_3d_avg_MM |
| ngl_lde_2_5d_FE |
| cm_info_corr1_2d_avg_TC |
| szm_zs_entr_2_5d_FE |
| cm_joint_max_2_5d_comb_FE |
| cm_clust_shade_2d_avg_FC |
| cm_inv_diff_norm_2d_comb_MM |
| ngl_dcnu_2_5d_MM |
| cm_clust_shade_3d_comb_FE |
| stat_kurt_TI |
| morph_pca_maj_axis_MM |
| cm_inv_diff_mom_norm_3d_avg_TI |
| ngl_dc_var_2_5d_FE |
| cm_energy_3d_comb_TC |
| ngl_lde_2_5d_LM |
| morph_diam_MM |
| morph_moran_i_TC |
| rlm_r_perc_2_5d_avg_TI |
| szm_szhge_3d_FE |
| cm_diff_avg_2d_comb_MM |
| morph_area_dens_aee_MM |
| loc_peak_loc_TI |
| morph_area_dens_conv_hull_FC |
| cm_joint_avg_2d_avg_LM |
| cm_clust_prom_2d_comb_LM |
| loc_peak_loc_FC |
| dzm_zd_entr_2_5d_MM |
| rlm_rl_var_2d_comb_LM |
| ngl_ldlge_2d_MM |
| cm_diff_var_2_5d_avg_TI |
| stat_energy_LM |
| ih_cov_MM |
| ih_iqr_FC |
| cm_sum_var_2d_comb_FE |
| cm_energy_3d_avg_TI |
| szm_zs_var_2d_MM |
| ivh_auc_TI |
| cm_diff_entr_3d_comb_MM |
| szm_zsnu_2d_FE |
| morph_area_dens_mvee_MM |
| morph_pca_elongammon_MM |
| ngt_busyness_2d_TC |
| loc_peak_glob_FE |
| loc_peak_loc_FE |
| cm_inv_diff_3d_comb_TI |
| cm_info_corr2_3d_comb_FE |
| szm_zs_var_2_5d_TC |
| morph_pca_elongation_FE |
| morph_vol_dens_aabb_FE |
| cm_sum_entr_2d_comb_FE |
| morph_area_dens_mvee_FC |
| ngl_dc_var_2d_MM |
| ngt_coarseness_2d_TI |
| cm_energy_2_5d_avg_TI |
| stat_skew_FE |
| cm_clust_shade_2_5d_comb_TI |
| ih_min_grad_g_TI |
| ngl_dc_energy_2_5d_FE |
| cm_inv_diff_mom_2_5d_avg_MM |
| loc_peak_glob_TI |
| ngl_ldhge_2_5d_LM |
| cm_joint_max_3d_comb_TC |
| ngt_complexity_2d_MM |
| morph_vol_dens_conv_hull_MM |
| cm_auto_corr_2d_comb_TI |
| ngl_hde_2_5d_TI |
| cm_clust_shade_2d_comb_MM |
| morph_diam_LM |
| ih_entropy_MM |
| cm_inv_diff_norm_2d_avg_TC |
| morph_pca_least_axis_MM |
| cm_energy_2d_avg_FE |
| morph_vol_dens_aee_TI |
| cm_energy_2_5d_comb_MM |
| cm_info_corr1_2_5d_comb_FE |
| cm_joint_max_2d_comb_MM |
| cm_clust_shade_3d_avg_TC |
| morph_pca_elongalmon_LM |
| cm_joint_avg_2d_comb_MM |
| ngl_dc_entr_3d_TI |
| cm_sum_entr_3d_comb_TI |
| ngl_glnu_2d_FE |
| dzm_ldlge_2_5d_LM |
| cm_energy_2d_comb_LM |
| szm_szlge_2_5d_MM |
| cm_info_corr1_3d_comb_MM |
| cm_inv_diff_2d_avg_TI |
| cm_clust_shade_2d_comb_TI |
| rlm_rl_var_3d_avg_TI |
| ih_mode_TI |
| ngl_ldlge_2_5d_TC |
| stat_cov_FE |
| cm_info_corr2_2_5d_avg_MM |
| ngt_busyness_2_5d_MM |
| ngl_hdhge_2d_FE |
| cm_energy_2_5d_comb_FE |
| rlm_sre_2_5d_avg_MM |
| morph_pca_maj_axis_LM |
| cm_joint_max_3d_avg_FE |
| morph_comp_2_FE |
| morph_area_dens_conv_hull_MM |
| dzm_ldlge_2d_FE |
| ngt_strength_2d_MM |
| ngl_hdlge_2_5d_FE |
| cm_info_corr1_2d_comb_LM |
| cm_clust_shade_2d_avg_TI |
| cm_info_corr1_2d_avg_FE |
| morph_pca_flatness_MM |
| morph_area_dens_aabb_TC |
| ngt_strength_2d_FE |
| cm_clust_shade_3d_avg_TI |
| cm_joint_max_3d_comb_FE |
| cm_inv_var_2d_avg_MM |
| dzm_zd_var_2d_MM |
| ih_iqr_FE |
| cm_diff_var_2d_comb_LM |
| cm_clust_prom_2_5d_avg_TC |
| cm_inv_var_2_5d_avg_TI |
| morph_pca_flatness_FE |
| szm_szlge_2d_FE |
| cm_energy_2_5d_avg_FE |
| morph_vol_dens_mvee_MM |
| cm_inv_var_3d_comb_TI |
| dzm_sdhge_3d_LM |
| ih_median_FE |
| szm_zs_var_3d_MM |
| **Biochemical biomarker Model (n=17)** |
| sCOMP |
| sHA |
| sPⅡANP |
| sCTXⅠ |
| sCS846 |
| sMMP-3 |
| sC2C |
| sC1, 2C |
| sCPⅡ |
| sNTXⅠ |
| sColl2_1_NO2 |
| uCTXⅠ-α |
| uCTXⅠ-β |
| uNTXⅠ |
| uC2C |
| uC1, 2C |
| uColl2_1_NO2 |
| uCTXⅡ |
| **Clinical Model (n=7)** |
| Baseline WOMAC knee pain score |
| Baseline WOMAC knee disability score |
| Age (year) |
| Sex |
| Race |
| Baseline knee pain medication |
| Baseline BMI (kg/m^2^) |
| **Biochemical biomarker plus Clinical variable Model (n=25)** |
| Baseline WOMAC knee pain score |
| Baseline WOMAC knee disability score |
| Age (year) |
| Sex |
| Race |
| Baseline knee pain medication |
| Baseline BMI (kg/m^2^) |
| sCOMP |
| sHA |
| sPⅡANP |
| sCTXⅠ |
| sCS846 |
| sMMP-3 |
| sC2C |
| sC1, 2C |
| sCPⅡ |
| sNTXⅠ |
| sColl2_1_NO2 |
| uCTXⅠ-α |
| uCTXⅠ-β |
| uNTXⅠ |
| uC2C |
| uC1, 2C |
| uColl2_1_NO2 |
| uCTXⅡ |
| **Load-Bearing Tissue Radiomic plus Biochemical biomarker and Clinical variable Model (n=255)** ‡ |
| cm_auto_corr_2_5d_comb_FE |
| cm_auto_corr_2d_avg_FE |
| cm_auto_corr_2d_avg_TC |
| cm_auto_corr_2d_comb_FE |
| cm_auto_corr_2d_comb_TI |
| cm_auto_corr_3d_comb_TI |
| cm_clust_prom_2d_comb_FE |
| cm_clust_prom_2d_comb_LM |
| cm_clust_prom_3d_comb_FE |
| cm_clust_prom_3d_comb_LM |
| cm_clust_shade_2_5d_comb_TI |
| cm_clust_shade_2d_avg_FC |
| cm_clust_shade_2d_avg_FE |
| cm_clust_shade_2d_avg_TI |
| cm_clust_shade_2d_comb_MM |
| cm_clust_shade_2d_comb_TI |
| cm_clust_shade_3d_avg_TI |
| cm_clust_shade_3d_comb_FE |
| cm_contrast_3d_comb_TI |
| cm_corr_2_5d_avg_MM |
| cm_corr_2d_avg_MM |
| cm_diff_avg_2d_comb_MM |
| cm_diff_entr_3d_comb_MM |
| cm_diff_var_2_5d_avg_TI |
| cm_diff_var_2d_avg_FE |
| cm_diff_var_2d_comb_FE |
| cm_diff_var_2d_comb_LM |
| cm_energy_2_5d_avg_TI |
| cm_energy_2_5d_comb_FE |
| cm_energy_2_5d_comb_TI |
| cm_energy_2d_avg_FE |
| cm_energy_2d_comb_LM |
| cm_energy_2d_comb_TI |
| cm_energy_3d_avg_MM |
| cm_energy_3d_avg_TI |
| cm_energy_3d_comb_TC |
| cm_info_corr1_2_5d_avg_FE |
| cm_info_corr1_2_5d_comb_FE |
| cm_info_corr1_2d_avg_MM |
| cm_info_corr1_2d_avg_TC |
| cm_info_corr1_2d_comb_FE |
| cm_info_corr1_2d_comb_LM |
| cm_info_corr1_2d_comb_MM |
| cm_info_corr1_3d_avg_MM |
| cm_info_corr1_3d_comb_MM |
| cm_info_corr2_2_5d_avg_MM |
| cm_info_corr2_2d_avg_FE |
| cm_info_corr2_2d_avg_LM |
| cm_info_corr2_3d_comb_FE |
| cm_inv_diff_2d_avg_MM |
| cm_inv_diff_2d_avg_TI |
| cm_inv_diff_3d_comb_TI |
| cm_inv_diff_mom_2_5d_avg_MM |
| cm_inv_diff_mom_2_5d_comb_FE |
| cm_inv_diff_mom_2d_avg_TI |
| cm_inv_diff_mom_norm_3d_avg_TI |
| cm_inv_diff_norm_2d_comb_MM |
| cm_inv_var_2_5d_avg_TI |
| cm_inv_var_2d_comb_TI |
| cm_inv_var_3d_comb_TI |
| cm_joint_avg_2d_avg_LM |
| cm_joint_avg_2d_comb_MM |
| cm_joint_avg_2d_comb_TC |
| cm_joint_max_2_5d_avg_FE |
| cm_joint_max_2d_avg_MM |
| cm_joint_max_2d_comb_MM |
| cm_joint_max_3d_avg_FE |
| cm_joint_max_3d_avg_LM |
| cm_joint_max_3d_comb_TC |
| cm_sum_entr_2_5d_avg_TI |
| cm_sum_entr_2d_comb_FE |
| cm_sum_entr_3d_comb_TI |
| dzm_lde_2_5d_FE |
| dzm_ldhge_2_5d_FE |
| dzm_ldhge_2d_FE |
| dzm_ldhge_2d_LM |
| dzm_ldhge_3d_FE |
| dzm_ldlge_2d_FE |
| dzm_ldlge_2d_TC |
| dzm_ldlge_2d_TI |
| dzm_ldlge_3d_LM |
| dzm_sde_2d_FE |
| dzm_sdhge_2_5d_LM |
| dzm_sdhge_2d_FE |
| dzm_sdhge_2d_LM |
| dzm_sdhge_2d_MM |
| dzm_sdhge_3d_LM |
| dzm_sdlge_2d_TC |
| dzm_zd_entr_2_5d_MM |
| dzm_zd_var_2_5d_MM |
| dzm_zd_var_2d_LM |
| dzm_zd_var_3d_LM |
| dzm_zd_var_3d_MM |
| dzm_zdnu_2d_LM |
| dzm_zdnu_2d_MM |
| dzm_zdnu_norm_2d_LM |
| dzm_zdnu_norm_3d_FE |
| dzm_zdnu_norm_3d_TC |
| ih_entropy_MM |
| ih_iqr_FC |
| ih_iqr_FE |
| ih_kurt_FE |
| ih_kurt_TI |
| ih_mad_TI |
| ih_median_TI |
| ih_min_grad_g_TI |
| ih_min_grad_LM |
| ih_mode_MM |
| ih_mode_TI |
| ih_p10_TC |
| ih_p90_TC |
| ih_rmad_FE |
| ih_skew_FE |
| ih_uniformity_MM |
| ivh_auc_FE |
| ivh_auc_MM |
| ivh_auc_TI |
| loc_peak_glob_FE |
| loc_peak_glob_MM |
| loc_peak_glob_TI |
| loc_peak_loc_FC |
| loc_peak_loc_TC |
| loc_peak_loc_TI |
| morph_area_dens_aabb_FE |
| morph_area_dens_aabb_TC |
| morph_area_dens_aabb_TI |
| morph_area_dens_aee_FE |
| morph_area_dens_aee_LM |
| morph_area_dens_aee_MM |
| morph_area_dens_conv_hull_FE |
| morph_area_dens_conv_hull_MM |
| morph_area_dens_mvee_FE |
| morph_area_dens_mvee_MM |
| morph_area_dens_mvee_TI |
| morph_area_dens_ombb_TI |
| morph_com_MM |
| morph_com_TC |
| morph_comp_2_FE |
| morph_comp_2_MM |
| morph_diam_FE |
| morph_diam_LM |
| morph_geary_c_MM |
| morph_moran_i_FE |
| morph_moran_i_LM |
| morph_moran_i_TC |
| morph_pca_elongalmon_LM |
| morph_pca_elongatcon_TC |
| morph_pca_elongation_FE |
| morph_pca_elongation_TI |
| morph_pca_flatness_FE |
| morph_pca_flatness_MM |
| morph_pca_least_axis_MM |
| morph_pca_least_axis_TI |
| morph_pca_maj_axis_MM |
| morph_sph_dispr_FC |
| morph_vol_dens_aabb_MM |
| morph_vol_dens_aabb_TI |
| morph_vol_dens_aee_TI |
| morph_vol_dens_conv_hull_LM |
| morph_vol_dens_mvee_FE |
| morph_vol_dens_mvee_MM |
| ngl_dc_energy_2_5d_FE |
| ngl_dc_energy_2d_FE |
| ngl_dc_entr_3d_TI |
| ngl_dc_var_2_5d_FE |
| ngl_dc_var_2_5d_MM |
| ngl_dc_var_2d_MM |
| ngl_dc_var_3d_FE |
| ngl_dcnu_2d_FE |
| ngl_dcnu_norm_2_5d_FE |
| ngl_dcnu_norm_2_5d_MM |
| ngl_dcnu_norm_2_5d_TC |
| ngl_hde_2_5d_FE |
| ngl_hde_2_5d_LM |
| ngl_hde_2_5d_MM |
| ngl_hdhge_2_5d_MM |
| ngl_hdhge_2_5d_TI |
| ngl_hdhge_3d_MM |
| ngl_hdlge_2_5d_FE |
| ngl_hdlge_2_5d_MM |
| ngl_hdlge_2_5d_TI |
| ngl_hdlge_2d_MM |
| ngl_lde_2_5d_FE |
| ngl_lde_2_5d_LM |
| ngl_lde_2_5d_MM |
| ngl_ldhge_2_5d_FE |
| ngl_ldhge_2_5d_MM |
| ngl_ldlge_2_5d_FE |
| ngl_ldlge_3d_LM |
| ngt_busyness_2_5d_MM |
| ngt_busyness_2d_TC |
| ngt_busyness_3d_LM |
| ngt_busyness_3d_TI |
| ngt_coarseness_2d_TI |
| ngt_complexity_2_5d_FE |
| ngt_complexity_2_5d_MM |
| ngt_complexity_2d_FE |
| ngt_complexity_2d_LM |
| ngt_complexity_2d_MM |
| ngt_complexity_3d_MM |
| ngt_complexity_3d_TC |
| ngt_contrast_2_5d_FE |
| ngt_contrast_2_5d_TI |
| ngt_strength_2_5d_TI |
| ngt_strength_2d_FE |
| Baseline knee pain medication |
| Baseline BMI (kg/m^2^) |
| Race |
| rlm_rl_var_2d_comb_FE |
| rlm_rl_var_3d_avg_TI |
| stat_cov_FE |
| stat_cov_MM |
| stat_cov_TC |
| stat_energy_LM |
| stat_iqr_TI |
| stat_kurt_TI |
| stat_median_FE |
| stat_min_TC |
| stat_qcod_TC |
| stat_range_FE |
| stat_rmad_LM |
| stat_rmad_TC |
| stat_skew_TC |
| stat_var_MM |
| szm_gl_var_2d_TI |
| szm_glnu_2d_TC |
| szm_glnu_norm_3d_MM |
| szm_glnu_norm_3d_TC |
| szm_lzlge_2_5d_MM |
| szm_szhge_3d_FE |
| szm_szlge_2_5d_LM |
| szm_szlge_2_5d_MM |
| szm_szlge_2d_FE |
| szm_szlge_2d_LM |
| szm_szlge_2d_MM |
| szm_zs_entr_2_5d_FE |
| szm_zs_var_2_5d_TC |
| szm_zs_var_2d_MM |
| szm_zsnu_2d_FE |
| Age (year) |
| sC1, 2C |
| sC2C |
| sColl2_1_NO2 |
| sCOMP |
| sCPⅡ |
| sCS846 |
| sCTXⅠ |
| sHA |
| sNTXⅠ |
| sPⅡANP |
| uCTXⅠ-α |
| uC2C |
| uCTXⅡ |
| Baseline WOMAC knee pain score |
| Baseline WOMAC knee disability score |
| **Femur MOAKS Model (n=18)** |
| Bone marrow lesion (% lesion that is edema): femur lateral anterior (trochlear) |
| Bone marrow lesion (% lesion that is edema): femur medial anterior (trochlear) |
| Number of bone marrow lesions: femur lateral anterior (trochlear) |
| Number of bone marrow lesions: femur medial anterior (trochlear) |
| Bone marrow lesion size: femur lateral anterior (trochlear) |
| Bone marrow lesion size: femur medial anterior (trochlear) |
| Bone marrow lesion (% lesion that is edema): femur lateral central |
| Bone marrow lesion (% lesion that is edema): femur medial central |
| Number of bone marrow lesions: femur lateral central |
| Number of bone marrow lesions: femur medial central |
| Bone marrow lesion size: femur lateral central |
| Bone marrow lesion size: femur medial central |
| Bone marrow lesion (% lesion that is edema): femur lateral posterior |
| Bone marrow lesion (% lesion that is edema): femur medial posterior |
| Number of bone marrow lesions: femur lateral posterior |
| Number of bone marrow lesions: femur medial posterior |
| Bone marrow lesion size: femur lateral posterior |
| Bone marrow lesion size: femur medial posterior |
| **Femoral Cartilage MOAKS Model (n=6)** |
| Cartilage Morphology: femur lateral anterior (trochlear) |
| Cartilage Morphology: femur medial anterior (trochlear) |
| Cartilage Morphology: femur lateral central |
| Cartilage Morphology: femur medial central |
| Cartilage Morphology: femur lateral posterior |
| Cartilage Morphology: femur medial posterior |
| **Tibia MOAKS Model (n=21)** |
| Bone marrow lesion (% lesion that is edema): tibia lateral anterior |
| Bone marrow lesion (% lesion that is edema): tibia medial anterior |
| Number of bone marrow lesions: tibia lateral anterior |
| Number of bone marrow lesions: tibia medial anterior |
| Bone marrow lesion size: tibia lateral anterior |
| Bone marrow lesion size: tibia medial anterior |
| Bone marrow lesion (% lesion that is edema): tibia lateral central |
| Bone marrow lesion (% lesion that is edema): tibia medial central |
| Number of bone marrow lesions: tibia lateral central |
| Number of bone marrow lesions: tibia medial central |
| Bone marrow lesion size: tibia lateral central |
| Bone marrow lesion size: tibia medial central |
| Bone marrow lesion (% lesion that is edema): tibia lateral posterior |
| Bone marrow lesion (% lesion that is edema): tibia medial posterior |
| Number of bone marrow lesions: tibia lateral posterior |
| Number of bone marrow lesions: tibia medial posterior |
| Bone marrow lesion size: tibia lateral posterior |
| Bone marrow lesion size: tibia medial posterior |
| Bone marrow lesion (% lesion that is edema): tibia sub-spinous |
| Number of bone marrow lesions: tibia sub-spinous |
| Bone marrow lesion size: tibia sub-spinous |
| **Tibial Cartilage MOAKS Model (n=6)** |
| Cartilage Morphology: tibia lateral anterior |
| Cartilage Morphology: tibia medial anterior |
| Cartilage Morphology: tibia lateral central |
| Cartilage Morphology: tibia medial central |
| Cartilage Morphology: tibia lateral posterior |
| Cartilage Morphology: tibia medial posterior |
| **Lateral Meniscus MOAKS Model (n=13)** |
| Lateral Meniscal Extrusion: anteriorly |
| Lateral Meniscal Extrusion: laterally |
| Lateral Meniscal Hypertrophy: anterior horn |
| Lateral Meniscal Hypertrophy: body |
| Lateral Meniscal Hypertrophy: posterior horn |
| Lateral Meniscal Signal Abnormality: anterior horn |
| Lateral Meniscal Signal Abnormality: body |
| Lateral Meniscal Signal Abnormality: posterior horn |
| Lateral Meniscal Morphology: anterior horn |
| Lateral Meniscal Morphology: body |
| Lateral Meniscal Morphology: posterior horn |
| Lateral Meniscal Morphology: anterior root tear |
| Lateral Meniscal Morphology: posterior root tear |
| **Medial Meniscus MOAKS Model (n=13)** |
| Medial Meniscal Extrusion: anteriorly |
| Medial Meniscal Extrusion: laterally |
| Medial Meniscal Hypertrophy: anterior horn |
| Medial Meniscal Hypertrophy: body |
| Medial Meniscal Hypertrophy: posterior horn |
| Medial Meniscal Signal Abnormality: anterior horn |
| Medial Meniscal Signal Abnormality: body |
| Medial Meniscal Signal Abnormality: posterior horn |
| Medial Meniscal Morphology: anterior horn |
| Medial Meniscal Morphology: body |
| Medial Meniscal Morphology: posterior horn |
| Medial Meniscal Morphology: anterior root tear |
| Medial Meniscal Morphology: posterior root tear |
| **Load-Bearing Tissue MOAKS Model (n=77)** |
| Bone marrow lesion (% lesion that is edema): femur lateral anterior (trochlear) |
| Bone marrow lesion (% lesion that is edema): femur medial anterior (trochlear) |
| Number of bone marrow lesions: femur lateral anterior (trochlear) |
| Number of bone marrow lesions: femur medial anterior (trochlear) |
| Bone marrow lesion size: femur lateral anterior (trochlear) |
| Bone marrow lesion size: femur medial anterior (trochlear) |
| Bone marrow lesion (% lesion that is edema): femur lateral central |
| Bone marrow lesion (% lesion that is edema): femur medial central |
| Number of bone marrow lesions: femur lateral central |
| Number of bone marrow lesions: femur medial central |
| Bone marrow lesion size: femur lateral central |
| Bone marrow lesion size: femur medial central |
| Bone marrow lesion (% lesion that is edema): femur lateral posterior |
| Bone marrow lesion (% lesion that is edema): femur medial posterior |
| Number of bone marrow lesions: femur lateral posterior |
| Number of bone marrow lesions: femur medial posterior |
| Bone marrow lesion size: femur lateral posterior |
| Bone marrow lesion size: femur medial posterior |
| Cartilage Morphology: femur lateral anterior (trochlear) |
| Cartilage Morphology: femur medial anterior (trochlear) |
| Cartilage Morphology: femur lateral central |
| Cartilage Morphology: femur medial central |
| Cartilage Morphology: femur lateral posterior |
| Cartilage Morphology: femur medial posterior |
| Bone marrow lesion (% lesion that is edema): tibia lateral anterior |
| Bone marrow lesion (% lesion that is edema): tibia medial anterior |
| Number of bone marrow lesions: tibia lateral anterior |
| Number of bone marrow lesions: tibia medial anterior |
| Bone marrow lesion size: tibia lateral anterior |
| Bone marrow lesion size: tibia medial anterior |
| Bone marrow lesion (% lesion that is edema): tibia lateral central |
| Bone marrow lesion (% lesion that is edema): tibia medial central |
| Number of bone marrow lesions: tibia lateral central |
| Number of bone marrow lesions: tibia medial central |
| Bone marrow lesion size: tibia lateral central |
| Bone marrow lesion size: tibia medial central |
| Bone marrow lesion (% lesion that is edema): tibia lateral posterior |
| Bone marrow lesion (% lesion that is edema): tibia medial posterior |
| Number of bone marrow lesions: tibia lateral posterior |
| Number of bone marrow lesions: tibia medial posterior |
| Bone marrow lesion size: tibia lateral posterior |
| Bone marrow lesion size: tibia medial posterior |
| Bone marrow lesion (% lesion that is edema): tibia sub-spinous |
| Number of bone marrow lesions: tibia sub-spinous |
| Bone marrow lesion size: tibia sub-spinous |
| Cartilage Morphology: tibia lateral anterior |
| Cartilage Morphology: tibia medial anterior |
| Cartilage Morphology: tibia lateral central |
| Cartilage Morphology: tibia medial central |
| Cartilage Morphology: tibia lateral posterior |
| Cartilage Morphology: tibia medial posterior |
| Lateral Meniscal Extrusion: anteriorly |
| Lateral Meniscal Extrusion: laterally |
| Lateral Meniscal Hypertrophy: anterior horn |
| Lateral Meniscal Hypertrophy: body |
| Lateral Meniscal Hypertrophy: posterior horn |
| Lateral Meniscal Signal Abnormality: anterior horn |
| Lateral Meniscal Signal Abnormality: body |
| Lateral Meniscal Signal Abnormality: posterior horn |
| Lateral Meniscal Morphology: anterior horn |
| Lateral Meniscal Morphology: body |
| Lateral Meniscal Morphology: posterior horn |
| Lateral Meniscal Morphology: anterior root tear |
| Lateral Meniscal Morphology: posterior root tear |
| Medial Meniscal Extrusion: anteriorly |
| Medial Meniscal Extrusion: laterally |
| Medial Meniscal Hypertrophy: anterior horn |
| Medial Meniscal Hypertrophy: body |
| Medial Meniscal Hypertrophy: posterior horn |
| Medial Meniscal Signal Abnormality: anterior horn |
| Medial Meniscal Signal Abnormality: body |
| Medial Meniscal Signal Abnormality: posterior horn |
| Medial Meniscal Morphology: anterior horn |
| Medial Meniscal Morphology: body |
| Medial Meniscal Morphology: posterior horn |
| Medial Meniscal Morphology: anterior root tear |
| Medial Meniscal Morphology: posterior root tear |
| **Load-Bearing Tissue MOAKS plus Biochemical biomarker and Clinical variable Model (n=102)** |
| Baseline WOMAC knee pain score |
| Baseline WOMAC knee disability score |
| Age (year) |
| Sex |
| Race |
| Baseline knee pain medication |
| Baseline BMI (kg/m^2^) |
| sCOMP |
| sHA |
| sPⅡANP |
| sCTXⅠ |
| sCS846 |
| sMMP-3 |
| sC2C |
| sC1, 2C |
| sCPⅡ |
| sNTXⅠ |
| sColl2_1_NO2 |
| uCTXⅠ-α |
| uCTXⅠ-β |
| uNTXⅠ |
| uC2C |
| uC1, 2C |
| uColl2_1_NO2 |
| uCTXⅡ |
| Bone marrow lesion (% lesion that is edema): femur lateral anterior (trochlear) |
| Bone marrow lesion (% lesion that is edema): femur medial anterior (trochlear) |
| Number of bone marrow lesions: femur lateral anterior (trochlear) |
| Number of bone marrow lesions: femur medial anterior (trochlear) |
| Bone marrow lesion size: femur lateral anterior (trochlear) |
| Bone marrow lesion size: femur medial anterior (trochlear) |
| Bone marrow lesion (% lesion that is edema): femur lateral central |
| Bone marrow lesion (% lesion that is edema): femur medial central |
| Number of bone marrow lesions: femur lateral central |
| Number of bone marrow lesions: femur medial central |
| Bone marrow lesion size: femur lateral central |
| Bone marrow lesion size: femur medial central |
| Bone marrow lesion (% lesion that is edema): femur lateral posterior |
| Bone marrow lesion (% lesion that is edema): femur medial posterior |
| Number of bone marrow lesions: femur lateral posterior |
| Number of bone marrow lesions: femur medial posterior |
| Bone marrow lesion size: femur lateral posterior |
| Bone marrow lesion size: femur medial posterior |
| Cartilage Morphology: femur lateral anterior (trochlear) |
| Cartilage Morphology: femur medial anterior (trochlear) |
| Cartilage Morphology: femur lateral central |
| Cartilage Morphology: femur medial central |
| Cartilage Morphology: femur lateral posterior |
| Cartilage Morphology: femur medial posterior |
| Bone marrow lesion (% lesion that is edema): tibia lateral anterior |
| Bone marrow lesion (% lesion that is edema): tibia medial anterior |
| Number of bone marrow lesions: tibia lateral anterior |
| Number of bone marrow lesions: tibia medial anterior |
| Bone marrow lesion size: tibia lateral anterior |
| Bone marrow lesion size: tibia medial anterior |
| Bone marrow lesion (% lesion that is edema): tibia lateral central |
| Bone marrow lesion (% lesion that is edema): tibia medial central |
| Number of bone marrow lesions: tibia lateral central |
| Number of bone marrow lesions: tibia medial central |
| Bone marrow lesion size: tibia lateral central |
| Bone marrow lesion size: tibia medial central |
| Bone marrow lesion (% lesion that is edema): tibia lateral posterior |
| Bone marrow lesion (% lesion that is edema): tibia medial posterior |
| Number of bone marrow lesions: tibia lateral posterior |
| Number of bone marrow lesions: tibia medial posterior |
| Bone marrow lesion size: tibia lateral posterior |
| Bone marrow lesion size: tibia medial posterior |
| Bone marrow lesion (% lesion that is edema): tibia sub-spinous |
| Number of bone marrow lesions: tibia sub-spinous |
| Bone marrow lesion size: tibia sub-spinous |
| Cartilage Morphology: tibia lateral anterior |
| Cartilage Morphology: tibia medial anterior |
| Cartilage Morphology: tibia lateral central |
| Cartilage Morphology: tibia medial central |
| Cartilage Morphology: tibia lateral posterior |
| Cartilage Morphology: tibia medial posterior |
| Lateral Meniscal Extrusion: anteriorly |
| Lateral Meniscal Extrusion: laterally |
| Lateral Meniscal Hypertrophy: anterior horn |
| Lateral Meniscal Hypertrophy: body |
| Lateral Meniscal Hypertrophy: posterior horn |
| Lateral Meniscal Signal Abnormality: anterior horn |
| Lateral Meniscal Signal Abnormality: body |
| Lateral Meniscal Signal Abnormality: posterior horn |
| Lateral Meniscal Morphology: anterior horn |
| Lateral Meniscal Morphology: body |
| Lateral Meniscal Morphology: posterior horn |
| Lateral Meniscal Morphology: anterior root tear |
| Lateral Meniscal Morphology: posterior root tear |
| Medial Meniscal Extrusion: anteriorly |
| Medial Meniscal Extrusion: laterally |
| Medial Meniscal Hypertrophy: anterior horn |
| Medial Meniscal Hypertrophy: body |
| Medial Meniscal Hypertrophy: posterior horn |
| Medial Meniscal Signal Abnormality: anterior horn |
| Medial Meniscal Signal Abnormality: body |
| Medial Meniscal Signal Abnormality: posterior horn |
| Medial Meniscal Morphology: anterior horn |
| Medial Meniscal Morphology: body |
| Medial Meniscal Morphology: posterior horn |
| Medial Meniscal Morphology: anterior root tear |
| Medial Meniscal Morphology: posterior root tear |

‡ Selected radiomics feature name = Radiomics feature name + anatomical structure.

FE: Femur, FC: Femoral Cartilage, TI: Tibia, TC: Tibial Cartilage, LM: Lateral Meniscus, MM: Medial Meniscus, sCOMP: serum Cartilage Oligomeric Matrix Protein (ng/mL), sHA: serum Hyaluronic Acid (ng/mL), sPⅡANP: serum type IIA Procollagen Amino terminal Propeptide (ng/mL), sCTXⅠ: serum type I collagen C-terminal Telopeptide (ng/mL), sCS846: serum aggrecan Chondroitin Sulfate 846 epitope (ng/mL), sMMP-3: serum Matrix MetalloProteinase-3 (ng/mL), sC2C: serum Cleavage neoepitope of type II Collagen (ng/mL), sC1, 2C: serum type II Collagen neoepitope (ng/mL), sCPⅡ: serum C-Propeptide of type II collagen (pg/mL), sNTXⅠ: serum N-terminal Telopeptide of type I collagen (nmol BCE), sColl2_1_NO2: serum triple helix of type II Collagen (nM), uCTXⅠ-α: urine C-terminal cross-linked Telopeptide of type I collagen-α (ng/mL), uCTXⅠ-β: urine urine C-terminal cross-linked Telopeptide of type I collagen-β (ug/L), uNTXⅠ: urine N-terminal cross-linked Telopeptide of type I collagen (nM BCE), uC2C: urine Cleavage neoepitope of type II Collagen (pg/mL), uC1, 2C: urine type II Collagen neoepitope (ug/mL), uColl2_1_NO2: urine triple helix of type II Collagen (nM), uCTXⅡ: urine C-telopeptide fragment of type II collagen (ug/L), WOMAC: Western Ontario and McMaster Universities Arthritis Index, MOAKS: Magnetic resonance imaging OsteoArthritis Knee Score.
