# Supplementary material for: Predicting knee osteoarthritis progression using neural network with longitudinal MRI radiomics, and biochemical biomarkers: A modeling study
Source: PLoS Med. 2025 Aug 21;22(8):e1004665. doi: 10.1371/journal.pmed.1004665 (PMC12370028; doi:10.1371/journal.pmed.1004665)
Supplement: S6 Table — The areas under ROC curves of predictive models in the test cohorts. (DOCX) [file pmed.1004665.s022.docx]

**Table S6. The areas under ROC curves of predictive models in the test cohorts.í**

| **Predicting models** | **Test cohort 1** | **Test cohort 2** | **Test cohort 3** | **Total test cohort** |
| --- | --- | --- | --- | --- |
| **JSN and pain progression** |  |  |  |  |
| FE-RM | 0.636 (0.569, 0.698) | 0.668 (0.600, 0.729) | 0.719 (0.650, 0.780) | 0.674 (0.635, 0.710) |
| FE-MOM | 0.624 (0.566, 0.680) | 0.627 (0.566, 0.685) | 0.608 (0.546, 0.668) | 0.621 (0.586, 0.654) |
| FC-RM | 0.530 (0.460, 0.599) | 0.552 (0.483, 0.619) | 0.552 (0.480, 0.622) | 0.543 (0.503, 0.583) |
| FC-MOM | 0.597 (0.533, 0.659) | 0.583 (0.518, 0.647) | 0.588 (0.519, 0.653) | 0.590 (0.553, 0.627) |
| TI-RM | 0.646 (0.578, 0.708) | 0.724 (0.659, 0.780) | 0.669 (0.601, 0.730) | 0.679 (0.642, 0.715) |
| TI-MOM | 0.603 (0.544, 0.659) | 0.606 (0.545, 0.663) | 0.618 (0.556, 0.677) | 0.609 (0.574, 0.642) |
| TC-RM | 0.675 (0.610, 0.734) | 0.679 (0.612, 0.740) | 0.688 (0.618, 0.751) | 0.682 (0.644, 0.717) |
| TC-MOM | 0.602 (0.542, 0.659) | 0.560 (0.494, 0.624) | 0.586 (0.521, 0.649) | 0.583 (0.547, 0.618) |
| LM-RM | 0.665 (0.599, 0.725) | 0.709 (0.643, 0.767) | 0.679 (0.606, 0.745) | 0.685 (0.646, 0.721) |
| LM-MOM | 0.520 (0.467, 0.573) | 0.566 (0.510, 0.621) | 0.549 (0.489, 0.608) | 0.545 (0.513, 0.577) |
| MM-RM | 0.656 (0.587, 0.719) | 0.729 (0.661, 0.787) | 0.653 (0.582, 0.718) | 0.680 (0.641, 0.717) |
| MM-MOM | 0.581 (0.520, 0.639) | 0.564 (0.502, 0.624) | 0.533 (0.469, 0.596) | 0.560 (0.524, 0.595) |
| LBT-RM | 0.791 (0.733, 0.840) | 0.833 (0.779, 0.876) | 0.795 (0.733, 0.846) | 0.808 (0.776, 0.836) |
| LBT-MOM | 0.730 (0.667, 0.785) | 0.734 (0.668, 0.791) | 0.736 (0.668, 0.794) | 0.734 (0.697, 0.767) |
| BM | 0.777 (0.717, 0.827) | 0.733 (0.668, 0.790) | 0.764 (0.698, 0.818) | 0.758 (0.722, 0.790) |
| Clinical model | 0.666 (0.601, 0.725) | 0.653 (0.587, 0.714) | 0.651 (0.581, 0.716) | 0.657 (0.619, 0.693) |
| BCM | 0.800 (0.739, 0.849) | 0.759 (0.695, 0.812) | 0.779 (0.713, 0.834) | 0.780 (0.745, 0.811) |
| LBTRBC-M | 0.869 (0.819, 0.907) | 0.896 (0.847, 0.930) | 0.879 (0.830, 0.915) | 0.880 (0.853, 0.903) |
| LBTMBC-M | 0.802 (0.743, 0.850) | 0.777 (0.715, 0.829) | 0.770 (0.704, 0.825) | 0.784 (0.750, 0.815) |
| LBTRB-M | 0.838 (0.785, 0.880) | 0.872 (0.823, 0.909) | 0.869 (0.820, 0.907) | 0.860 (0.832, 0.883) |
| LBTRC-M | 0.827 (0.772, 0.871) | 0.862 (0.811, 0.902) | 0.804 (0.744, 0.853) | 0.832 (0.801, 0.859) |
| **JSN progression** |  |  |  |  |
| FE-RM | 0.625 (0.533, 0.709) | 0.644 (0.546, 0.732) | 0.740 (0.663, 0.805) | 0.672 (0.621, 0.719) |
| FE-MOM | 0.630 (0.550, 0.704) | 0.604 (0.520, 0.682) | 0.577 (0.497, 0.654) | 0.603 (0.556, 0.648) |
| FC-RM | 0.520 (0.438, 0.602) | 0.573 (0.484, 0.658) | 0.561 (0.469, 0.649) | 0.555 (0.505, 0.604) |
| FC-MOM | 0.589 (0.508, 0.665) | 0.542 (0.459, 0.623) | 0.466 (0.386, 0.548) | 0.533 (0.485, 0.580) |
| TI-RM | 0.691 (0.606, 0.764) | 0.711 (0.623, 0.786) | 0.609 (0.521, 0.690) | 0.670 (0.621, 0.716) |
| TI-MOM | 0.583 (0.505, 0.657) | 0.532 (0.456, 0.607) | 0.539 (0.463, 0.613) | 0.551 (0.507, 0.594) |
| TC-RM | 0.611 (0.531, 0.685) | 0.688 (0.605, 0.761) | 0.706 (0.622, 0.778) | 0.668 (0.622, 0.712) |
| TC-MOM | 0.553 (0.482, 0.622) | 0.495 (0.416, 0.574) | 0.512 (0.434, 0.590) | 0.520 (0.476, 0.564) |
| LM-RM | 0.698 (0.616, 0.770) | 0.663 (0.580, 0.738) | 0.643 (0.555, 0.722) | 0.667 (0.620, 0.712) |
| LM-MOM | 0.537 (0.467, 0.606) | 0.562 (0.490, 0.632) | 0.532 (0.461, 0.602) | 0.545 (0.504, 0.585) |
| MM-RM | 0.707 (0.626, 0.777) | 0.736 (0.656, 0.803) | 0.726 (0.644, 0.796) | 0.723 (0.678, 0.764) |
| MM-MOM | 0.658 (0.592, 0.719) | 0.610 (0.535, 0.680) | 0.669 (0.602, 0.730) | 0.646 (0.606, 0.684) |
| LBT-RM | 0.799 (0.724, 0.857) | 0.857 (0.796, 0.903) | 0.809 (0.732, 0.868) | 0.821 (0.782, 0.855) |
| LBT-MOM | 0.677 (0.591, 0.752) | 0.681 (0.590, 0.759) | 0.661 (0.572, 0.740) | 0.674 (0.624, 0.720) |
| BM | 0.772 (0.687, 0.840) | 0.767 (0.689, 0.830) | 0.741 (0.651, 0.815) | 0.760 (0.713, 0.801) |
| Clinical model | 0.726 (0.646, 0.794) | 0.682 (0.594, 0.758) | 0.728 (0.645, 0.797) | 0.712 (0.665, 0.754) |
| BCM | 0.822 (0.751, 0.877) | 0.786 (0.705, 0.850) | 0.785 (0.703, 0.849) | 0.798 (0.755, 0.835) |
| LBTRBC-M | 0.919 (0.861, 0.954) | 0.906 (0.843, 0.946) | 0.917 (0.855, 0.954) | 0.913 (0.881, 0.937) |
| LBTMBC-M | 0.808 (0.732, 0.867) | 0.755 (0.667, 0.825) | 0.768 (0.683, 0.835) | 0.777 (0.731, 0.817) |
| LBTRB-M | 0.838 (0.769, 0.890) | 0.891 (0.829, 0.933) | 0.869 (0.799, 0.917) | 0.867 (0.831, 0.896) |
| LBTRC-M | 0.855 (0.787, 0.904) | 0.864 (0.801, 0.910) | 0.858 (0.787, 0.908) | 0.859 (0.823, 0.889) |
| **Pain progression** |  |  |  |  |
| FE,-RM | 0.689 (0.602, 0.764) | 0.656 (0.571, 0.732) | 0.587 (0.496, 0.673) | 0.642 (0.592, 0.690) |
| FE-MOM | 0.532 (0.446, 0.617) | 0.561 (0.471, 0.648) | 0.513 (0.429, 0.596) | 0.533 (0.483, 0.583) |
| FC-RM | 0.549 (0.458, 0.637) | 0.548 (0.459, 0.634) | 0.507 (0.424, 0.589) | 0.532 (0.482, 0.582) |
| FC-MOM | 0.610 (0.536, 0.679) | 0.574 (0.494, 0.651) | 0.641 (0.569, 0.708) | 0.610 (0.566, 0.651) |
| TI-RM | 0.702 (0.618, 0.774) | 0.678 (0.585, 0.759) | 0.611 (0.532, 0.684) | 0.659 (0.611, 0.705) |
| TI-MOM | 0.542 (0.478, 0.604) | 0.551 (0.478, 0.622) | 0.552 (0.484, 0.618) | 0.548 (0.509, 0.586) |
| TC-RM | 0.682 (0.602, 0.753) | 0.632 (0.538, 0.717) | 0.699 (0.614, 0.772) | 0.671 (0.622, 0.716) |
| TC-MOM | 0.557 (0.477, 0.633) | 0.513 (0.431, 0.595) | 0.563 (0.484, 0.638) | 0.545 (0.499, 0.590) |
| LM-RM | 0.682 (0.597, 0.756) | 0.681 (0.592, 0.758) | 0.701 (0.622, 0.769) | 0.687 (0.640, 0.731) |
| LM-MOM | 0.464 (0.400, 0.529) | 0.498 (0.414, 0.582) | 0.562 (0.485, 0.635) | 0.509 (0.465, 0.552) |
| MM-RM | 0.636 (0.553, 0.713) | 0.628 (0.530, 0.715) | 0.674 (0.588, 0.751) | 0.648 (0.598, 0.696) |
| MM-MOM | 0.620 (0.543, 0.691) | 0.589 (0.505, 0.668) | 0.613 (0.535, 0.686) | 0.607 (0.562, 0.650) |
| LBT-RM | 0.798 (0.719, 0.860) | 0.820 (0.750, 0.873) | 0.761 (0.685, 0.823) | 0.790 (0.748, 0.827) |
| LBT-MOM | 0.679 (0.588, 0.758) | 0.643 (0.541, 0.733) | 0.689 (0.603, 0.765) | 0.671 (0.619, 0.719) |
| BM | 0.758 (0.675, 0.826) | 0.694 (0.597, 0.776) | 0.756 (0.669, 0.825) | 0.737 (0.688, 0.781) |
| Clinical model | 0.741 (0.669, 0.802) | 0.631 (0.543, 0.712) | 0.690 (0.611, 0.760) | 0.691 (0.646, 0.732) |
| BCM | 0.801 (0.722, 0.862) | 0.706 (0.613, 0.784) | 0.765 (0.681, 0.832) | 0.760 (0.712, 0.801) |
| LBTRBC-M | 0.886 (0.834, 0.924) | 0.892 (0.833, 0.933) | 0.881 (0.824, 0.921) | 0.886 (0.856, 0.910) |
| LBTMBC-M | 0.753 (0.673, 0.819) | 0.681 (0.586, 0.763) | 0.738 (0.656, 0.805) | 0.726 (0.679, 0.769) |
| LBTRB-M | 0.858 (0.795, 0.904) | 0.843 (0.772, 0.895) | 0.837 (0.773, 0.885) | 0.845 (0.809, 0.875) |
| LBTRC-M | 0.814 (0.739, 0.871) | 0.836 (0.765, 0.889) | 0.750 (0.674, 0.813) | 0.797 (0.755, 0.833) |
| **Non progression** |  |  |  |  |
| FE-RM | 0.657 (0.587, 0.721) | 0.741 (0.680, 0.795) | 0.716 (0.647, 0.776) | 0.705 (0.668, 0.741) |
| FE-MOM | 0.634 (0.569, 0.695) | 0.622 (0.557, 0.682) | 0.609 (0.539, 0.674) | 0.622 (0.584, 0.658) |
| FC-RM | 0.543 (0.473, 0.611) | 0.545 (0.475, 0.613) | 0.544 (0.473, 0.613) | 0.543 (0.503, 0.583) |
| FC-MOM | 0.611 (0.543, 0.674) | 0.566 (0.501, 0.629) | 0.551 (0.482, 0.618) | 0.577 (0.539, 0.615) |
| TI-RM | 0.680 (0.612, 0.741) | 0.695 (0.627, 0.756) | 0.665 (0.592, 0.731) | 0.679 (0.640, 0.716) |
| TI-MOM | 0.634 (0.581, 0.683) | 0.609 (0.555, 0.661) | 0.615 (0.559, 0.668) | 0.619 (0.588, 0.649) |
| TC-RM | 0.672 (0.604, 0.734) | 0.673 (0.604, 0.735) | 0.710 (0.643, 0.769) | 0.684 (0.645, 0.720) |
| TC-MOM | 0.507 (0.447, 0.567) | 0.562 (0.501, 0.620) | 0.546 (0.483, 0.608) | 0.538 (0.503, 0.573) |
| LM-RM | 0.649 (0.577, 0.715) | 0.680 (0.612, 0.741) | 0.644 (0.570, 0.711) | 0.658 (0.618, 0.696) |
| LM-MOM | 0.584 (0.524, 0.641) | 0.595 (0.537, 0.650) | 0.565 (0.501, 0.627) | 0.582 (0.548, 0.616) |
| MM-RM | 0.669 (0.601, 0.730) | 0.747 (0.685, 0.801) | 0.665 (0.591, 0.731) | 0.695 (0.657, 0.731) |
| MM-MOM | 0.631 (0.568, 0.689) | 0.590 (0.526, 0.651) | 0.607 (0.542, 0.668) | 0.609 (0.572, 0.644) |
| LBT-RM | 0.801 (0.742, 0.850) | 0.826 (0.771, 0.870) | 0.788 (0.728, 0.838) | 0.805 (0.773, 0.834) |
| LBT-MOM | 0.715 (0.649, 0.773) | 0.736 (0.672, 0.791) | 0.724 (0.657, 0.782) | 0.725 (0.688, 0.759) |
| BM | 0.751 (0.687, 0.805) | 0.748 (0.685, 0.801) | 0.721 (0.652, 0.781) | 0.740 (0.703, 0.773) |
| Clinical model | 0.687 (0.618, 0.748) | 0.587 (0.516, 0.655) | 0.563 (0.487, 0.636) | 0.614 (0.573, 0.654) |
| BCM | 0.777 (0.714, 0.829) | 0.743 (0.679, 0.797) | 0.723 (0.652, 0.783) | 0.748 (0.712, 0.782) |
| LBTRBC-M | 0.910 (0.872, 0.937) | 0.920 (0.883, 0.946) | 0.897 (0.852, 0.929) | 0.909 (0.888, 0.926) |
| LBTMBC-M | 0.813 (0.755, 0.860) | 0.769 (0.710, 0.819) | 0.746 (0.682, 0.801) | 0.777 (0.743, 0.807) |
| LBTRB-M | 0.876 (0.830, 0.910) | 0.889 (0.847, 0.921) | 0.865 (0.815, 0.903) | 0.877 (0.853, 0.899) |
| LBTRC-M | 0.823 (0.764, 0.870) | 0.858 (0.811, 0.895) | 0.797 (0.737, 0.846) | 0.827 (0.797, 0.853) |

Data are mean (95% CI).

The results of test cohort 1, test cohort 2, test cohort 3, and the total test cohort corresponded to baseline, 1, years follow, up, 2, year follow, up, and encompassed the aforementioned follow, up time points. ROC: Receiver Operating Characteristic, JSN: Joint Space Narrowing, CI: Confidence Interval, FE-RM: Femur Radiomic Model, FC-RM: Femoral Cartilage Radiomic Model, TI-RM: Tibia Radiomic Model, TC-RM: Tibial Cartilage Radiomic Model, LM-RM: Lateral Meniscus Radiomic Model, MM-RM: Medial Meniscus Radiomic Model, LBT-RM: Load-Bearing Tissue Radiomic Model, BM: Biochemical biomarker Model, BCM: Biochemical biomarker plus Clinical variable Model, LBTRBC-M: Load-Bearing Tissue Radiomic plus Biochemical biomarker and Clinical variable Model, FE-MOM: Femur MOAKS Model, FC-MOM: Femoral Cartilage MOAKS Model, TI-RM: Tibia MOAKS Model, TC-MOM: Tibial Cartilage MOAKS Model, LM-MOM: Lateral Meniscus MOAKS Model, MM-MOM: Medial Meniscus MOAKS Model, LBT-MOM: Load-Bearing Tissue MOAKS Model, LBTMBC-M: Load-Bearing Tissue MOAKS plus Biochemical biomarker and Clinical variable Model, MOAKS: Magnetic resonance imaging OsteoArthritis Knee Score, LBTRC-M: Load-Bearing Tissue Radiomics plus Clinical variable Model, LBTRB-M: Load-Bearing Tissue Radiomics plus Biochemical biomarker Model.
