# Supplementary material for: Predicting knee osteoarthritis progression using neural network with longitudinal MRI radiomics, and biochemical biomarkers: A modeling study
Source: PLoS Med. 2025 Aug 21;22(8):e1004665. doi: 10.1371/journal.pmed.1004665 (PMC12370028; doi:10.1371/journal.pmed.1004665)
Supplement: S7 Table — The accuracy of predictive models in the test cohorts. (DOCX) [file pmed.1004665.s023.docx]

**Table S7. The accuracy of predictive models in the test cohorts.**

| **Predicting models** | **Test cohort 1** | **Test cohort 2** | **Test cohort 3** | **Total test cohort** |
| --- | --- | --- | --- | --- |
| FE-RM | 40.9% (123/301) | 48.2% (143/297) | 45.0% (125/278) | 44.6% (391/876) |
| FE-MOM | 44.5% (134/301) | 44.1% (131/297) | 41.4% (115/278) | 43.4% (380/876) |
| FC-RM | 32.6% (98/301) | 35.4% (105/297) | 30.9% (86/278) | 33.0% (289/876) |
| FC-MOM | 37.9% (114/301) | 38.4% (114/297) | 34.9% (97/278) | 37.1% (325/876) |
| TI-RM | 44.5% (134/301) | 47.5% (141/297) | 38.1% (106/278) | 43.5% (381/876) |
| TI-MOM | 42.9% (129/301) | 43.8% (130/297) | 41.7% (116/278) | 42.8% (375/876) |
| TC-RM | 42.2% (127/301) | 46.5% (138/297) | 48.2% (134/278) | 45.6% (399/876) |
| TC-MOM | 36.5% (110/301) | 39.7% (118/297) | 36.0% (100/278) | 37.4% (328/876) |
| LM-RM | 45.2% (136/301) | 46.5% (138/297) | 45.7% (127/278) | 45.8% (401/876) |
| LM-MOM | 30.9% (93/301) | 36.7% (109/297) | 31.7% (88/278) | 33.1% (290/876) |
| MM-RM | 43.9% (132/301) | 51.5% (153/297) | 42.8% (119/278) | 46.1% (404/876) |
| MM-MOM | 43.5% (128/301) | 41.1% (122/297) | 37.8% (105/278) | 40.5% (355/876) |
| LBT-RM | 58.5% (176/301) | 61.6% (183/297) | 54.0% (150/278) | 58.1% (509/876) |
| LBT-MOM | 51.5% (155/301) | 52.9% (157/297) | 48.6% (135/278) | 51.0% (447/876) |
| BM | 49.8% (150/301) | 49.8% (148/297) | 49.3% (137/278) | 49.7% (435/876) |
| Clinical model | 41.9% (126/301) | 40.7% (121/297) | 37.4% (104/278) | 40.1% (351/876) |
| BCM | 56.2% (169/301) | 52.5% (156/297) | 49.6% (138/278) | 52.9% (463/876) |
| LBTRBC-M | 68.1% (205/301) | 74.1% (220/297) | 68.0% (189/278) | 70.1% (614/876) |
| LBTMBC-M | 59.1% (178/301) | 54.2% (161/297) | 53.6% (149/278) | 55.7% (488/876) |

Data are percentages (numerator/denominator for percentages).

The results of test cohort 1, test cohort 2, test cohort 3, and the total test cohort corresponded to baseline, 1, years follow, up, 2, year follow, up, and encompassed the aforementioned follow, up time points. FE, RM: Femur Radiomic Model, FC, RM: Femoral Cartilage Radiomic Model, TI, RM: Tibia Radiomic Model, TC, RM: Tibial Cartilage Radiomic Model, LM, RM: Lateral Meniscus Radiomic Model, MM, RM: Medial Meniscus Radiomic Model, LBT, RM: Load, Bearing Tissue Radiomic Model, BM: Biochemical biomarker Model, BCM: Biochemical biomarker plus Clinical variable Model, LBTRBC, M: Load, Bearing Tissue Radiomic plus Biochemical biomarker and Clinical variable Model, FE, MOM: Femur MOAKS Model, FC, MOM: Femoral Cartilage MOAKS Model, TI, RM: Tibia MOAKS Model, TC, MOM: Tibial Cartilage MOAKS Model, LM, MOM: Lateral Meniscus MOAKS Model, MM, MOM: Medial Meniscus MOAKS Model, LBT, MOM: Load, Bearing Tissue MOAKS Model, LBTMBC, M: Load, Bearing Tissue MOAKS plus Biochemical biomarker and Clinical variable Model, MOAKS: Magnetic resonance imaging OsteoArthritis Knee Score, accuracy=(TP+TN)/(TP+FP +TN+FN), TP: True Positive, FP: False Positive, TN: True Negative, FN: False Negative.
