# Supplementary material for: Predicting knee osteoarthritis progression using neural network with longitudinal MRI radiomics, and biochemical biomarkers: A modeling study
Source: PLoS Med. 2025 Aug 21;22(8):e1004665. doi: 10.1371/journal.pmed.1004665 (PMC12370028; doi:10.1371/journal.pmed.1004665)
Supplement: S8 Table — Comparing the areas under two correlated ROC curves between predictive models in the test cohorts. (DOCX) [file pmed.1004665.s024.docx]

**Table S8. Comparing the areas under two correlated ROC curves between predictive models in the test cohorts.**

| **Predicting models** | **Tesr cohort 1** | |  | **Test cohort 2** | |  | **Test cohort 3** | |  | **Total test cohort** | |
| --- | --- | --- | --- | --- | --- | --- | --- | --- | --- | --- | --- |
|  | **ΔAUC** | ***p* value** |  | **ΔAUC** | ***p* value** |  | **ΔAUC** | ***p* value** |  | **ΔAUC** | ***p* value** |
| **JSN and pain progression** |  |  |  |  |  |  |  |  |  |  |  |
| FE-RM vs. FE-MOM | 0.011 (-0.075, 0.098) | 0.798 |  | 0.041 (-0.048, 0.129) | 0.368 |  | 0.111 (0.027, 0.195) | 0.010 |  | 0.053 (0.003, 0.103) | 0.036 |
| FC-RM vs. FC-MOM | -0.067 (-0.159, 0.025) | 0.152 |  | -0.031 (-0.124, 0.061) | 0.508 |  | -0.036 (-0.133, 0.061) | 0.469 |  | , 0.047 (-0.101, 0.007) | 0.086 |
| TI-RM vs. TI-MOM | 0.043 (-0.043, 0.129) | 0.329 |  | 0.118 (0.041, 0.196) | 0.003 |  | 0.051 (-0.033, 0.135) | 0.237 |  | 0.071 (0.023, 0.118) | 0.004 |
| TC-RM vs. TC-MOM | 0.073 (-0.002, 0.148) | 0.056 |  | 0.119 (0.033, 0.205) | 0.007 |  | 0.102 (0.019, 0.185) | 0.017 |  | 0.099 (0.052, 0.145) | <0.001 |
| LM-RM vs. LM-MOM | 0.145 (0.063, 0.227) | <0.001 |  | 0.143 (0.059, 0.227) | <0.001 |  | 0.130 (0.037, 0.223) | 0.006 |  | 0.139 (0.090, 0.189) | <0.001 |
| MM-RM vs. MM-MOM | 0.075 (-0.006, 0.157) | 0.070 |  | 0.164 (0.080, 0.249) | <0.001 |  | 0.120 (0.032, 0.208) | 0.007 |  | 0.120 (0.071, 0.169) | <0.001 |
| LBT-RM vs. FE-RM | 0.156 (0.094, 0.218) | <0.001 |  | 0.165 (0.101, 0.229) | <0.001 |  | 0.076 (0.010, 0.141) | 0.024 |  | 0.134 (0.098, 0.171) | <0.001 |
| LBT-RM vs. FC-RM | 0.261 (0.179, 0.344) | <0.001 |  | 0.281 (0.196, 0.365) | <0.001 |  | 0.243 (0.154, 0.332) | <0.001 |  | 0.265 (0.216, 0.314) | <0.001 |
| LBT-RM vs. TI-RM | 0.146 (0.072, 0.220) | <0.001 |  | 0.109 (0.041, 0.176) | 0.002 |  | 0.126 (0.053, 0.200) | <0.001 |  | 0.129 (0.087, 0.170) | <0.001 |
| LBT-RM vs. TC-RM | 0.116 (0.043, 0.189) | 0.002 |  | 0.154 (0.086, 0.221) | <0.001 |  | 0.107 (0.035, 0.179) | 0.004 |  | 0.127 (0.086, 0.167) | <0.001 |
| LBT-RM vs. LM-RM | 0.127 (0.056, 0.197) | <0.001 |  | 0.124 (0.051, 0.196) | <0.001 |  | 0.116 (0.048, 0.183) | <0.001 |  | 0.123 (0.083, 0.163) | <0.001 |
| LBT-RM vs. MM-RM | 0.135 (0.075, 0.196) | <0.001 |  | 0.104 (0.044, 0.165) | <0.001 |  | 0.142 (0.070, 0.213) | <0.001 |  | 0.128 (0.091, 0.165) | <0.001 |
| LBT-RM vs. LBT-MOM | 0.061 (-0.012, 0.135) | 0.102 |  | 0.099 (0.031, 0.166) | 0.004 |  | 0.059 (-0.013, 0.132) | 0.110 |  | 0.074 (0.033, 0.115) | <0.001 |
| LBT-RM vs. BM | 0.015 (-0.063, 0.092) | 0.709 |  | 0.100 (0.027, 0.173) | 0.007 |  | 0.031 (-0.044, 0.107) | 0.412 |  | 0.050 (0.007, 0.094) | 0.023 |
| LBT-RM vs. Clinical model | 0.126 (0.042, 0.209) | 0.003 |  | 0.180 (0.107, 0.252) | <0.001 |  | 0.144 (0.059, 0.229) | <0.001 |  | 0.151 (0.105, 0.198) | <0.001 |
| LBT-RM vs. BCM | -0.008 (-0.086, 0.070) | 0.837 |  | 0.074 (0.002, 0.146) | 0.044 |  | 0.016 (-0.062, 0.093) | 0.694 |  | 0.028 (-0.015, 0.072) | 0.203 |
| LBTRBC-M vs. LBTMBC-M | 0.067 (0.014, 0.121) | 0.014 |  | 0.119 (0.061, 0.176) | <0.001 |  | 0.109 (0.053, 0.165) | <0.001 |  | 0.096 (0.064, 0.128) | <0.001 |
| LBTRBC-M vs. BM | 0.092 (0.034, 0.151) | 0.002 |  | 0.163 (0.100, 0.226) | <0.001 |  | 0.115 (0.058, 0.172) | <0.001 |  | 0.122 (0.088, 0.157) | <0.001 |
| LBTRBC-M vs. Clinical model | 0.204 (0.132, 0.275) | <0.001 |  | 0.243 (0.176, 0.309) | <0.001 |  | 0.227 (0.156, 0.299) | <0.001 |  | 0.224 (0.183, 0.264) | <0.001 |
| LBTRBC-M vs. BCM | 0.070 (0.012, 0.127) | 0.018 |  | 0.137 (0.078, 0.197) | <0.001 |  | 0.100 (0.041, 0.158) | <0.001 |  | 0.100 (0.067, 0.134) | <0.001 |
| LBTRBC-M vs. LBTRB-M | 0.031 (-0.001, 0.061) | 0.252 |  | 0.023 (-0.001, 0.048) | 0.360 |  | 0.010 (-0.020, 0.040) | 0.534 |  | 0.021 (-0.005, 0.037) | 0.396 |
| LBTRBC-M vs. LBTRC-M | 0.042 (-0.001, 0.085) | 0.156 |  | 0.033 (-0.002, 0.064) | 0.173 |  | 0.075 (-0.037, 0.112) | 0.065 |  | 0.048 (-0.027, 0.070) | 0.132 |
| LBTRB-M vs. LBTRC-M | 0.011 (-0.032, 0.055) | 0.612 |  | 0.010 (-0.023, 0.043) | 0.562 |  | 0.065 (-0.024, 0.106) | 0.072 |  | 0.028 (-0.005, 0.050) | 0.201 |
| LBTRB-M vs. LBT-RM | 0.047 (-0.0001, 0.094) | 0.162 |  | 0.039 (-0.002, 0.081) | 0.203 |  | 0.074 (-0.027, 0.122) | 0.059 |  | 0.051 (-0.025, 0.077) | 0.105 |
| **JSN progression** |  |  |  |  |  |  |  |  |  |  |  |
| FE-RM vs. FEMOM | -0.005 (-0.120, 0.110) | 0.932 |  | 0.041 (-0.076, 0.158) | 0.495 |  | 0.163 (0.054, 0.272) | 0.003 |  | 0.069 (0.003, 0.135) | 0.040 |
| FC-RM vs. FC-MOM | -0.068 (-0.185, 0.049) | 0.253 |  | 0.031 (-0.081, 0.143) | 0.589 |  | 0.088 (-0.037, 0.213) | 0.167 |  | 0.022 (-0.046, 0.090) | 0.526 |
| TI-RM vs. TI-MOM | 0.108 (0.005, 0.21) | 0.040 |  | 0.179 (0.079, 0.279) | <0.001 |  | 0.070 (-0.032, 0.172) | 0.179 |  | 0.120 (0.061, 0.178) | <0.001 |
| TC-RM vs. TC-MOM | 0.058 (-0.047, 0.163) | 0.281 |  | 0.193 (0.081, 0.306) | <0.001 |  | 0.194 (0.087, 0.300) | <0.001 |  | 0.149 (0.086, 0.211) | <0.001 |
| LM-RM vs. LM-MOM | 0.161 (0.066, 0.256) | <0.001 |  | 0.101 (-0.003, 0.205) | 0.057 |  | 0.111 (0.005, 0.216) | 0.040 |  | 0.123 (0.064, 0.181) | <0.001 |
| MM-RM vs. MM-MOM | 0.049 (-0.049, 0.146) | 0.329 |  | 0.126 (0.032, 0.220) | 0.009 |  | 0.057 (-0.040, 0.155) | 0.247 |  | 0.077 (0.022, 0.133) | 0.006 |
| LBT-RM vs. FE-RM | 0.174 (0.084, 0.264) | <0.001 |  | 0.213 (0.118, 0.307) | <0.001 |  | 0.069 (-0.004, 0.142) | 0.063 |  | 0.149 (0.099, 0.199) | <0.001 |
| LBT-RM vs. FC-RM | 0.278 (0.178, 0.378) | <0.001 |  | 0.284 (0.199, 0.370) | <0.001 |  | 0.242 (0.130, 0.353) | <0.001 |  | 0.266 (0.208, 0.324) | <0.001 |
| LBT-RM vs. TI-RM | 0.108 (0.016, 0.200) | 0.021 |  | 0.146 (0.067, 0.225) | <0.001 |  | 0.201 (0.115, 0.286) | <0.001 |  | 0.151 (0.102, 0.200) | <0.001 |
| LBT-RM vs. TC-RM | 0.188 (0.099, 0.277) | <0.001 |  | 0.169 (0.088, 0.250) | <0.001 |  | 0.103 (0.018, 0.188) | 0.017 |  | 0.153 (0.104, 0.202) | <0.001 |
| LBT-RM vs. LM-RM | 0.100 (0.013, 0.187) | 0.024 |  | 0.194 (0.112, 0.275) | <0.001 |  | 0.166 (0.076, 0.257) | <0.001 |  | 0.154 (0.104, 0.203) | <0.001 |
| LBT-RM vs. MM-RM | 0.092 (0.009, 0.174) | 0.029 |  | 0.121 (0.042, 0.201) | 0.003 |  | 0.083 (-0.002, 0.167) | 0.055 |  | 0.098 (0.051, 0.145) | <0.001 |
| LBT-RM vs. LBT-MOM | 0.122 (0.032, 0.211) | 0.008 |  | 0.177 (0.086, 0.267) | <0.001 |  | 0.148 (0.061, 0.235) | <0.001 |  | 0.148 (0.097, 0.198) | <0.001 |
| LBT-RM vs. BM | 0.026 (-0.07, 0.123) | 0.592 |  | 0.090 (0.004, 0.177) | 0.041 |  | 0.068 (-0.038, 0.174) | 0.208 |  | 0.061 (0.006, 0.117) | 0.031 |
| LBT-RM vs. Clinical model | 0.072 (-0.021, 0.166) | 0.130 |  | 0.176 (0.078, 0.274) | <0.001 |  | 0.082 (-0.002, 0.165) | 0.054 |  | 0.109 (0.057, 0.162) | <0.001 |
| LBT-RM vs. BCM | -0.024 (-0.112, 0.064) | 0.599 |  | 0.071 (-0.019, 0.160) | 0.122 |  | 0.024 (-0.068, 0.117) | 0.605 |  | 0.023 (-0.029, 0.075) | 0.379 |
| LBTRBC-M vs. LBTMBC-M | 0.111 (0.044, 0.177) | 0.002 |  | 0.152 (0.078, 0.225) | <0.001 |  | 0.149 (0.072, 0.227) | <0.001 |  | 0.136 (0.094, 0.178) | <0.001 |
| LBTRBC-M vs. BM | 0.147 (0.063, 0.231) | <0.001 |  | 0.139 (0.062, 0.217) | <0.001 |  | 0.176 (0.080, 0.272) | <0.001 |  | 0.153 (0.104, 0.202) | <0.001 |
| LBTRBC-M vs. Clinical model | 0.193 (0.121, 0.265) | <0.001 |  | 0.225 (0.143, 0.307) | <0.001 |  | 0.189 (0.116, 0.263) | <0.001 |  | 0.201 (0.158, 0.245) | <0.001 |
| LBTRBC-M vs. BCM | 0.097 (0.030, 0.163) | 0.004 |  | 0.120 (0.050, 0.190) | <0.001 |  | 0.132 (0.052, 0.212) | 0.002 |  | 0.115 (0.074, 0.157) | <0.001 |
| LBTRBC-M vs. LBTRB-M | 0.081 (-0.036, 0.125) | 0.052 |  | 0.015 (-0.008, 0.038) | 0.210 |  | 0.048 (-0.024, 0.072) | 0.125 |  | 0.046 (-0.028, 0.064)) | 0.130 |
| LBTRBC-M vs. LBTRC-M | 0.064 (-0.024, 0.103) | 0.102 |  | 0.042 (-0.005, 0.079) | 0.206 |  | 0.059 (-0.026, 0.092) | 0.236 |  | 0.054 (-0.033, 0.075) | 0.247 |
| LBTRB-M vs. LBTRC-M | -0.017 (-0.065, 0.031) | 0.478 |  | 0.027 (-0.012, 0.066) | 0.170 |  | 0.011 (-0.024, 0.045) | 0.543 |  | 0.008 (-0.015, 0.031) | 0.588 |
| LBTRB-M vs. LBT-RM | 0.040 (-0.007, 0.086) | 0.195 |  | 0.034 (-0.022, 0.090) | 0.233 |  | 0.060 (-0.012, 0.107) | 0.072 |  | 0.045 (-0.017, 0.074) | 0.176 |
| **Pain progression** |  |  |  |  |  |  |  |  |  |  |  |
| FE-RM vs. FE-MOM | 0.157 (0.038, 0.275) | 0.010 |  | 0.095 (-0.029, 0.219) | 0.133 |  | 0.074 (-0.055, 0.204) | 0.260 |  | 0.109 (0.038, 0.180) | 0.003 |
| FC-RM vs. FC-MOM | -0.060 (-0.169, 0.048) | 0.276 |  | -0.027 (-0.152, 0.099) | 0.680 |  | -0.134 (-0.237, 0.032)) | 0.010 |  | -0.077 (-0.142, 0.013)) | 0.019 |
| TI-RM vs. TI-MOM | 0.161 (0.066, 0.255) | <0.001 |  | 0.127 (0.019, 0.235) | 0.022 |  | 0.059 (-0.038, 0.156) | 0.231 |  | 0.112 (0.054, 0.169) | <0.001 |
| TC-RM vs. TC-MOM | 0.125 (0.029, 0.222) | 0.011 |  | 0.119 (0.002, 0.235) | 0.046 |  | 0.136 (0.041, 0.231) | 0.005 |  | 0.126 (0.067, 0.185) | <0.001 |
| LM-RM vs. LM-MOM | 0.218 (0.111, 0.325) | <0.001 |  | 0.182 (0.057, 0.308) | 0.004 |  | 0.139 (0.029, 0.249) | 0.013 |  | 0.179 (0.113, 0.244) | <0.001 |
| MM-RM vs. MM-MOM | 0.017 (-0.094, 0.127) | 0.768 |  | 0.039 (-0.095, 0.173) | 0.570 |  | 0.061 (-0.045, 0.168) | 0.257 |  | 0.042 (-0.025, 0.108) | 0.220 |
| LBT-RM vs. FE-RM | 0.109 (0.036, 0.183) | 0.004 |  | 0.164 (0.065, 0.262) | 0.002 |  | 0.174 (0.081, 0.266) | <0.001 |  | 0.148 (0.097, 0.198) | <0.001 |
| LBT-RM vs. FC-RM | 0.249 (0.131, 0.367) | <0.001 |  | 0.272 (0.172, 0.373) | <0.001 |  | 0.254 (0.154, 0.354) | <0.001 |  | 0.257 (0.196, 0.319) | <0.001 |
| LBT-RM vs. TI-RM | 0.096 (-0.003, 0.195) | 0.057 |  | 0.142 (0.039, 0.245) | 0.007 |  | 0.150 (0.070, 0.229) | <0.001 |  | 0.131 (0.077, 0.184) | <0.001 |
| LBT-RM vs. TC-RM | 0.116 (0.045, 0.187) | 0.002 |  | 0.188 (0.093, 0.282) | <0.001 |  | 0.062 (-0.026, 0.150) | 0.167 |  | 0.119 (0.071, 0.167) | <0.001 |
| LBT-RM vs. LM-RM | 0.116 (0.023, 0.210) | 0.015 |  | 0.139 (0.034, 0.245) | 0.010 |  | 0.060 (-0.023, 0.143) | 0.158 |  | 0.103 (0.049, 0.157) | <0.001 |
| LBT-RM vs. MM-RM | 0.162 (0.065, 0.259) | 0.002 |  | 0.192 (0.093, 0.291) | <0.001 |  | 0.086 (-0.008, 0.181) | 0.074 |  | 0.141 (0.085, 0.198) | <0.001 |
| LBT-RM vs. LBT-MOM | 0.119 (0.027, 0.212) | 0.012 |  | 0.177 (0.071, 0.284) | 0.002 |  | 0.071 (-0.029, 0.172) | 0.165 |  | 0.119 (0.061, 0.177) | <0.001 |
| LBT-RM vs. BM | 0.040 (-0.051, 0.131) | 0.389 |  | 0.126 (0.032, 0.221) | 0.009 |  | 0.005 (-0.088, 0.098) | 0.916 |  | 0.053 (-0.001, 0.107) | 0.054 |
| LBT-RM vs. Clinical model | 0.057 (-0.035, 0.149) | 0.222 |  | 0.189 (0.074, 0.304) | 0.002 |  | 0.070 (-0.038, 0.179) | 0.202 |  | 0.099 (0.039, 0.160) | 0.002 |
| LBT-RM vs. BCM | -0.003 (-0.093, 0.087) | 0.948 |  | 0.114 (0.014, 0.215) | 0.026 |  | , 0.004 (-0.101, 0.093) | 0.936 |  | 0.030 (-0.026, 0.086) | 0.288 |
| LBTRBC-M vs. LBTMBC-M | 0.133 (0.058, 0.207) | <0.001 |  | 0.211 (0.123, 0.299) | <0.001 |  | 0.143 (0.075, 0.211) | <0.001 |  | 0.160 (0.116, 0.203) | <0.001 |
| LBTRBC-M vs. BM | 0.128 (0.053, 0.202) | <0.001 |  | 0.199 (0.122, 0.276) | <0.001 |  | 0.125 (0.046, 0.204) | 0.002 |  | 0.149 (0.105, 0.193) | <0.001 |
| LBTRBC-M vs. Clinical model | 0.145 (0.081, 0.210) | <0.001 |  | 0.261 (0.161, 0.362) | <0.001 |  | 0.190 (0.108, 0.273) | <0.001 |  | 0.195 (0.148, 0.243) | <0.001 |
| LBTRBC-M vs. BCM | 0.085 (0.020, 0.149) | 0.010 |  | 0.187 (0.104, 0.270) | <0.001 |  | 0.116 (0.043, 0.189) | 0.002 |  | 0.126 (0.084, 0.169) | <0.001 |
| LBTRBC-M vs. LBTRB-M | 0.028 (-0.014, 0.070) | 0.191 |  | 0.049 (-0.008, 0.090) | 0.158 |  | 0.044 (-0.008, 0.079) | 0.162 |  | 0.041 (-0.019, 0.064) | 0.174 |
| LBTRBC-M vs. LBTRC-M | 0.072 (-0.023, 0.121) | 0.079 |  | 0.056 (-0.015, 0.098) | 0.105 |  | 0.131 (-0.080, 0.181) | 0.053 |  | 0.089 (-0.061, 0.117) | 0.064 |
| LBTRB-M vs. LBTRC-M | 0.044 (-0.002, 0.086) | 0.109 |  | 0.007 (-0.045, 0.059) | 0.790 |  | 0.087 (-0.031, 0.143) | 0.055 |  | 0.048 (-0.018, 0.077) | 0.101 |
| LBTRB-M vs. LBT-RM | 0.060 (-0.015, 0.104) | 0.101 |  | 0.023 (-0.026, 0.072) | 0.350 |  | 0.076 (-0.020, 0.132) | 0.067 |  | 0.055 (-0.025, 0.084) | 0.105 |
| **Non progression** |  |  |  |  |  |  |  |  |  |  |  |
| FE-RM vs. FE-MOM | 0.023 (-0.061, 0.107) | 0.592 |  | 0.120 (0.041, 0.199) | 0.003 |  | 0.107 (0.020, 0.194) | 0.015 |  | 0.084 (0.036, 0.132) | <0.001 |
| FC-RM vs. FC-MOM | -0.068 (-0.160, 0.023) | 0.144 |  | -0.021 (-0.112, 0.069) | 0.646 |  | -0.007 (-0.099, 0.085) | 0.880 |  | , 0.034 (-0.087, 0.019) | 0.209 |
| TI-RM vs. TI-MOM | 0.046 (-0.029, 0.121) | 0.231 |  | 0.086 (0.009, 0.163) | 0.029 |  | 0.051 (-0.033, 0.135) | 0.237 |  | 0.060 (0.015, 0.106) | 0.009 |
| TC-RM vs. TC-MOM | 0.165 (0.076, 0.255) | <0.001 |  | 0.111 (0.019, 0.203) | 0.018 |  | 0.164 (0.078, 0.251) | <0.001 |  | 0.146 (0.095, 0.198) | <0.001 |
| LM-RM vs. LM-MOM | 0.065 (-0.031, 0.162) | 0.185 |  | 0.086 (0.002, 0.170) | 0.045 |  | 0.079 (-0.016, 0.174) | 0.103 |  | 0.076 (0.024, 0.129) | 0.004 |
| MM-RM vs. MM-MOM | 0.038 (-0.042, 0.119) | 0.351 |  | 0.157 (0.073, 0.241) | <0.001 |  | 0.058 (-0.035, 0.151) | 0.220 |  | 0.086 (0.037, 0.136) | <0.001 |
| LBT-RM vs. FE-RM | 0.144 (0.074, 0.214) | <0.001 |  | 0.084 (0.027, 0.141) | 0.004 |  | 0.072 (0.011, 0.134) | 0.020 |  | 0.100 (0.064, 0.136) | <0.001 |
| LBT-RM vs. FC-RM | 0.259 (0.175, 0.342) | <0.001 |  | 0.281 (0.199, 0.363) | <0.001 |  | 0.245 (0.158, 0.331) | <0.001 |  | 0.262 (0.214, 0.310) | <0.001 |
| LBT-RM vs. TI-RM | 0.121 (0.047, 0.196) | 0.002 |  | 0.130 (0.061, 0.200) | <0.001 |  | 0.123 (0.048, 0.198) | 0.002 |  | 0.126 (0.084, 0.168) | <0.001 |
| LBT-RM vs. TC-RM | 0.129 (0.057, 0.201) | <0.001 |  | 0.153 (0.081, 0.225) | <0.001 |  | 0.078 (0.011, 0.144) | 0.023 |  | 0.121 (0.081, 0.161) | <0.001 |
| LBT-RM vs. LM-RM | 0.152 (0.075, 0.230) | <0.001 |  | 0.145 (0.071, 0.220) | <0.001 |  | 0.144 (0.071, 0.217) | <0.001 |  | 0.147 (0.103, 0.190) | <0.001 |
| LBT-RM vs. MM-RM | 0.132 (0.071, 0.194) | <0.001 |  | 0.079 (0.016, 0.141) | 0.014 |  | 0.123 (0.053, 0.194) | <0.001 |  | 0.110 (0.073, 0.147) | <0.001 |
| LBT-RM vs. LBT-MOM | 0.086 (0.012, 0.160) | 0.023 |  | 0.090 (0.019, 0.161) | 0.013 |  | 0.064 (-0.014, 0.142) | 0.108 |  | 0.080 (0.037, 0.123) | <0.001 |
| LBT-RM vs. BM | 0.050 (-0.032, 0.133) | 0.232 |  | 0.078 (0.005, 0.151) | 0.035 |  | 0.067 (-0.017, 0.151) | 0.119 |  | 0.066 (0.020, 0.111) | 0.005 |
| LBT-RM vs. Clinical model | 0.115 (0.028, 0.201) | 0.009 |  | 0.238 (0.158, 0.318) | <0.001 |  | 0.225 (0.132, 0.318) | <0.001 |  | 0.191 (0.141, 0.241) | <0.001 |
| LBT-RM vs. BCM | 0.024 (-0.057, 0.106) | 0.560 |  | 0.083 (0.011, 0.155) | 0.024 |  | 0.066 (-0.020, 0.151) | 0.133 |  | 0.057 (0.011, 0.103) | 0.015 |
| LBTRBC-M vs. LBTMBC-M | 0.097 (0.039, 0.155) | 0.002 |  | 0.151 (0.096, 0.206) | <0.001 |  | 0.151 (0.092, 0.211) | <.001 |  | 0.132 (0.099, 0.165) | <0.001 |
| LBTRBC-M vs. BM | 0.159 (0.097, 0.221) | <0.001 |  | 0.172 (0.112, 0.232) | <0.001 |  | 0.176 (0.111, 0.241) | <.001 |  | 0.169 (0.134, 0.205) | <0.001 |
| LBTRBC-M vs. Clinical model | 0.223 (0.155, 0.292) | <0.001 |  | 0.333 (0.260, 0.405) | <0.001 |  | 0.334 (0.253, 0.415) | <.001 |  | 0.295 (0.252, 0.337) | <0.001 |
| LBTRBC-M vs. BCM | 0.133 (0.073, 0.193) | <0.001 |  | 0.177 (0.116, 0.238) | <0.001 |  | 0.174 (0.109, 0.239) | <.001 |  | 0.161 (0.125, 0.196) | <0.001 |
| LBTRBC-M vs. LBTRB-M | 0.034 (-0.008, 0.060) | 0.100 |  | 0.031 (-0.006, 0.055) | 0.105 |  | 0.032 (-0.007, 0.057) | 0.103 |  | 0.032 (-0.017, 0.046) | 0.102 |
| LBTRBC-M vs. LBTRC-M | 0.086 (-0.040, 0.132) | 0.055 |  | 0.062 (-0.031, 0.093) | 0.106 |  | 0.100 (-0.064, 0.136) | 0.052 |  | 0.082 (-0.061, 0.104) | 0.057 |
| LBTRB-M vs. LBTRC-M | 0.052 (-0.005, 0.099) | 0.092 |  | 0.031 (-0.004, 0.066) | 0.102 |  | 0.068 (-0.029, 0.108) | 0.067 |  | 0.051 (-0.027, 0.074) | 0.097 |
| LBTRB-M vs. LBT-RM | 0.074 (-0.027, 0.122) | 0.067 |  | 0.063 (-0.026, 0.101) | 0.104 |  | 0.077 (-0.033, 0.121) | 0.061 |  | 0.072 (-0.048, 0.097) | 0.077 |

Data are mean (95% CI).

The results of test cohort 1, test cohort 2, test cohort 3, and the total test cohort corresponded to baseline, 1, years follow, up, 2, year follow, up, and encompassed the aforementioned follow, up time points. ROC: Receiver Operating Characteristic, AUC: Area Under the ROC Curve, JSN: Joint Space Narrowing, CI: Confidence Interval, FE-RM: Femur Radiomic Model, FC-RM: Femoral Cartilage Radiomic Model, TI-RM: Tibia Radiomic Model, TC-RM: Tibial Cartilage Radiomic Model, LM-RM: Lateral Meniscus Radiomic Model, MM-RM: Medial Meniscus Radiomic Model, LBT-RM: Load-Bearing Tissue Radiomic Model, BM: Biochemical biomarker Model, BCM: Biochemical biomarker plus Clinical variable Model, LBTRBC-M: Load-Bearing Tissue Radiomics plus Biochemical biomarker and Clinical variable Model, FE-MOM: FEmur MOAKS Model, FC-MOM: Femoral Cartilage MOAKS Model, TI-RM: TIbia MOAKS Model, TC-MOM: Tibial Cartilage MOAKS Model, LM-MOM: Lateral Meniscus MOAKS Model, MM-MOM: Medial Meniscus MOAKS Model, LBT-MOM: Load-Bearing Tissue MOAKS Model, LBTMBC-M: Load-Bearing Tissue MOAKS plus Biochemical biomarker and Clinical variable Model, MOAKS: Magnetic resonance imaging OsteoArthritis Knee Score, LBTRB-M: Load-Bearing Tissue Radiomics plus Biochemical biomarker Model, LBTRC-M: Load-Bearing Tissue Radiomics plus Clinical variable Model.
