# Supplementary material for: Predicting knee osteoarthritis progression using neural network with longitudinal MRI radiomics, and biochemical biomarkers: A modeling study
Source: PLoS Med. 2025 Aug 21;22(8):e1004665. doi: 10.1371/journal.pmed.1004665 (PMC12370028; doi:10.1371/journal.pmed.1004665)
Supplement: S9 Table — Related risks of outcomes for predictive model outputs. (DOCX) [file pmed.1004665.s025.docx]

**Table S9. Related risks of outcomes for predictive model outputs.**

| **Predicting models** | **Crude OR** | ***p* value^†^** | **Adjusted OR^‡^** | ***p* value^†^** |
| --- | --- | --- | --- | --- |
| **FE-RM** |  |  |  |  |
| JSN and pain progression | 6.283 (4.927, 8.011) | <0.001 | 6.918 (5.371, 8.911) | <0.001 |
| JSN progression | 3.042 (2.315, 3.997) | <0.001 | 3.066 (2.308, 4.073) | <0.001 |
| Pain progression | 2.018 (1.531, 2.660) | <0.001 | 2.101 (1.584, 2.785) | <0.001 |
| Non progression | Reference | , | Reference | , |
| **FC-RM** |  |  |  |  |
| JSN and pain progression | 4.670 (3.699, 5.897) | <0.001 | 4.981 (3.916, 6.336) | <0.001 |
| JSN progression | 2.280 (1.741, 2.986) | <0.001 | 2.395 (1.814, 3.161) | <0.001 |
| Pain progression | 1.692 (1.275, 2.245) | <0.001 | 1.747 (1.309, 2.332) | <0.001 |
| Non progression | Reference | , | Reference | , |
| **TI-RM** |  |  |  |  |
| JSN and pain progression | 6.566 (5.155, 8.364) | <0.001 | 6.803 (5.306, 8.723) | <0.001 |
| JSN progression | 3.477 (2.616, 4.620) | <0.001 | 3.625 (2.694, 4.878) | <0.001 |
| Pain progression | 1.807 (1.373, 2.378) | <0.001 | 1.835 (1.388, 2.427) | <0.001 |
| Non progression | Reference | , | Reference | , |
| **TC-RM** |  |  |  |  |
| JSN and pain progression | 7.778 (6.077, 9.955) | <0.001 | 8.359 (6.457, 10.822) | <0.001 |
| JSN progression | 3.534 (2.672, 4.675) | <0.001 | 3.955 (2.941, 5.318) | <0.001 |
| Pain progression | 2.124 (1.602, 2.817) | <0.001 | 2.174 (1.630, 2.900) | <0.001 |
| Non progression | Reference | , | Reference | , |
| **LM-RM** |  |  |  |  |
| JSN and pain progression | 7.669 (5.983, 9.829) | <0.001 | 8.169 (6.321, 10.556) | <0.001 |
| JSN progression | 3.293 (2.502, 4.332) | <0.001 | 3.410 (2.567, 4.530) | <0.001 |
| Pain progression | 2.451 (1.847, 3.251) | <0.001 | 2.431 (1.826, 3.236) | <0.001 |
| Non progression | Reference | , | Reference | , |
| **MM-RM** |  |  |  |  |
| JSN and pain progression | 7.523 (5.897, 9.597) | <0.001 | 8.022 (6.222, 10.343) | <0.001 |
| JSN progression | 3.004 (2.266, 3.981) | <0.001 | 2.973 (2.224, 3.974) | <0.001 |
| Pain progression | 2.200 (1.656, 2.922) | <0.001 | 2.222 (1.665, 2.966) | <0.001 |
| Non progression | Reference | , | Reference | , |
| **LBT-RM** |  |  |  |  |
| JSN and pain progression | 13.882 (10.571, 18.230) | <0.001 | 14.705 (11.105, 19.472) | <0.001 |
| JSN progression | 4.703 (3.559, 6.215) | <0.001 | 4.936 (3.685, 6.612) | <0.001 |
| Pain progression | 2.371 (1.785, 3.149) | <0.001 | 2.427 (1.813, 3.250) | <0.001 |
| Non progression | Reference | , | Reference | , |
| **LBTBC-RM** |  |  |  |  |
| JSN and pain progression | 29.013 (21.277, 39.561) | <0.001 | 30.906 (22.470, 42.511) | <0.001 |
| JSN progression | 6.433 (4.784, 8.650) | <0.001 | 6.465 (4.740, 8.820) | <0.001 |
| Pain progression | 3.090 (2.321, 4.112) | <0.001 | 3.307 (2.457, 4.452) | <0.001 |
| Non progression | Reference | , | Reference | , |
| **Clinical model** |  |  |  |  |
| JSN and pain progression | 1.728 (1.398, 2.135) | <0.001 | 1.682 (1.316, 2.151) | <0.001 |
| JSN progression | 1.195 (0.827, 1.725) | 0.343 | 1.047 (0.664, 1.652) | 0.843 |
| Pain progression | 1.309 (0.826, 2.075) | 0.252 | 1.372 (0.852, 2.209) | 0.193 |
| Non progression | Reference | , | Reference | , |
| **Biochemical biomarker model** |  |  |  |  |
| JSN and pain progression | 2.941 (2.387, 3.622) | <0.001 | 2.952 (2.383, 3.656) | <0.001 |
| JSN progression | 1.741 (1.281, 2.366) | <0.001 | 1.584 (1.151, 2.182) | 0.005 |
| Pain progression | 1.382 (0.890, 2.146) | 0.150 | 1.359 (0.868, 2.127) | 0.180 |
| Non progression | Reference | , | Reference | , |
| **BCM** |  |  |  |  |
| JSN and pain progression | 3.290 (2.671, 4.053) | <0.001 | 3.291 (2.650, 4.086) | <0.001 |
| JSN progression | 1.917 (1.391, 2.643) | <0.001 | 1.743 (1.227, 2.475) | 0.002 |
| Pain progression | 1.478 (1.017, 2.146) | 0.040 | 1.527 (1.043, 2.235) | 0.030 |
| Non progression | Reference | , | Reference | , |

Data are OR (95% CI).

^†^GEE was used to assess the significance levels of individual risk factors, the outputs of predictive models were independent variables, KOA progression was dependent variable, non progression was the reference outcome.

^‡^ Adjustment for baseline age, sex, BMI, knee side, race, and WOMAC knee pain score.

KOA: Knee Osteoarthritis, OR: Odds Ratio, JSN: Joint Space Narrowing, BMI: Body Mass Index, GEE: Generalized Estimating Equation, CI: Confidence Interval, FE-RM: Femur Radiomic Model, FC-RM: Femoral Cartilage Radiomic Model, TI-RM: Tibia Radiomic Model, TC-RM: Tibial Cartilage Radiomic Model, LM-RM: Lateral Meniscus Radiomic Model, MM-RM: Medial Meniscus Radiomic Model, LBT-RM: Load-Bearing Tissue Radiomic Model, BCM: Biochemical biomarker plus Clinical variable Model, LBTRBC-M: Load-Bearing Tissue Radiomic plus Biochemical biomarker and Clinical variable Model, WOMAC: Western Ontario and McMaster Universities Arthritis Index.
