# Supplementary material for: Predicting knee osteoarthritis progression using neural network with longitudinal MRI radiomics, and biochemical biomarkers: A modeling study
Source: PLoS Med. 2025 Aug 21;22(8):e1004665. doi: 10.1371/journal.pmed.1004665 (PMC12370028; doi:10.1371/journal.pmed.1004665)
Supplement: S10 Table — Predictive performance of resident physicians under the assistance of LBTRBC-M. (DOCX) [file pmed.1004665.s026.docx]

**Table S10. Predictive performance of resident physicians under the assistance of LBTRBC-M.**

| **Predicting outcomes** | **Test cohort 1** | |  | **Test cohort 2** | |  | **Test cohort 3** | |  | **Total test cohort** | |
| --- | --- | --- | --- | --- | --- | --- | --- | --- | --- | --- | --- |
|  | **No** | **Yes** |  | **No** | **Yes** |  | **No** | **Yes** |  | **No** | **Yes** |
| **JSN and pain progression** |  |  |  |  |  |  |  |  |  |  |  |
| Sensitivity |  |  |  |  |  |  |  |  |  |  |  |
| Physician 1 | 47.2% (51/108) | 63.9% (69/108) |  | 44.1% (45/102) | 70.6% (72/102) |  | 48.4% (45/93) | 65.6% (61/93) |  | 46.5% (141/303) | 66.7% (202/303) |
| Physician 2 | 67.6% (73/108) | 68.5% (74/108) |  | 63.7% (65/102) | 71.6% (73/102) |  | 61.3% (57/93) | 71.0% (66/93) |  | 64.4% (195/303) | 70.3% (213/303) |
| Physician 3 | 58.3% (63/108) | 63.0% (68/108) |  | 62.7% (64/102) | 73.5% (75/102) |  | 58.1% (54/93) | 72.0% (67/93) |  | 59.7% (181/303) | 69.3% (210/303) |
| Physician 4 | 51.9% (56/108) | 62.0% (67/108) |  | 51.0% (52/102) | 63.7% (65/102) |  | 51.6% (48/93) | 67.7% (63/93) |  | 51.5% (156/303) | 64.4% (195/303) |
| Physician 5 | 60.2% (65/108) | 63.9% (69/108) |  | 59.8% (61/102) | 73.5% (75/102) |  | 57.0% (53/93) | 65.6% (61/93) |  | 59.1% (179/303) | 67.7% (205/303) |
| Physician 6 | 60.2% (65/108) | 64.8% (70/108) |  | 61.8% (63/102) | 74.5% (76/102) |  | 58.1% (54/93) | 69.9% (65/93) |  | 60.1% (182/303) | 69.6% (211/303) |
| Physician 7 | 61.1% (66/108) | 63.0% (68/108) |  | 62.7% (64/102) | 75.5% (77/102) |  | 59.1% (55/93) | 68.8% (64/93) |  | 61.1% (185/303) | 69.0% (209/303) |
| Specificity |  |  |  |  |  |  |  |  |  |  |  |
| Physician 1 | 52.6% (80/152) | 82.9% (126/152) |  | 52.8% (86/163) | 80.4% (131/163) |  | 53.9% (76/141) | 85.8% (121/141) |  | 53.1% (242/456) | 82.9% (378/456) |
| Physician 2 | 46.1% (70/152) | 76.3% (116/152) |  | 45.4% (74/163) | 82.8% (135/163) |  | 44.0% (62/141) | 76.6% (108/141) |  | 45.2% (206/456) | 78.7% (359/456) |
| Physician 3 | 50.7% (77/152) | 82.2% (125/152) |  | 52.8% (86/163) | 82.2% (134/163) |  | 51.1% (72/141) | 80.1% (113/141) |  | 51.5% (235/456) | 81.6% (372/456) |
| Physician 4 | 50.7% (77/152) | 79.6% (121/152) |  | 50.3% (82/163) | 76.7% (125/163) |  | 50.4% (71/141) | 79.4% (112/141) |  | 50.4% (230/456) | 78.5% (358/456) |
| Physician 5 | 56.6% (86/152) | 78.9% (120/152) |  | 54.6% (89/163) | 78.5% (128/163) |  | 52.5% (74/141) | 82.3% (116/141) |  | 54.6% (249/456) | 79.8% (364/456) |
| Physician 6 | 54.6% (83/152) | 80.9% (123/152) |  | 54.0% (88/163) | 79.8% (130/163) |  | 52.5% (74/141) | 80.1% (113/141) |  | 53.7% (245/456) | 80.3% (366/456) |
| Physician 7 | 53.3% (81/152) | 80.3% (122/152) |  | 54.0% (88/163) | 81.0% (132/163) |  | 55.3% (78/141) | 81.6% (115/141) |  | 54.2% (247/456) | 80.9% (369/456) |
| **JSN progression** |  |  |  |  |  |  |  |  |  |  |  |
| Sensitivity |  |  |  |  |  |  |  |  |  |  |  |
| Physician 1 | 4.0% (2/50) | 52.0% (26/50) |  | 3.9% (2/51) | 56.9% (29/51) |  | 8.0% (4/50) | 58.0% (29/50) |  | 5.3% (8/151) | 55.6% (84/151) |
| Physician 2 | 6.0% (3/50) | 38.0% (19/50) |  | 5.9% (3/51) | 52.9% (27/51) |  | 4.0% (2/50) | 44.0% (22/50) |  | 5.3% (8/151) | 45.0% (68/151) |
| Physician 3 | 8.0% (4/50) | 44.0% (22/50) |  | 5.9% (3/51) | 58.8% (30/51) |  | 2.0% (1/50) | 46.0% (23/50) |  | 5.3% (8/151) | 49.7% (75/151) |
| Physician 4 | 4.0% (2/50) | 44.0% (22/50) |  | 2.0% (1/51) | 54.9% (28/51) |  | 2.0% (1/50) | 48.0% (24/50) |  | 1.3% (2/151) | 49.0% (74/151) |
| Physician 5 | 18.0% (9/50) | 48.0% (24/50) |  | 13.7% (7/51) | 54.9% (28/51) |  | 6.0% (3/50) | 48.0% (24/50) |  | 12.6% (19/151) | 50.3% (76/151) |
| Physician 6 | 10.0% (5/50) | 56.0% (28/50) |  | 11.8% (6/51) | 54.9% (28/51) |  | 10.0% (5/50) | 46.0% (23/50) |  | 10.6% (16/151) | 52.3% (79/151) |
| Physician 7 | 8.0% (4/50) | 54.0% (27/50) |  | 11.8% (6/51) | 54.9% (28/51) |  | 10.0% (5/50) | 60.0% (30/50) |  | 9.9% (15/151) | 56.3% (85/151) |
| Specificity |  |  |  |  |  |  |  |  |  |  |  |
| Physician 1 | 67.9% (129/190) | 88.9% (169/190) |  | 65.5% (129/197)) | 88.3% (174/197) |  | 70.9% (117/165) | 92.7% (153/165) |  | 67.9% (375/552) | 89.9% (496/552) |
| Physician 2 | 73.7% (140/190) | 90.0% (171/190) |  | 69.0% (136/197) | 91.9% (181/197) |  | 70.9% (117/165) | 92.1% (152/165) |  | 71.2% (393/552) | 91.3% (504/552) |
| Physician 3 | 71.6% (136/190) | 90.0% (171/190) |  | 74.6% (147/197) | 90.9% (179/197) |  | 75.8% (125/165) | 95.2% (157/165) |  | 73.9% (408/552) | 91.8% (507/552) |
| Physician 4 | 68.9% (131/190) | 87.4% (166/190) |  | 68.0% (134/197) | 82.2% (162/197) |  | 72.1% (119/165) | 91.5% (151/165) |  | 69.6% (384/552) | 86.8% (479/552) |
| Physician 5 | 74.7% (142/190) | 86.8% (165/190) |  | 72.6% (143/197) | 88.8% (175/197) |  | 75.2% (124/165) | 92.7% (153/165) |  | 74.1% (409/552) | 89.3% (493/552) |
| Physician 6 | 75.3% (143/190) | 86.8% (165/190) |  | 73.6% (145/197) | 90.4% (178/197) |  | 74.5% (123/165) | 93.9% (155/165) |  | 74.5% (411/552) | 90.2% (498/552) |
| Physician 7 | 75.3% (143/190) | 85.8% (163/190) |  | 74.1% (146/197) | 91.9% (181/197) |  | 77.6% (128/165) | 90.3% (149/165) |  | 75.5% (417/552) | 89.3% (493/552) |
| **Pain progression** |  |  |  |  |  |  |  |  |  |  |  |
| Sensitivity |  |  |  |  |  |  |  |  |  |  |  |
| Physician 1 | 3.8% (2/52) | 44.2% (23/52) |  | 9.1% (4/44) | 40.9% (18/44) |  | 5.9% (3/51) | 31.4% (16/51) |  | 6.1% (9/147) | 38.8% (57/147) |
| Physician 2 | 5.8% (3/52) | 44.2% (23/52) |  | 11.4% (5/44) | 43.2% (19/44) |  | 5.9% (3/51) | 31.4% (16/51) |  | 7.5% (11/147) | 39.5% (58/147) |
| Physician 3 | 5.8% (3/52) | 40.4% (21/52) |  | 11.4% (5/44) | 34.1% (15/44) |  | 5.9% (3/51) | 35.3% (18/51) |  | 7.5% (11/147) | 36.7% (54/147) |
| Physician 4 | 1.9% (1/52) | 38.5% (20/52) |  | 4.5% (2/44) | 29.5% (13/44) |  | 2.0% (1/51) | 25.5% (13/51) |  | 2.0% (3/147) | 31.3% (46/147) |
| Physician 5 | 17.3% (9/52) | 38.5% (20/52) |  | 25.0% (11/44) | 40.9% (18/44) |  | 11.8% (6/51) | 37.3% (19/51) |  | 17.7% (26/147) | 38.8% (57/147) |
| Physician 6 | 13.5% (7/52) | 28.8% (15/52) |  | 9.1% (4/44) | 40.9% (18/44) |  | 7.8% (4/51) | 33.3% (17/51) |  | 10.2% (15/147) | 34.0% (50/147) |
| Physician 7 | 9.6% (5/52) | 30.8% (16/52) |  | 9.1% (4/44) | 40.9% (18/44) |  | 7.8% (4/51) | 27.5% (14/51) |  | 8.8% (13/147) | 32.7% (48/147) |
| Specificity |  |  |  |  |  |  |  |  |  |  |  |
| Physician 1 | 66.5% (129/194) | 88.7% (172/194) |  | 61.7% (127/206) | 89.8% (185/206) |  | 65.9% (118/179) | 92.7% (166/179) |  | 64.6% (374/579) | 90.3% (523/579) |
| Physician 2 | 72.2% (140/194) | 86.1% (167/194) |  | 65.0% (134/206) | 91.7% (189/206) |  | 64.8% (116/179) | 88.3% (158/179) |  | 67.4% (390/579) | 88.8% (514/579) |
| Physician 3 | 70.6% (137/194) | 88.7% (172/194) |  | 70.4% (145/206) | 94.2% (194/206) |  | 68.7% (123/179) | 90.5% (162/179) |  | 69.9% (405/579) | 91.2% (528/579) |
| Physician 4 | 68.6% (133/194) | 86.6% (168/194) |  | 64.1% (132/206) | 85.9% (177/206) |  | 65.9% (118/179) | 90.5% (162/179) |  | 66.1% (383/579) | 87.6% (507/579) |
| Physician 5 | 73.2% (142/194) | 87.1% (169/194) |  | 67.5% (139/206) | 89.8% (185/206) |  | 67.6% (121/179) | 88.3% (158/179) |  | 69.4% (402/579) | 88.4% (512/579) |
| Physician 6 | 72.7% (141/194) | 91.8% (178/194) |  | 71.4% (147/206) | 91.3% (188/206) |  | 69.3% (124/179) | 89.9% (161/179) |  | 71.2% (412/579) | 91.0% (527/579) |
| Physician 7 | 73.2% (142/194) | 89.7% (174/194) |  | 71.8% (148/206) | 92.7% (191/206) |  | 72.1% (129/179) | 92.2% (165/179) |  | 72.4% (419/579) | 91.5% (530/579) |
| **Non progression** |  |  |  |  |  |  |  |  |  |  |  |
| Sensitivity |  |  |  |  |  |  |  |  |  |  |  |
| Physician 1 | 83.5% (76/91) | 84.6% (77/91) |  | 80.0% (80/100) | 84.0% (84/100) |  | 82.1% (69/84) | 90.5% (76/84) |  | 81.8% (225/275) | 86.2% (237/275) |
| Physician 2 | 70.3% (64/91) | 81.3% (74/91) |  | 66.0% (66/100) | 89.0% (89/100) |  | 67.9% (57/84) | 83.3% (70/84) |  | 68.0% (187/275) | 84.7% (233/275) |
| Physician 3 | 76.9% (70/91) | 90.1% (82/91) |  | 78.0% (78/100) | 89.0% (89/100) |  | 81.0% (68/84) | 85.7% (72/84) |  | 78.5% (216/275) | 88.4% (243/275) |
| Physician 4 | 82.4% (75/91) | 86.8% (79/91) |  | 80.0% (80/100) | 84.0% (84/100) |  | 83.3% (70/84) | 89.3% (75/84) |  | 81.8% (225/275) | 86.5% (238/275) |
| Physician 5 | 74.7% (68/91) | 83.5% (76/91) |  | 71.0% (71/100) | 82.0% (82/100) |  | 77.4% (65/84) | 86.9% (73/84) |  | 74.2% (204/275) | 84.0% (231/275) |
| Physician 6 | 78.0% (71/91) | 87.9% (80/91) |  | 78.0% (78/100) | 84.0% (84/100) |  | 77.4% (65/84) | 86.9% (73/84) |  | 77.8% (214/275) | 86.2% (237/275) |
| Physician 7 | 79.1% (72/91) | 86.8% (79/91) |  | 78.0% (78/100) | 86.0% (86/100) |  | 82.1% (69/84) | 84.5% (71/84) |  | 79.6% (219/275) | 85.8% (236/275) |
| Specificity |  |  |  |  |  |  |  |  |  |  |  |
| Physician 1 | 31.4% (55/175) | 67.4% (118/175) |  | 29.8% (51/171) | 69.6% (119/171) |  | 30.4% (52/171) | 62.0% (106/171) |  | 30.6% (158/517) | 66.3% (343/517) |
| Physician 2 | 45.1% (79/175) | 66.3% (116/175) |  | 42.7% (73/171) | 69.6% (119/171) |  | 36.3% (62/171) | 60.8% (104/171) |  | 41.4% (214/517) | 65.6% (339/517) |
| Physician 3 | 40.0% (70/175) | 63.4% (111/175) |  | 42.1% (72/171) | 70.2% (120/171) |  | 33.9% (58/171) | 63.2% (108/171) |  | 38.7% (200/517) | 65.6% (339/517) |
| Physician 4 | 33.1% (58/175) | 62.3% (109/175) |  | 31.6% (54/171) | 62.0% (106/171) |  | 28.7% (49/171) | 58.5% (100/171) |  | 31.1% (161/517) | 60.9% (315/517) |
| Physician 5 | 47.4% (83/175) | 64.6% (113/175) |  | 46.2% (79/171) | 70.8% (121/171) |  | 36.3% (62/171) | 60.8% (104/171) |  | 43.3% (224/517) | 65.4% (338/517) |
| Physician 6 | 44.0% (77/175) | 64.6% (113/175) |  | 42.7% (73/171) | 71.3% (122/171) |  | 36.8% (63/171) | 61.4% (105/171) |  | 41.2% (213/517) | 65.8% (340/517) |
| Physician 7 | 42.9% (75/175) | 63.4% (111/175) |  | 43.3% (74/171) | 71.9% (123/171) |  | 37.4% (64/171) | 63.2% (108/171) |  | 41.2% (213/517) | 66.2% (342/517) |

Data are percentages (numerator/denominator for percentages). Clinical practice years of resident physicians: 1 to 4 years.

The results of test cohort 1, test cohort 2, test cohort 3, and the total test cohort corresponded to baseline, 1, years follow, up, 2, year follow, up, and encompassed the aforementioned follow, up time points. LBTRBC-M: Load-Bearing Tissue Radiomic plus Biochemical biomarker and Clinical variable Model, sensitivity=TP/(TP+FN), specificity=TN/(TN+FP), TP: True Positive, TN: True Negative, FP: False Positive, FN: False Negative.
