# Supplementary material for: Predicting knee osteoarthritis progression using neural network with longitudinal MRI radiomics, and biochemical biomarkers: A modeling study
Source: PLoS Med. 2025 Aug 21;22(8):e1004665. doi: 10.1371/journal.pmed.1004665 (PMC12370028; doi:10.1371/journal.pmed.1004665)
Supplement: S11 Table — The accuracy of resident physicians under the assistance of LBTRBC-M. (DOCX) [file pmed.1004665.s027.docx]

**Table S11. The accuracy of resident physicians under the assistance of LBTRBC-M.**

| **Resident physicians** | **Test cohort 1** | |  | **Test cohort 2** | |  | **Test cohort 3** | |  | **Total test cohort** | |
| --- | --- | --- | --- | --- | --- | --- | --- | --- | --- | --- | --- |
|  | **No** | **Yes** |  | **No** | **Yes** |  | **No** | **Yes** |  | **No** | **Yes** |
| Physician 1 | 43.5% (131/301) | 64.8% (195/301) |  | 44.1% (131/297) | 68.4% (203/297) |  | 43.5% (121/278) | 65.5% (182/278) |  | 43.7% (383/876) | 66.2% (580/876) |
| Physician 2 | 47.5% (143/301) | 63.1% (190/301) |  | 46.8% (139/297) | 70.0% (208/297) |  | 42.8% (119/278) | 62.6% (174/278) |  | 45.8% (401/876) | 65.3% (572/876) |
| Physician 3 | 46.5% (140/301) | 64.1% (193/301) |  | 50.5% (150/297) | 70.4% (209/297) |  | 45.3% (126/278) | 64.8% (180/278) |  | 47.5% (416/876) | 66.4% (582/876) |
| Physician 4 | 44.2% (133/301) | 62.5% (188/301) |  | 45.1% (134/297) | 64.0% (190/297) |  | 42.8% (119/278) | 63.0% (175/278) |  | 44.1% (386/876) | 63.1% (553/876) |
| Physician 5 | 50.2% (151/301) | 62.8% (189/301) |  | 50.5% (150/297) | 68.4% (203/297) |  | 45.7% (127/278) | 63.7% (169/278) |  | 48.9% (428/876) | 65.0% (569/876) |
| Physician 6 | 49.2% (148/301) | 64.1% (193/301) |  | 50.8% (151/297) | 69.4% (206/297) |  | 46.0% (128/278) | 64.0% (178/278) |  | 48.7% (427/876) | 65.9% (577/876) |
| Physician 7 | 48.8% (147/301) | 63.1% (190/301) |  | 51.2% (152/297) | 70.4% (209/297) |  | 47.8% (133/278) | 64.4% (179/278) |  | 49.3% (432/876) | 66.0% (578/876) |

Data are percentages (numerator/denominator for percentages). Clinical practice years of resident physicians: 1 to 4 years.

The results of test cohort 1, test cohort 2, test cohort 3, and the total test cohort corresponded to baseline, 1, years follow, up, 2, year follow, up, and encompassed the aforementioned follow, up time points. LBTRBC-M: Load-Bearing Tissue Radiomic plus Biochemical biomarker and Clinical variable Model, accuracy=(TP+TN)/(TP+FP +TN+FN), TP: True Positive, FP: False Positive, TN: True Negative, FN: False Negative.
