# Supplementary material for: Predicting knee osteoarthritis progression using neural network with longitudinal MRI radiomics, and biochemical biomarkers: A modeling study
Source: PLoS Med. 2025 Aug 21;22(8):e1004665. doi: 10.1371/journal.pmed.1004665 (PMC12370028; doi:10.1371/journal.pmed.1004665)
Supplement: S12 Table — Predictive performance of resident physicians under the assistance of LBTRBC-M in the test cohorts. (DOCX) [file pmed.1004665.s028.docx]

**Table S12. Predictive performance of resident physicians under the assistance of LBTRBC-M in the test cohorts.**

| **Resident physicians (n=7)** |  | **Test cohort 1** | | |  | **Test cohort 2** | | |  | **Test cohort 3** | | |  | **Total test cohort** | | |
| --- | --- | --- | --- | --- | --- | --- | --- | --- | --- | --- | --- | --- | --- | --- | --- | --- |
| **Predictive performance** |  | **No** | **Yes** | ***p* value** |  | **No** | **Yes** | ***p* value** |  | **No** | **Yes** | ***p* value** |  | **No** | **Yes** | ***p* value** |
| **Accuracy** |  | 47.1% (44.8%, 49.5%) | 63.5% (62.7%, 64.3%) | <0.001 |  | 48.4% (45.6%, 51.2%) | 68.7% (66.6%, 70.8%) | <0.001 |  | 44.8% (43.1%, 46.6%) | 64.0% (63.1%, 64.9%) | <0.001 |  | 46.9% (44.7%, 49.0%) | 65.4% (64.4%, 66.5%) | <.001 |
| **JSN and pain progression** |  |  |  |  |  |  |  |  |  |  |  |  |  |  |  |  |
| Sensitivity |  | 58.1% (51.9%, 64.2%) | 64.2% (62.2%, 66.1%) | 0.039 |  | 58.0% (51.0%, 64.9%) | 71.8% (68.2%, 75.5%) | <0.001 |  | 56.2% (52.0%, 60.4%) | 68.7% (66.3%, 71.0%) | <0.001 |  | 57.5% (51.7%, 63.2%) | 68.1% (66.2%, 70.0%) | <.001 |
| Specificity |  | 52.1% (48.9%, 55.2%) | 80.2% (78.1%, 82.2%) | <0.001 |  | 52.0% (49.0%, 55.0%) | 80.2% (78.2%, 82.2%) | <0.001 |  | 51.4% (48.0%, 54.8%) | 80.9% (78.2%, 83.5%) | <0.001 |  | 51.8% (48.8%, 54.9%) | 80.4% (78.9%, 81.8%) | <.001 |
| **JSN progression** |  |  |  |  |  |  |  |  |  |  |  |  |  |  |  |  |
| Sensitivity |  | 8.3% (3.8%, 12.7%) | 48.0% (42.1%, 53.9%) | <0.001 |  | 7.6% (2.9%, 12.2%) | 55.5% (53.7%, 57.2%) | <0.001 |  | 5.7% (2.1%, 9.3%) | 50.0% (44.2%, 55.8%) | <0.001 |  | 7.2% (3.5%, 10.8%) | 51.2% (47.5%, 54.8%) | <.001 |
| Specificity |  | 72.5% (69.6%, 75.3%) | 88.0% (86.4%, 89.5%) | <0.001 |  | 71.1% (67.8%, 74.3%) | 89.2% (86.1%, 92.3%) | <0.001 |  | 73.9% (71.5%, 76.2%) | 92.6% (91.2%, 94.1%) | <0.001 |  | 72.4% (69.8%, 75.0%) | 89.8% (88.3%, 91.3%) | <.001 |
| **Pain progression** |  |  |  |  |  |  |  |  |  |  |  |  |  |  |  |  |
| Sensitivity |  | 8.0% (2.5%, 13.4%) | 37.9% (32.3%, 43.5%) | <0.001 |  | 11.4% (5.4%, 17.3%) | 38.6% (34.1%, 43.2%) | <0.001 |  | 6.7% (4.0%, 9.5%) | 31.7% (27.8%, 35.5%) | <0.001 |  | 8.6% (4.1%, 13.0%) | 36.0% (32.9%, 39.0%) | <.001 |
| Specificity |  | 71.0% (68.6%, 73.4%) | 88.4% (86.5%, 90.2%) | <0.001 |  | 67.4% (63.7%, 71.1%) | 90.8% (88.3%, 93.2%) | <0.001 |  | 67.8% (65.5%, 70.1%) | 90.3% (88.7%, 91.9%) | <0.001 |  | 68.7% (66.1%, 71.3%) | 89.8% (88.4%, 91.3%) | <.001 |
| **Non progression** |  |  |  |  |  |  |  |  |  |  |  |  |  |  |  |  |
| Sensitivity |  | 77.9% (73.7%, 82.0%) | 85.9% (83.2%, 88.6%) | 0.002 |  | 75.9% (71.0%, 80.8%) | 85.4% (82.9%, 87.9%) | 0.002 |  | 78.7% (73.8%, 83.7%) | 86.7% (84.4%, 89.1%) | 0.004 |  | 77.4% (72.9%, 81.9%) | 86.0% (84.7%, 87.3%) | <.001 |
| Specificity |  | 40.6% (34.9%, 46.2%) | 64.6% (62.9%, 66.2%) | <0.001 |  | 39.8% (33.9%, 45.6%) | 69.3% (66.2%, 72.4%) | <0.001 |  | 34.3% (31.1%, 37.4%) | 61.4% (59.9%, 62.9%) | <0.001 |  | 38.2% (33.4%, 43.0%) | 65.1% (63.4%, 66.8%) | <.001 |

Data are mean (95% CI). Clinical practice years of resident physicians: 1 to 4 years.

The results of test cohort 1, test cohort 2, test cohort 3, and the total test cohort corresponded to baseline, 1, years follow, up, 2, year follow, up, and encompassed the aforementioned follow, up time points. CI: Confidence Interval, LBTRBC-M: Load-Bearing Tissue Radiomic plus Biochemical biomarker and Clinical variable Model, JSN: Joint Space Narrowing, sensitivity=TP/(TP+FN), specificity=TN/(TN+FP), accuracy=(TP+TN)/(TP+FP +TN+FN), TP: True Positive, TN: True Negative, FP: False Positive, FN: False Negative.
