# Supplementary material for: Predicting knee osteoarthritis progression using neural network with longitudinal MRI radiomics, and biochemical biomarkers: A modeling study
Source: PLoS Med. 2025 Aug 21;22(8):e1004665. doi: 10.1371/journal.pmed.1004665 (PMC12370028; doi:10.1371/journal.pmed.1004665)
Supplement: S13 Table — Related risks of outcomes for LBTRBC-M outputs using different GEE model. (DOCX) [file pmed.1004665.s029.docx]

**Table S13. Related risks of outcomes for LBTRBC-M outputs using different GEE model.**

| **GEE structure** | **Crude OR** | ***p* value^†^** | **Adjusted OR^‡^** | ***p* value^†^** |
| --- | --- | --- | --- | --- |
| **Autoregressive** |  |  |  |  |
| JSN and pain progression | 29.013 (21.277, 39.561) | <0.001 | 30.906 (22.470, 42.511) | <0.001 |
| JSN progression | 6.433 (4.784, 8.650) | <0.001 | 6.465 (4.740, 8.820) | <0.001 |
| Pain progression | 3.090 (2.321, 4.112) | <0.001 | 3.307 (2.457, 4.452) | <0.001 |
| Non progression | Reference | , | Reference | , |
| **Independent** |  |  |  |  |
| JSN and pain progression | 28.512 (20.843, 39.014) | <0.001 | 30.165 (22.161, 42.051) | <0.001 |
| JSN progression | 6.457 (4.774, 8.612) | <0.001 | 6.418 (4.652, 8.806) | <0.001 |
| Pain progression | 3.167 (2.389, 4.243) | <0.001 | 3.338 (2.595, 4.580) | <0.001 |
| Non progression | Reference | , | Reference | , |
| **Exchangeable** |  |  |  |  |
| JSN and pain progression | 29.008 (21.392, 40.274) | <0.001 | 30.219 (22.880, 43.524) | <0.001 |
| JSN progression | 6.548 (4.909, 8.769) | <0.001 | 6.642 (4.896, 8.934) | <0.001 |
| Pain progression | 3.218 (2.449, 4.344) | <0.001 | 3.428 (2.695, 4.635) | <0.001 |
| Non progression | Reference | , | Reference | , |
| **Unstructured** |  |  |  |  |
| JSN and pain progression | 28.965 (20.993, 39.731) | <0.001 | 30.736 (22.470, 42.962) | <0.001 |
| JSN progression | 6.321 (4.748, 8.541) | <0.001 | 6.552 (4.779, 8.854) | <0.001 |
| Pain progression | 3.131 (2.349, 4.160) | <0.001 | 3.353 (2.579, 4.586) | <0.001 |
| Non progression | Reference | , | Reference | , |

Data are OR (95% CI).

†GEE was used to assess the significance levels of individual risk factors, the outputs of predictive models were independent variables, KOA progression was dependent variable, non progression was the reference outcome.

‡ Adjustment for baseline age, sex, BMI, knee side, race, and WOMAC knee pain score.

KOA: Knee Osteoarthritis, OR: Odds Ratio, JSN: Joint Space Narrowing, BMI: Body Mass Index, GEE: Generalized Estimating Equation, CI: Confidence Interval, LBTRBC-M: Load-Bearing Tissue Radiomic plus Biochemical biomarker and Clinical variable Model, WOMAC: Western Ontario and McMaster Universities Arthritis Index.
