# Supplementary material for: Predicting knee osteoarthritis progression using neural network with longitudinal MRI radiomics, and biochemical biomarkers: A modeling study
Source: PLoS Med. 2025 Aug 21;22(8):e1004665. doi: 10.1371/journal.pmed.1004665 (PMC12370028; doi:10.1371/journal.pmed.1004665)
Supplement: S14 Table — Compares the predictive performance of the LBTRBC-M model using/without using the MI in the total test cohort. (DOCX) [file pmed.1004665.s030.docx]

**Table S14. Compares the predictive performance of the LBTRBC-M model using/without using the MI in the total test cohort.**

| **Predicting models** | **Using MI*** | **Without using MI*** | **ΔAUC** | ***p* value** |
| --- | --- | --- | --- | --- |
| JSN and pain progression | 0.880 (0.853, 0.903) | 0.866 (0.839, 0.889) | 0.014 (-0.002, 0.030) | 0.081 |
| JSN progression | 0.913 (0.881, 0.937) | 0.886 (0.851, 0.913) | 0.028 (-0.011, 0.044) | 0.072 |
| Pain progression | 0.886 (0.856, 0.910) | 0.871 (0.840, 0.897) | 0.015 (-0.006, 0.035) | 0.160 |
| Non progression | 0.909 (0.888, 0.926) | 0.886 (0.860, 0.907) | 0.023 (-0.009, 0.037) | 0.075 |

*Data are mean AUC (95% CI).

AUC: Areas Under receiver operating characteristic Curve, MI: Multiple Imputation, CI: Confidence Interval, LBTRBC-M: Load-Bearing Tissue Radiomic plus Biochemical biomarker and Clinical variable Model.
