# Supplementary material for: Predicting knee osteoarthritis progression using neural network with longitudinal MRI radiomics, and biochemical biomarkers: A modeling study
Source: PLoS Med. 2025 Aug 21;22(8):e1004665. doi: 10.1371/journal.pmed.1004665 (PMC12370028; doi:10.1371/journal.pmed.1004665)
Supplement: S15 Table — Comparing predictive performance of the LBTRBC-M model using different algorithms in the total test cohort. (DOCX) [file pmed.1004665.s031.docx]

**Table S15. Comparing predictive performance of the LBTRBC**-**M model using different algorithms in the total test cohort.**

| **Algorithm** | **Total test cohort** | | | |
| --- | --- | --- | --- | --- |
|  | **ERS** | **MR** | **AUC** | **RASE** |
| XGBoost | 0.409 | 0.299 | 0.897 | 0.508 |
| Bootstrap Forest | 0.179 | 0.403 | 0.833 | 0.654 |
| Shallow Neural Network | 0.118 | 0.418 | 0.807 | 0.661 |
| Support Vector Machines | 0.089 | 0.422 | 0.804 | 0.691 |
| Decision Tree | 0.013 | 0.595 | 0.575 | 0.708 |
| Nominal Logistic | -2.110 | 0.502 | 0.677 | 0.716 |
| Naive Bayes | -5.538 | 0.654 | 0.659 | 0.792 |

ERS: Entropy R Square, MR: Misclassification Rate, AUC: Area Under receiver operating characteristic Curve, RASE: Root Average Squared Error, LBTRBC-M: Load-Bearing Tissue Radiomic plus Biochemical biomarker and Clinical variable Model.
