# Supplementary material for: Predicting knee osteoarthritis progression using neural network with longitudinal MRI radiomics, and biochemical biomarkers: A modeling study
Source: PLoS Med. 2025 Aug 21;22(8):e1004665. doi: 10.1371/journal.pmed.1004665 (PMC12370028; doi:10.1371/journal.pmed.1004665)
Supplement: S16 Table — The parameters of LBTRBA-M model. (DOCX) [file pmed.1004665.s032.docx]

**Table S16. The parameters of LBTRBA**-**M model.**

| **Model parameters** | **Values** | **Descriptions** | **Source cited** |
| --- | --- | --- | --- |
| Entropy R Square | 0.409 | Measures the proportion of the variation in the data that is explained by the model. Higher values indicate better fit. | Chen, Tianqi, and Carlos Guestrin. "Xgboost: A scalable tree boosting system." Proceedings of the 22nd acm sigkdd international conference on knowledge discovery and data mining. 2016. |
| Misclassification Rate | 0.299 | The proportion of incorrectly classified predictions in the model. Lower values indicate better performance. | Chen, Tianqi, and Carlos Guestrin. "Xgboost: A scalable tree boosting system." *Proceedings of the 22nd acm sigkdd international conference on knowledge discovery and data mining*. 2016. |
| AUC | 0.897 | Area Under the Receiver Operating Characteristic Curve, which reflects the model's ability to discriminate between classes. A value close to 1 indicates excellent discrimination. | Chen, Tianqi, and Carlos Guestrin. "Xgboost: A scalable tree boosting system." *Proceedings of the 22nd acm sigkdd international conference on knowledge discovery and data mining*. 2016. |
| Root Average Squared Error | 0.508 | A measure of the average magnitude of errors in prediction, with lower values indicating more accurate predictions. | Chen, Tianqi, and Carlos Guestrin. "Xgboost: A scalable tree boosting system." *Proceedings of the 22nd acm sigkdd international conference on knowledge discovery and data mining*. 2016. |
| Logloss | 0.792 | Logarithmic loss, indicating how well the predicted probabilities match the actual outcomes. Lower values reflect better model performance. | Chen, Tianqi, and Carlos Guestrin. "Xgboost: A scalable tree boosting system." *Proceedings of the 22nd acm sigkdd international conference on knowledge discovery and data mining*. 2016. |
| Accuracy | 0.701 | The proportion of correct predictions made by the model, with higher values indicating better predictive accuracy. | Chen, Tianqi, and Carlos Guestrin. "Xgboost: A scalable tree boosting system." *Proceedings of the 22nd acm sigkdd international conference on knowledge discovery and data mining*. 2016. |
| F1 score | 0.665 | The harmonic mean of precision and recall, providing a balance between the two. A value closer to 1 indicates better performance. | Sokolova, Marina, and Guy Lapalme. "A systematic analysis of performance measures for classification tasks." Information processing & management 45.4 (2009): 427, 437. |
| Matthews Correlation Coefficient | 0.578 | A balanced measure that takes into account true and false positives and negatives. A value closer to 1 indicates a better model. | Chicco, Davide, and Giuseppe Jurman. "A statistical comparison between Matthews correlation coefficient (MCC), prevalence threshold, and Fowlkes–Mallows index." Journal of Biomedical Informatics 144 (2023): 104426. |
| Max_depth | 6 | The maximum depth of the decision tree. A higher value allows the model to capture more complex relationships but may risk overfitting. | Chen, Tianqi, and Carlos Guestrin. "Xgboost: A scalable tree boosting system." *Proceedings of the 22nd acm sigkdd international conference on knowledge discovery and data mining*. 2016. |
| Subsample | 1 | The fraction of samples used for fitting each tree. A value of 1 means no sampling and all data is used. Lower values help prevent overfitting. | Chen, Tianqi, and Carlos Guestrin. "Xgboost: A scalable tree boosting system." *Proceedings of the 22nd acm sigkdd international conference on knowledge discovery and data mining*. 2016. |
| Colsample_bytree | 1 | The fraction of features used per tree. A value of 1 indicates that all features are used in the model. Lower values reduce overfitting. | Chen, Tianqi, and Carlos Guestrin. "Xgboost: A scalable tree boosting system." *Proceedings of the 22nd acm sigkdd international conference on knowledge discovery and data mining*. 2016. |
| Min_child_weight | 1 | Minimum sum of instance weight (hessian) needed in a child. Controls overfitting by requiring a minimum weight to create a new node. | Chen, Tianqi, and Carlos Guestrin. "Xgboost: A scalable tree boosting system." *Proceedings of the 22nd acm sigkdd international conference on knowledge discovery and data mining*. 2016. |
| α (Alpha) | 0 | The L1 regularization term. A value of 0 indicates no L1 regularization. Higher values impose more regularization to reduce overfitting. | Chen, Tianqi, and Carlos Guestrin. "Xgboost: A scalable tree boosting system." *Proceedings of the 22nd acm sigkdd international conference on knowledge discovery and data mining*. 2016. |
| λ (Lambda) | 1 | The L2 regularization term. A higher value penalizes large weights, reducing model complexity and preventing overfitting. | Chen, Tianqi, and Carlos Guestrin. "Xgboost: A scalable tree boosting system." *Proceedings of the 22nd acm sigkdd international conference on knowledge discovery and data mining*. 2016. |
| Learning_rate | 0.3 | The step size used during training to update model parameters. A smaller value improves model generalization but requires more iterations. | Chen, Tianqi, and Carlos Guestrin. "Xgboost: A scalable tree boosting system." *Proceedings of the 22nd acm sigkdd international conference on knowledge discovery and data mining*. 2016. |
| Iterations | 100 | The number of boosting rounds (iterations) the model will go through. More iterations generally improve model performance, but may increase overfitting risk. | Chen, Tianqi, and Carlos Guestrin. "Xgboost: A scalable tree boosting system." *Proceedings of the 22nd acm sigkdd international conference on knowledge discovery and data mining*. 2016. |

AUC: Area Under receiver operating characteristic Curve, LBTRBC-M: Load-Bearing Tissue Radiomic plus Biochemical biomarker and Clinical variable Model.
