# Supplementary material for: Predicting knee osteoarthritis progression using neural network with longitudinal MRI radiomics, and biochemical biomarkers: A modeling study
Source: PLoS Med. 2025 Aug 21;22(8):e1004665. doi: 10.1371/journal.pmed.1004665 (PMC12370028; doi:10.1371/journal.pmed.1004665)
Supplement: S17 Table — The predictive performance of LBTRBC-M using different hyperparameters in the total test cohort. (DOCX) [file pmed.1004665.s033.docx]

**Table S17. The predictive performance of LBTRBC**-**M using different hyperparameters in the total test cohort.**

| **Model** | **Max_depth** | **Subsample** | **Colsample_bytree** | **Min_child_weight** | **α** | **λ** | **Learning_rate** | **Iterations** | **ERS** | **Accuracy** | **AUC** | ***p* value*** |
| --- | --- | --- | --- | --- | --- | --- | --- | --- | --- | --- | --- | --- |
| Optimal setting | 6 | 1 | 1 | 1 | 0 | 1 | 0.300 | 100 | 0.409 | 0.701 | 0.897 | - |
| Reference setting 1 | 5 | 0.893 | 0.361 | 6.626 | 1.893 | 1.298 | 0.067 | 108 | 0.267 | 0.622 | 0.855 | 0.356 |
| Reference setting 2 | 7 | 0.901 | 0.562 | 4.450 | 0.805 | 1.511 | 0.253 | 72 | 0.359 | 0.674 | 0.871 | 0.788 |
| Reference setting 3 | 5 | 0.614 | 0.392 | 5.734 | 1.333 | 1.955 | 0.053 | 177 | 0.268 | 0.614 | 0.846 | 0.152 |
| Reference setting 4 | 3 | 0.651 | 0.315 | 3.964 | 0.307 | 1.737 | 0.088 | 22 | 0.094 | 0.489 | 0.753 | <0.001 |
| Reference setting 5 | 4 | 0.907 | 0.310 | 3.326 | 1.726 | 0.756 | 0.290 | 241 | 0.344 | 0.640 | 0.864 | 0.564 |
| Reference setting 6 | 4 | 0.338 | 0.410 | 3.430 | 0.124 | 1.579 | 0.240 | 275 | 0.240 | 0.592 | 0.820 | 0.018 |
| Reference setting 7 | 6 | 0.407 | 0.432 | 9.189 | 1.801 | 1.055 | 0.260 | 179 | 0.247 | 0.590 | 0.810 | <0.001 |
| Reference setting 8 | 6 | 0.617 | 0.956 | 2.577 | 0.247 | 0.825 | 0.216 | 224 | 0.312 | 0.623 | 0.851 | 0.376 |
| Reference setting 9 | 3 | 0.601 | 0.935 | 9.231 | 0.701 | 1.652 | 0.142 | 252 | 0.310 | 0.623 | 0.847 | 0.141 |
| Reference setting 10 | 3 | 0.791 | 0.520 | 6.939 | 0.021 | 0.238 | 0.276 | 263 | 0.328 | 0.648 | 0.859 | 0.294 |
| Reference setting 11 | 8 | 0.643 | 0.642 | 7.912 | 0.520 | 0.262 | 0.234 | 103 | 0.322 | 0.642 | 0.851 | 0.397 |
| Reference setting 12 | 7 | 0.991 | 0.529 | 9.938 | 0.353 | 0.749 | 0.124 | 298 | 0.390 | 0.676 | 0.885 | 0.891 |
| Reference setting 13 | 8 | 0.371 | 0.684 | 6.943 | 1.387 | 1.119 | 0.181 | 294 | 0.252 | 0.582 | 0.814 | <0.001 |
| Reference setting 14 | 8 | 0.669 | 0.563 | 1.436 | 1.979 | 1.809 | 0.199 | 281 | 0.326 | 0.646 | 0.855 | 0.332 |
| Reference setting 15 | 8 | 0.514 | 0.668 | 1.096 | 0.898 | 0.115 | 0.097 | 232 | 0.348 | 0.656 | 0.868 | 0.513 |
| Reference setting 16 | 8 | 0.325 | 0.742 | 4.189 | 0.935 | 0.532 | 0.296 | 143 | 0.181 | 0.550 | 0.785 | <0.001 |
| Reference setting 17 | 7 | 0.841 | 0.623 | 3.489 | 1.566 | 0.016 | 0.163 | 157 | 0.363 | 0.658 | 0.878 | 0.870 |
| Reference setting 18 | 6 | 0.349 | 0.915 | 9.929 | 1.848 | 1.861 | 0.170 | 95 | 0.219 | 0.562 | 0.796 | <0.001 |
| Reference setting 19 | 4 | 0.452 | 0.943 | 2.902 | 1.328 | 0.080 | 0.090 | 117 | 0.283 | 0.623 | 0.843 | 0.214 |
| Reference setting 20 | 7 | 0.956 | 0.860 | 9.798 | 1.125 | 1.176 | 0.095 | 21 | 0.172 | 0.586 | 0.827 | 0.023 |

*****Delong test were performed between the optimal and reference settings to compare the AUC difference.

ERS: Entropy R Square, AUC: Area Under receiver operating characteristic Curve, LBTRBC-M: Load-Bearing Tissue Radiomic plus Biochemical Biomarker and Clinical Variable Model.
