# Supplementary material for: Predicting knee osteoarthritis progression using neural network with longitudinal MRI radiomics, and biochemical biomarkers: A modeling study
Source: PLoS Med. 2025 Aug 21;22(8):e1004665. doi: 10.1371/journal.pmed.1004665 (PMC12370028; doi:10.1371/journal.pmed.1004665)
Supplement: S18 Table — Compares the predictive performance of the LBTRBC-M model with different interactions in the total test cohort. (DOCX) [file pmed.1004665.s034.docx]

**Table S18. Compares the predictive performance of the LBTRBC**-**M model with different interantions in the total test cohort.**

|  | **JSN and pain progression** | | |  | **JSN progression** | | |  | **Pain progression** | | |  | **Non progression** | | |  |  |
| --- | --- | --- | --- | --- | --- | --- | --- | --- | --- | --- | --- | --- | --- | --- | --- | --- | --- |
| **Interantions** | **AUC (95% CI)** | **ΔAUC (95% CI)** | ***p* value** |  | **AUC (95% CI)** | **ΔAUC (95% CI)** | ***p* value** |  | **AUC (95% CI)** | **ΔAUC (95% CI)** | ***p* value** |  | **AUC (95% CI)** | **ΔAUC (95% CI)** | ***p* value** |  | **Time consumption** |
| 100 | 0.880 (0.853, 0.903) | - | - |  | 0.913 (0.881, 0.937) | - | - |  | 0.886 (0.856, 0.910) | - | - |  | 0.909 (0.888, 0.926) | - | - |  | 6 seconds |
| 300 | 0.881 (0.854, 0.903) | -0.001 (-0.005, 0.004) | 0.808 |  | 0.914 (0.882, 0.937) | -0.001 (-0.006, 0.004) | 0.765 |  | 0.878 (0.848, 0.903) | 0.008 (0.002, 0.013) | 0.011 |  | 0.907 (0.885, 0.924) | 0.002 (-0.002, 0.006) | 0.235 |  | 23 seconds |
| 1000 | 0.876 (0.849, 0.898) | 0.004 (-0.003, 0.012) | 0.219 |  | 0.911 (0.880, 0.934) | 0.002 (-0.006, 0.011) | 0.597 |  | 0.873 (0.842, 0.898) | 0.013 (0.005, 0.022) | 0.003 |  | 0.904 (0.881, 0.922) | 0.005 (-0.001, 0.012) | 0.111 |  | 104 seconds |
| 25000 | 0.868 (0.841, 0.892) | 0.012 (0.002, 0.021) | 0.015 |  | 0.909 (0.879, 0.932) | 0.004 (-0.006, 0.015) | 0.426 |  | 0.871 (0.839, 0.897) | 0.015 (0.004, 0.026) | 0.006 |  | 0.902 (0.879, 0.921) | 0.007 (-0.002, 0.016) | 0.114 |  | 15152 seconds |

AUC: Areas Under receiver operating characteristic Curve, CI: Confidence Interval, LBTRBC-M: Load-Bearing Tissue Radiomic plus Biochemical biomarker and Clinical variable Model.
