# Supplementary material for: Predicting knee osteoarthritis progression using neural network with longitudinal MRI radiomics, and biochemical biomarkers: A modeling study
Source: PLoS Med. 2025 Aug 21;22(8):e1004665. doi: 10.1371/journal.pmed.1004665 (PMC12370028; doi:10.1371/journal.pmed.1004665)
Supplement: S19 Table — Compares the predictive performance of the LBTRBC-M model using/without using the stratified cross-validation in the total test cohort. (DOCX) [file pmed.1004665.s035.docx]

**Table S19. Compares the predictive performance of the LBTRBC**-**M model using/without using the stratified cross**-**validation in the total test cohort.**

|  | **Un**-**stratified cross**-**validation** |  | **Stratified cross**-**validation** |  |  |  |
| --- | --- | --- | --- | --- | --- | --- |
| **Predicting outcomes** | **AUC (95% CI)** |  | **AUC (95% CI)** |  | **ΔAUC (95% CI)** | ***p* value** |
| JSN and pain progression | 0.880 (0.853, 0.903) |  | 0.853 (0.826, 0.877) |  | 0.027 (-0.003, 0.058) | 0.080 |
| JSN progression | 0.913 (0.881, 0.937) |  | 0.860 (0.823, 0.891) |  | 0.053 (0.016, 0.090) | 0.005 |
| Pain progression | 0.886 (0.856, 0.910) |  | 0.878 (0.843, 0.906) |  | 0.008 (-0.031, 0.047) | 0.697 |
| Non progression | 0.909 (0.888, 0.9260 |  | 0.853 (0.824, 0.877) |  | 0.056 (0.030, 0.082) | <0.001 |

AUC: Areas Under receiver operating characteristic Curve, CI: Confidence Interval, LBTRBC-M: Load-Bearing Tissue Radiomic plus Biochemical biomarker and Clinical variable Model.
