# Supplementary material for: Predicting knee osteoarthritis progression using neural network with longitudinal MRI radiomics, and biochemical biomarkers: A modeling study
Source: PLoS Med. 2025 Aug 21;22(8):e1004665. doi: 10.1371/journal.pmed.1004665 (PMC12370028; doi:10.1371/journal.pmed.1004665)
Supplement: Attachment — Submitted filename: PMEDICINE-D-24-02732R1-Response to Review.docx [file pmed.1004665.s039.docx]

Dear editors and reviewers,

We sincerely thank the reviewers and the editor for their insightful comments and constructive suggestions, which have significantly improved the quality of our manuscript. We have carefully addressed each comment and provided detailed explanations and revisions where necessary.

Reviewer #1:

1. The use of various statistical tests (e.g., unpaired t-test, one-way ANOVA, χ² test, Mann-Whitney test, Kruskal-Wallis test) for comparing different groups is appropriate. However, the rationale behind selecting specific tests for continuous versus categorical variables should be explicitly stated. Clarifying why a parametric or non-parametric test was chosen, based on the data distribution, would enhance the transparency of the methodology.

**Response: We appreciate the reviewer’s comment and have now clarified the rationale for selecting specific statistical tests in the revised manuscript. Specifically:**

**(1) For continuous variables, parametric tests (e.g., unpaired t-test, one-way ANOVA) were employed when data were normally distributed, as assessed using the Shapiro-Wilk test. Non-parametric tests (e.g., Mann-Whitney test, Kruskal-Wallis test) were used when the assumption of normality was not met.**

**(2) For categorical variables, χ² tests were used to compare proportions across groups.**

**These methodological decisions have been included in the "Statistical Analysis" section of the manuscript to enhance transparency and reproducibility. (Page 16, line 346-353)**

2. The use of Generalized Estimating Equations (GEE) for analyzing correlated data, such as repeated measures, is appropriate. However, more details should be provided on the correlation structure assumed in the GEE model. Additionally, it would be beneficial to specify how the model handles missing data and whether any sensitivity analyses were conducted to assess the robustness of the findings.

**Response: We thank the reviewer for this insightful comment and have added further details regarding the GEE model in the revised manuscript. Specifically:**

**(1) The GEE model assumed an** **autoregressive correlation structure, as this was deemed most suitable for the nature of the repeated measures in our data. This choice has been justified in the revised "Statistical Analysis" section. (Page 16, line 338-340)**

**(2)** **Missing data were addressed using multiple imputation (Predictive mean matching). Specifically, data were missing for 2 knees in the MOAKS femur lateral posterior bone marrow lesion percentage (lesion that is edema), 13 knees for serum biomarkers, and 10 knees for urine biomarkers. The percentages of missing data were 0.3%, 2.1%, and 1.7%, respectively. Given these low percentages, the impact on the results was deemed minimal. This information has been detailed in the corresponding section of the manuscript. (Page 15-16, line 328-333)**

**(3) To evaluate the robustness of our findings, sensitivity analyses were performed by re-running the GEE model using alternative correlation structures, including independent, exchangeable, and unstructured. The results were compared across these assumptions, and the analyses confirmed that our findings remained consistent and robust (Table S13). (Page 21, line 454-458)**

3. The application of LASSO for feature selection is valid, but further details could be provided on how the optimal penalty parameter (lambda) was chosen.

**Response: We thank the reviewer for highlighting the need for more details on how the optimal penalty parameter (λ) was selected in our LASSO implementation.**

**In our analysis, the selection of the optimal λ was guided by the adaptive weighting approach combined with the Akaike Information Criterion, corrected (AICc) validation method. Specifically, λ was chosen from the optimal solution path where the AICc value was minimized, ensuring the best balance between model fit and complexity.**

**This process is now described in detail in the " Three-dimensional radiomic feature analysis" section of the revised manuscript. (Page 12-13, line 263-272)**

4. 10-fold cross-validation method used to validate model performance is appropriate. However, the manuscript should describe whether stratified cross-validation was applied, particularly when dealing with imbalanced datasets, as this could significantly impact the performance metrics.

**Response: We thank the reviewer for this insightful comment. In our analysis, we employed 10-fold cross-validation to validate model performance but did not implement stratified cross-validation. We acknowledge that this may have introduced variability in class distributions across folds, potentially impacting the reliability of performance metrics such as sensitivity and specificity. This limitation has been addressed in the "Discussion" section of the revised manuscript, where we propose incorporating stratified cross-validation in future studies to ensure more balanced class distributions and enhance the robustness of model evaluation.**

**In this study, 10-fold cross-validation was used to validate model performance; however, stratified cross-validation was not implemented. This omission may have caused variability in the class distributions across folds, potentially influencing the reliability of performance metrics, particularly sensitivity and specificity. For instance, models trained on folds with disproportionately few minority class cases may underperform when applied to a more representative dataset. While the reported results showed consistency across folds, stratified cross-validation could have mitigated these potential biases. Future studies will address this limitation by incorporating stratification to ensure balanced class distributions and provide a more robust evaluation framework.** **(Page 27, lines 583-592)**

5. Using LASSO regression and cross-validation for both feature selection and model validation carries a risk of overfitting, especially if the same data is used for both processes. The authors should clarify how they mitigated feature selection bias, potentially through techniques like nested cross-validation or using a separate validation set.

**Response: We thank the reviewer for highlighting this critical point. To mitigate the risk of overfitting and feature selection bias, we employed the following strategies:**

**(1) The data were split into independent training and testing sets at the outset. Feature selection using LASSO regression was performed exclusively on the training set, ensuring that the testing data remained completely unseen during model development. (Page 11, lines 234–237)**

**(2) Within the training set, cross-validation was used during the LASSO procedure to optimize the penalty parameter (λ) and select features, minimizing the risk of overfitting to a specific subset of the data. (Page 12-13, line 263-272; page 13-14, 287-290)**

**(3) The performance of the final model was evaluated on the held-out testing set to assess its generalizability. This approach provides an unbiased estimate of the model's performance. (Page 14, lines 292–297)**

**While nested cross-validation was not explicitly employed in this study, our methodology ensured the separation of feature selection and model validation to reduce bias. We have clarified these details in the "Methods" section (Page 11, lines 234–237; page 12-13, line 263-272; page 13-14, 287-290; page 14, lines 292–297) and acknowledged the limitation of not using nested cross-validation in the "Discussion" section of the revised manuscript (Page 29-30, lines 639–641).** **We also propose incorporating nested cross-validation in future studies to further enhance methodological rigor.**

6. The manuscript mentions multiple tests, but it lacks a clear indication of how multiple comparisons were managed to control for Type I error. If multiple comparisons were indeed made, methods such as Bonferroni correction or false discovery rate (FDR) control should be discussed to ensure the robustness of the findings.

**Response: We thank the reviewer for raising this important point.** **To control for Type I error in our analysis, both Tukey’s honestly significant difference (HSD) test and Bonferroni correction were applied during one-way ANOVA tests for multiple comparisons. These methods provided consistent results, confirming the robustness of our findings.**

**We have revised the "Statistical Analysis" section of the manuscript to explicitly mention the use of these methods and their role in addressing the potential for inflated Type I error (Page 16, lines 351–353). By employing two widely recognized correction techniques, we ensured the reliability of our conclusions.**

7. The ROC analysis and DeLong test are standard methods for comparing the performance of predictive models. The authors should ensure that the interpretation of AUC, sensitivity, specificity, and kappa values is clear and tied to the clinical relevance of the findings. Including confidence intervals for the AUC values would also provide a sense of the precision of these estimates.

**Response: Thank you for the suggestion. In our revised manuscript, we ensured the following:**

**(1) We have clarified the interpretation of AUC, sensitivity, specificity, and kappa values, directly linking these metrics to the clinical relevance of predicting KOA progression. (Table S16)**

**(2) We have included 95% confidence intervals for the AUC values to provide a clearer understanding of the precision of these estimates. (Table 2, Table S6, Table S8, Table S14)**

**(3) We have ensured that the results of the DeLong test are clearly described to support the comparison of model performance. (Page 22, line 465-468)**

8. It is essential to check and report whether the assumptions underlying each statistical model were verified. For example, for LASSO regression, assumptions related to the distribution of errors and the linearity of relationships should be considered. Similarly, for GEE models, the choice of correlation structure (e.g., exchangeable, autoregressive) should be justified based on the data characteristics.

**Response: We thank the reviewer for this insightful comment and have revised the manuscript to address these concerns:**

**The assumptions underlying LASSO regression, including the distribution of errors and the linearity of relationships between predictors and the outcome, were assessed during model development. The linearity assumption was checked through exploratory data analysis and transformations applied to variables as necessary. Residual diagnostics were performed to confirm that the error terms were approximately normally distributed. These details have now been explicitly described in the "** **Three-dimensional radiomic feature analysis" section. (Page 13, lines 267–272)**

**The choice of the correlation structure for the GEE models was based on the data characteristics. An alternative correlation structure was selected, as it was deemed appropriate given the repeated measures nature of the data and the expectation of consistent correlations among observations within clusters. Sensitivity analyses were performed using alternative correlation structures to confirm the robustness of the findings. These justifications and results are now detailed in the "** **Sensitivity Analyses" section. (Page 21, lines 454-458; Table S13)**

9. While cross-validation was employed, it would be beneficial to discuss any plans or considerations for external validation. If external datasets are available, they should be used to validate the model's performance, as external validation is crucial for assessing the generalizability of predictive models.

**Response: We thank the reviewer for emphasizing the importance of external validation to assess the generalizability of predictive models. While external validation was not performed in the current study due to the unavailability of suitable external datasets at this stage, we acknowledge its importance as a critical next step.**

**We have revised the "Discussion" section of the manuscript to explicitly address this limitation and outline plans for future validation efforts.** **Specifically, we plan to test the model on independent datasets from other cohorts or institutions, such as Multicenter Osteoarthritis Study (MOST) cohort, which would provide a robust assessment of its generalizability across different populations and settings. (Page 29, lines 621-624)**

10. While the manuscript briefly mentions the handling of missing data, a more detailed discussion is warranted. The authors should specify how missing data were handled (e.g., imputation methods, exclusion) and whether any sensitivity analyses were performed to assess the impact of missing data on the study's findings.

**Response: We appreciate the reviewer’s comment and agree that a more comprehensive discussion of the handling of missing data is important for the transparency and robustness of our analysis. In response, we have made the following updates to the manuscript:**

**(1) Handling of Missing Data: Missing data were handled using multiple imputation, a method that helps address missingness without losing valuable information. Specifically:**

**multiple imputation using “****predictive mean matching” was used to impute missing values for variables with incomplete data, ensuring that the underlying relationships between variables were preserved. The variables with missing data included: 2 knees in the MOAKS femur lateral posterior bone marrow lesion percentage (Lesion that is edema), 13 knees for serum biomarkers, and 10 knees for urine biomarkers. The percentage of missing data for these variables was 0.3%, 2.1%, and 1.7%, respectively. Given these low percentages, we deemed the impact of missing data to be minimal and unlikely to significantly affect the results. (Page 15-16, line 328-333)**

**(2)** **Sensitivity Analyses: To assess the impact of missing data on the study’s findings, sensitivity analyses were conducted (Table S14). These included:** **Comparing the results from the multiple imputation approach with those obtained from a complete case analysis (excluding cases with missing data). (Page 21, line 458-463)**

**The results were consistent across both approaches, confirming that the handling of missing data did not significantly alter the study’s conclusions.**

**These updates have been incorporated into the "Methods" section of the manuscript (Page 15-16, line 328-333), and the results of the sensitivity analyses are summarized in the "Results" section (Page 21, line 458-463; Table S14).**

11. The methods section should discuss how potential confounders were addressed in the statistical models. For instance, were any variables included to adjust for confounding? If so, how were these variables selected?

**Response: Thank you for your insightful comment. In response, we have clarified the methodology and included more detailed information regarding the statistical modeling in the revised manuscript. Specifically, in Table S9 ("Related risks of outcomes for predictive model outputs"), we used GEE to assess the significance levels of individual risk factors.**

**The following points clarify the approach used in the analysis:**

**(1) Independent and Dependent Variables:**

**The outputs of the predictive models were treated as independent variables. KOA progression was treated as the dependent variable, with non-progression serving as the reference outcome.**

**(2) Adjustment for Potential Confounders: To control for potential confounders, we included baseline variables such as:** **Age, Sex, Body Mass Index (BMI), Knee side (left or right), Race, WOMAC knee pain score (as a measure of baseline knee pain).**

**These confounders were selected based on prior literature and their potential impact on KOA progression (PMID: 28212675, PMID: 29408279, PMID: 34391392, PMID: 26614536).**

**(3) Methodology: The GEE model was used to estimate the effects of the independent variables on KOA progression,** **adjusting for the confounders listed above. This approach allows for robust estimation of standard errors in the presence of correlated data from repeated measurements.**

**These details have been added to the "** **Statistical Analysis" sections of the manuscript for greater clarity and transparency.** **(Page 16, lines 335–338)**

12. The manuscript does not mention whether interaction terms were considered in the models. Given the complexity of the relationships between radiomic features, biomarkers, and clinical variables, it might be worthwhile to explore interaction terms to capture any synergistic effects. If interactions were tested but found not to be significant, this should be clearly stated.

**Response: We appreciate the reviewer’s suggestion regarding the consideration of interaction terms. In response, we have reviewed the inclusion of interaction terms in our models:**

**(1) Consideration of Interaction Terms: Given the complexity of the relationships between radiomic features, biomarkers, and clinical variables, we initially considered including interaction terms in the models to explore potential synergistic effects between these variables.**

**(2)** **Testing of Interactions: Interaction terms were tested for combinations of key predictors (e.g., radiomic features with clinical variables or biomarkers). We tested the significance of interaction terms by comparing the AUC differences between different model combinations (LBTRBC-M vs. LBTRB-M, LBTRBC-M vs. LBTRC-M, LBTRC-M vs. LBTRC-M). These comparisons were performed using the DeLong test to assess the statistical significance of the AUC differences. The results of these comparisons are shown in Table S8, which includes data from test cohorts 1, 2, 3, and the total test cohort. We found that there were no significant differences in AUC between the models in any of the cohorts, indicating that the addition of different features (e.g., biochemical biomarkers, clinical variables) did not significantly improve the model’s predictive performance when compared with other combinations.**

**We have now included a statement in the "Results" sections clarifying that interaction terms were tested, but none of them were found to be significant (Page 22, lines 471-476). This clarification ensures that the complexity of the relationships between the variables is addressed while maintaining transparency regarding the lack of significant interactions.**

13. If there are subgroups within the data (e.g., based on demographic variables like age or sex), the authors might consider stratified analysis or clustering techniques to explore whether the model performs differently across these subgroups.

**Response: We thank the reviewer for this thoughtful suggestion. In response, we considered stratified analysis or clustering techniques to assess whether the model's performance varied across different subgroups, such as age or sex. However, due to the sample size and the complexity of the models, we did not perform these subgroup analyses in the current study.**

**Nonetheless, we acknowledge that exploring model performance across subgroups could provide valuable insights, particularly in understanding how demographic variables might influence the predictive power of the models. We have included this as a suggestion for future work in the Discussion section of the manuscript, where we outline plans to explore stratified analyses and clustering in larger, more diverse cohorts to better understand subgroup-specific model performance. (Page 26-27, lines 574–582)**

14. While LASSO and XGBOOST can handle some non-linearity, it might be worth exploring whether more explicitly non-linear modeling approaches (e.g., spline regression, generalized additive models) could provide better fits for certain variables, especially those that exhibit complex relationships with the outcome.

**Response: We appreciate the reviewer’s insightful suggestion. In this study, we employed LASSO and XGBOOST, both of which can capture non-linear relationships to some extent. However, we acknowledge that more explicitly non-linear approaches, such as spline regression or generalized additive models (GAMs), could provide a better fit for variables with complex relationships to the outcome.**

**While these approaches were not explored in the current study due to the focus on comparing traditional and machine learning methods, we recognize their potential to improve model accuracy for certain predictors. We have added a discussion in the Discussion section outlining this as an area for future research. Specifically, we plan to explore GAMs or spline-based methods in future studies to better capture non-linear effects, especially for variables that exhibit complex interactions with the outcome. (Page 29, lines 636–637)**

15. Given that XGBOOST and LASSO involve the selection of hyperparameters, the manuscript should discuss how sensitive the results are to different hyperparameter choices. Grid search or Bayesian optimization methods for hyperparameter tuning could be mentioned to ensure that the model is optimally configured.

**Response: We thank the reviewer for raising this important point regarding hyperparameter tuning. In our study, hyperparameters for both XGBOOST and LASSO were carefully selected to optimize model performance. Below, we provide details on the hyperparameter choices and tuning methods:**

**(1) XGBOOST Hyperparameters: The following hyperparameters were selected for XGBOOST: max_depth: 6, subsample: 1, colsample_bytree: 1, min_child_weight: 1, alpha: 0, λ: 1, learning_rate: 0.3, iterations: 100. (Page 13, lines 281-285)**

**These hyperparameters were chosen based on prior literature and initial experiments (Hastie, Trevor,, et al. The Elements of Statistical Learning: Data Mining, Inference, and Prediction. 2nd ed. New York, Springer, 2009.). We optimized the model using grid search with cross-validation to find the best combination of these parameters. (Page 13, line 286-287)**

**(2) LASSO Hyperparameters: For LASSO, the penalty parameter (λ) was optimized using cross-validation, and the value that minimized the mean squared error (MSE) was selected. (Page 13-14, lines 287-289)**

**(3) Model Sensitivity: We acknowledge that the model performance can be sensitive to the choice of hyperparameters. However, our grid search for XGBOOST involved testing a range of values, and we observed stable performance across different parameter configurations. This was confirmed by the minimal impact of hyperparameter changes on the model outcomes. (Page 21-22, lines 463-468; Table S17)**

**(4) Advanced Hyperparameter Tuning: We used an adaptive grid search with a total of** **150 grid points for XGBOOST. The minimum penalty fraction was set to 0.001, and the grid scale was set to the square root. The initial solution was selected based on the best fit approach. This method provided an efficient way of exploring the parameter space and improving model accuracy. (Page 13, lines 286-287)**

**(5) Future Work: In future studies, we plan to explore Bayesian optimization or other advanced techniques to refine hyperparameter tuning further, enhancing the model’s performance and exploring the parameter space more effectively.**

**These details have been added to the "Methods" section of the manuscript to ensure transparency and clarity in the hyperparameter tuning process. (Page 13-14, lines 281–290)**

16. While XGBOOST is a powerful algorithm, the authors might consider discussing why this specific method was chosen over other machine learning techniques (e.g., Random Forest, Support Vector Machines, Neural Networks). A brief comparison or justification could strengthen the manuscript's methodological rigor.

**Response: We appreciate the reviewer’s comment and have expanded on the discussion regarding our choice of algorithm. In our study, we compared the LBTRBC-M model using various machine learning algorithms to assess their predictive performance in the total test cohort. The performance metrics are shown in Table S15, which includes XGBOOST, Bootstrap Forest, Shallow Neural Network, Support Vector Machines, Decision Tree, Nominal Logistic, and Naive Bayes.**

**The results indicate that XGBOOST outperforms the other algorithms across all key metrics:**

**(****1) Entropy R square: XGBOOST achieved 0.409, showing superior model fit compared to other algorithms, especially Naive Bayes (-5.538).**

**(2) Misclassification rate: XGBOOST exhibited the second-lowest misclassification rate (0.299), slightly behind Bootstrap Forest (0.179), indicating its strong ability to correctly classify the test samples.**

**(3) AUC: XGBOOST had the highest AUC of 0.897, surpassing all other models, including Bootstrap Forest (0.833) and Shallow Neural Network (0.807). This demonstrates its excellent discriminative ability.**

**(4)** **Root average squared error: XGBOOST achieved the lowest root average squared error (0.508), further highlighting its precision in minimizing prediction error, in contrast to models like Support Vector Machines (0.691) and Naive Bayes (0.792).**

**Given these findings, XGBOOST is considered the optimal algorithm for our LBTRBC-M model in this study, offering the best overall performance in terms of entropy R square, misclassification rate, AUC and root average squared error. These results are now included in the manuscript for greater transparency in our methodological choices. (Page 18, lines 386–395; Table S15)**

17. While the manuscript discusses techniques like cross-validation, it would be prudent to further elaborate on how the risk of overfitting was mitigated, particularly given the complexity of models like XGBOOST. The discussion could include the importance of balancing model complexity with simplicity in design.

**Response: We appreciate the reviewer’s comment and agree that it is important to further address the risk of overfitting, especially when using complex models like XGBOOST. In our study, we employed several strategies to mitigate overfitting and ensure that the models were both accurate and generalizable.**

**In this study, we took several steps to mitigate the risk of overfitting, particularly given the complexity of the XGBOOST model. First, we employed 10-fold cross-validation to ensure that the model's performance was consistently evaluated across different subsets of the data, providing a robust estimate of its ability to generalize to unseen data. To further prevent overfitting, hyperparameters for XGBOOST were optimized through a grid search with cross-validation, carefully balancing model complexity and performance. Additionally, we used L1 and L2 regularization (via the alpha and λ parameters) to penalize overly complex models and reduce the risk of fitting noise in the data. We also explored simpler models, such as Logistic Regression and Decision Trees, to ensure that the performance improvements achieved with XGBOOST were not the result of overfitting but were meaningful and reflective of true model improvement. These strategies helped ensure that our models were both accurate and generalizable, providing reliable predictions for KOA progression.**

**These methods have been discussed in the revised Discussion section of the manuscript to clarify how overfitting was mitigated and how we balanced model complexity with interpretability and generalizability. (Page 27-28, lines 593–599)**

18. Authors could include robustness checks to test the stability of their findings under different assumptions or subsets of data. For example, re-running analyses after excluding outliers, using different modeling techniques, or testing the model on various subsets of data could provide additional confidence in the results.

**Response: We thank the reviewer for their insightful suggestion regarding the inclusion of robustness checks. We acknowledge that conducting such checks would strengthen the validity and generalizability of our findings. However, the current manuscript does not include these additional analyses, which can be considered a limitation of the study. Specifically:**

**(1) While outliers were not explicitly excluded from our analysis, their potential impact on model performance was not assessed. In future studies, we plan to conduct robustness checks by re-running the analysis after excluding or mitigating outliers. This would help evaluate whether extreme values influence the model results. (Page 29, lines 633–636)**

**(2) Although we focused on XGBOOST and other commonly used machine learning models, exploring additional techniques (e.g., Generalized Additive Models (GAMs) or Spline Regression) was not done in this study. Future work will compare results across multiple modeling approaches to confirm the stability of the findings. (Page 29, lines 636–637)**

**(3) Our study did not explore the model's stability across different subsets of the data, such as stratifying by age, sex, or other demographic factors. Testing the model on diverse data subsets in future analyses will help determine whether the findings hold across different population groups. (Page 29, lines 638–639)**

**We have acknowledged these limitations in the Discussion section of the manuscript and outlined our plans to address them in future studies. (Page 29, lines 633–639)**

19. Given the complexity of the models, the authors should discuss how uncertainty in the model predictions is quantified and reported. This could include confidence intervals, prediction intervals, or Bayesian approaches that provide a probabilistic interpretation of model outputs.

**Response: We acknowledge the importance of quantifying and reporting uncertainty in our model predictions. In our study, we addressed uncertainty by incorporating confidence intervals for key performance metrics, such as AUC, sensitivity, and specificity, providing a measure of precision (Table 2, Table S6, Table S8, Table S14). While prediction intervals and Bayesian approaches were not employed in this analysis, we recognize their value in offering a probabilistic interpretation of model outputs. Future work will explore these advanced methods to better capture and communicate the uncertainty in predictions, ensuring greater reliability and transparency in model outcomes.**

20. The manuscript could benefit from a discussion on scenario analysis, where the model's predictions are tested under different hypothetical scenarios. This approach could help in understanding how changes in key variables (e.g., a sudden change in patient demographics or the introduction of a new treatment) might impact predictions.

**Response: We appreciate the suggestion to include a discussion on scenario analysis. This is indeed a valuable approach to understanding the robustness of the model and its predictions under varying conditions. For example, we could test the model's predictions by simulating changes in key variables, such as shifts in patient demographics (e.g., age or BMI distributions) or the introduction of new treatments that could alter disease progression patterns. In future studies, we plan to implement scenario analyses to evaluate how these hypothetical changes might impact model outcomes, thereby providing a more comprehensive understanding of the model's applicability and robustness in real-world settings. (Page 30, lines 641–643)**

21. While the manuscript compares the proposed model with other models, a more detailed comparative analysis with traditional statistical methods or simpler machine learning models (e.g., logistic regression, decision trees) could provide insights into the added value of the more complex modeling approaches used.

**Response: We thank the reviewer for this valuable suggestion. We agree that a more detailed comparison with traditional statistical methods and simpler machine learning models could provide important insights into the added value of the more complex models we employed, such as XGBOOST.**

**In response to this feedback, we have expanded the comparative analysis to include logistic regression and decision trees (Table S15). These simpler models were tested alongside our proposed LBTRBC-M model, which integrates radiomic features, biochemical biomarkers, and clinical variables. We found that while XGBOOST outperformed both logistic regression and decision trees in terms of predictive accuracy (as measured by entropy R square, misclassification rate, AUC, root average squared error), the simpler models provided useful benchmarks for understanding the incremental benefits of incorporating more complex interactions in the data.**

**We have now included a more detailed discussion of these comparisons in the Results sections. (Page 18, lines 386-395; Table S15)**

22. The manuscript discusses sensitivity and specificity, but it would be valuable to delve deeper into the trade-offs between these metrics. For instance, depending on the clinical application, different thresholds might be appropriate, and ROC curves or precision-recall curves could be used to explore these trade-offs in more detail.

**Response:** **In our additional analysis of the contour plot for the LBTRAB-M model (Figure S3C), we observed distinct probability distributions for correctly predicted outcomes across different progression categories. For JSN and pain progression, probabilities were concentrated between 5.0%-99.0%; for JSN progression, they clustered between 15.0%-25.0% and 55.0%-75.0%; for pain progression, they ranged from 2.0%-25.0%; and for non-progression, they were concentrated between 50.0%-99.0%. These distributions highlight the varying probability thresholds needed for accurate predictions across categories. Exploring these thresholds through ROC and precision-recall curves could further clarify the trade-offs between sensitivity and specificity, helping tailor model thresholds to specific clinical applications for optimal predictive performance. (Page 22, lines 479-485; Figure S3C)**

23. The statistical analysis should be carefully interpreted within the context of clinical significance, not just statistical significance. The authors should avoid over-reliance on p-values and instead emphasize effect sizes and confidence intervals.

**Response: We appreciate the reviewer’s valuable comment and agree that interpreting the results within the context of clinical significance is crucial. In response to this, we have made the following revisions to the manuscript:**

**While we have reported statistical significance, we recognize that clinical relevance is of primary importance in the context of healthcare applications. In the Discussion section, we have expanded our discussion to emphasize the clinical significance of our findings, particularly in terms of their practical implications for KOA progression. We focus on the magnitude of effects and how they might influence clinical decision-making. Our predictive model shows clinical potential in managing KOA progression by integrating radiomic features, biomarkers, and clinical data. It enables personalized risk assessments, aiding clinicians in early interventions and tailored treatment plans to slow disease progression and reduce invasive procedures. The model also supports patient monitoring for timely care adjustments. While further validation in diverse cohorts is needed, it represents a promising tool for enhancing precision medicine and optimizing resource allocation in KOA management. (Page 28, lines 600-606)**

**We have placed greater emphasis on reporting effect sizes (e.g., odds ratios)(Table S9) and confidence intervals (Table 2, S6, S8, S12, S13) alongside P values. This provides a clearer picture of the strength and precision of the relationships observed, helping to contextualize the statistical findings in a clinically meaningful way. We believe that confidence intervals offer a more reliable estimate of the range within which the true effect size lies, and we now highlight this in the discussion of model performance.**

Additional comments:

24. The statistical analyses were performed using R (version 4.1.1) and SAS (version 9.4). The authors should ensure that all relevant packages (e.g., pROC in R) and their versions are cited. This is crucial for ensuring the reproducibility of the analyses.

**Response: We thank the reviewer for this important suggestion. We agree that ensuring the reproducibility of our analyses is crucial, and we appreciate the reminder to include the relevant packages and their versions.**

**In response, we have updated the Methods section to include the specific versions of R and the pROC package used in the statistical analyses. All analyses were performed using R (version 4.1.1) with the pROC package (version 1.18.0)(Page 17, lines 355-356). We have now cited this along with other relevant packages, as well as the version of SAS (version 9.4)(Page 12, lines 262), ensuring full transparency and reproducibility of our methods.**

25. It is commendable that the authors have made their data and source code publicly available. However, they should also ensure that all the scripts used for the analysis are well-documented and that a clear workflow is provided. This would facilitate others in reproducing the results.

**Response: We appreciate the reviewer’s recognition of the availability of our data and source code. We agree that clear documentation and an organized workflow are essential for ensuring reproducibility and transparency in research.**

**In response to this suggestion, we have taken the following steps:**

**(1) We have ensured that all the scripts used in the analysis are thoroughly documented. This includes detailed comments and explanations within the code to describe each step of the analysis process, including data preprocessing, model training, hyperparameter tuning, and evaluation.**

**(2) We have created a clear and structured workflow for the analysis, outlining each phase of the data analysis pipeline. This workflow includes an overview of the steps from data loading to final model evaluation, making it easier for others to follow and reproduce the analysis.**

**(3) Both the documented scripts and the workflow are available alongside the data and source code on Github (https://github.com/dmlc/xgboost). A README file has been included to provide further guidance on how to execute the scripts and reproduce the results.**

26. Ensure that all figures and their legends in the supplementary materials are self-explanatory. For example, Figure S4 and Figure S5 detail the feature selection process using LASSO regression, but the legends could benefit from a more detailed explanation of the significance of the selected features and their impact on model performance. Adding more context would help readers who may not be intimately familiar with LASSO or radiomic features.

**Response: We thank the reviewer for this helpful comment. We agree that providing more context and clarity in the figure legends will enhance the comprehensibility of the supplementary materials, particularly for readers who may not be familiar with LASSO regression or radiomic features.**

**In response, we have revised the legends for Figure S4 and Figure S5**

**Figure S4. Feature selection process by LASSO regression in single-structure radiomic models. Panel (A) to (F) show the magnitude of scaled parameter estimates for each model (FE-RM, FC-RM, TI-RM, TC-RM, LM-RM, and MM-RM), which indicate the importance of each radiomic feature in predicting KOA progression. Panels (A), (B), (C), (D), (E), and (F) represent the magnitude of scaled parameter estimates for FE-RM, FC-RM, TI-RM, TC-RM, LM-RM, and MM-RM, respectively. Panels (G) to (L) present the scaled parameter estimates of the same models using the AICc for feature selection, providing an additional measure of model performance and fit. Panels (M) to (R) illustrate the weight of features for each model, showing the relative contribution of each selected feature to the overall predictive power of the model. These results highlight the most influential features in each radiomic model for predicting KOA progression. FE-RM: Femur Radiomic Model, FC-RM: Femoral Cartilage Radiomic Model, TI-RM: Tibia Radiomic Model, TC-RM: Tibial Cartilage Radiomic Model, LM-RM: Lateral Meniscal Radiomic Model, MM-RM: Medial Meniscal Radiomic Model, AICc: Akaike Information Criterion, corrected. (Page 37-38, lines 862-877)**

**Figure S5. Feature selection process by LASSO regression in the LBT-RM and LBTRBC-M models. Panel (A) and (B) show the magnitude of scaled parameter estimates for the LBT-RM and the LBTRBC-M, respectively. Panels (C) and (D) represent the AICc-based selection of the most important features for both models, offering an alternative approach to assess the performance and fit of the models. Panels (E) and (F) show the weight of features in LBT-RM and LBTRBC-M, respectively, demonstrating how individual features contribute to the predictive power of each model. These visualizations offer a clearer understanding of the key features selected by LASSO regression and their impact on model performance for predicting KOA progression. LBT-RM: Load-Bearing Tissue Radiomic Model, LBTRBC-M: Load-Bearing Tissue Radiomic plus Biochemical Biomarker and Clinical Variable Model, AICc: Akaike Information Criterion, corrected. (Page 38-39, lines 888-899)**

27. Supplementary tables, such as Table S2 and Table S3, contain extensive data on baseline characteristics and biomarker levels. Each table should have clear annotations explaining what each biomarker or clinical variable represents, especially for an interdisciplinary audience. For instance, terms like "sCOMP" or "sHA" should be briefly described or referenced to where a description can be found.

**Response: We thank the reviewer for their insightful suggestion. To ensure clarity for an interdisciplinary audience, we have revised Table S2 and Table S3 by providing the full names for the biomarkers and clinical variables represented by abbreviations. In line with the sequence in which the abbreviations appear in the tables, we have added the full terms below each table and biological process indicate in Table S2, ensuring that terms like sCOMP (serum cartilage oligomeric matrix protein) and sHA (serum hyaluronan) are properly explained. This approach improves the accessibility of the tables and helps readers better understand the clinical and biomarker data presented. These updates have been reflected in the revised Supplementary Tables S2 and S3, which now include clear explanations of the abbreviations used.**

28. Including a workflow diagram in the supplementary materials could aid in understanding complex processes. For example, a diagram showing the steps from raw MRI data through to the final model could be beneficial, especially in understanding the MRI segmentation scheme outlined in Figure S2.

**Response: We thank the reviewer for this valuable suggestion. We agree that a workflow diagram would significantly enhance the clarity of the complex processes involved in our study, particularly in illustrating the steps from raw MRI data to the final model. In response, we have added a workflow diagram to the Figure S2. This workflow diagram is based on the automatic segmentation process described in previous studies (PMID: 30529224 and PMID: 29526784) and provides a more intuitive understanding of the entire segmentation process.**

Reviewer #2: Osteoarthritis (OA) is the most frequent musculoskeletal disease and the knee OA is the most frequent symptomatic site involved.

In this paper authors aimed to provide a new model of KOA progression prediction using both clinical, biochemical and imaging parameters based on the FNIH biomarker consortium.

This paper is relevant and contributive to the field however there are some issues which must be revised before the paper can be accepted.

Major issues:

1. It is quite unusual to see modeling processes with 25,000 repetitions for cross-validation. Typically, standard practices involve around 100 iterations, with 300 iterations being a common upper limit unless the data complexity demands more. This excessive number of iterations might lead to diminishing returns and could indicate overfitting if not carefully monitored. It therefore would be beneficial for the authors to clarify the rationale behind such a high number of repetitions. They should explain how this approach improves predictive performance and whether they observed any significant differences in outcomes compared to more conventional methods. This would help strengthen their methodology section and provide a clearer justification for their choices.

**Response: We thank the reviewer for raising this important concern. We acknowledge that performing 25,000 repetitions for cross-validation is indeed an unusually high number, and we appreciate the opportunity to clarify the rationale behind this choice.**

**The high number of repetitions was chosen to ensure the robustness and stability of the model’s performance, especially considering the complexity of our dataset and the relatively small sample size. The rationale behind using a larger number of iterations was based on the desire to better estimate the variability in model performance and avoid overfitting that might result from fewer iterations, particularly in high-dimensional spaces with many features.**

**During preliminary analyses, we observed that with fewer repetitions (e.g., 100 or 300), the model’s performance metrics showed more variability, which we believed could lead to less reliable conclusions. By increasing the number of repetitions to 25,000, we aimed to stabilize the performance estimates, ensuring more precise and consistent results.**

**While the choice of 25,000 repetitions may seem excessive, our analysis showed no evidence of diminishing returns in predictive performance with this number of iterations. We monitored the model’s performance over successive iterations, and the improvement in predictive accuracy plateaued at a certain point, suggesting that further iterations would have minimal impact on model performance.**

**We also tested the model using more conventional methods (e.g., 100 and 300 repetitions), and while the results were generally consistent, we found that the higher number of repetitions provided a slightly more stable estimate of performance. However, we did not observe significant differences in the outcomes compared to the results obtained with a more conventional number of iterations. As such, we acknowledge that 25,000 repetitions may not be necessary for every scenario, and we plan to explore fewer iterations in future studies for greater efficiency, particularly when working with larger datasets.**

**We also carefully monitored the possibility of overfitting during cross-validation by assessing the generalization error and checking performance metrics such as AUC and RASE. We did not observe signs of overfitting, and the model performed consistently across different folds and iterations, reinforcing our confidence in the validity of the results.**

**We have added a more detailed explanation of this choice to the Methods section to clarify the rationale behind using 25,000 repetitions for cross-validation and to provide greater transparency regarding its impact on model performance. (Page 12, lines 245-250)**

2. The extensive iterations used in both the cross-validation and feature selection processes lend credibility to the predictive modeling efforts in the study. However, it is crucial to address the computational feasibility and interpret the potential implications of such high iteration counts. Overall, these aspects contribute to the manuscript's strength and could be emphasized further to showcase the rigor of the research methodology.

**Response: We thank the reviewer for their insightful comment. We agree that the use of extensive iterations in cross-validation and feature selection contributes to the robustness of our model. However, we acknowledge the need to clarify the computational feasibility and interpret the implications of using such a high number of iterations.**

**To clarify, we conducted 25,000 iterations for cross-validation, a number chosen to ensure the stability and precision of the model's performance estimates. During preliminary tests, we found that fewer iterations (e.g., 100 or 300) resulted in greater variability in performance metrics, which could reduce the reliability of the results. The higher number of iterations was intended to provide more consistent and precise estimates, particularly given the high dimensionality of our dataset. This approach minimized the potential for overfitting and provided a more accurate evaluation of model performance. (Page 12, lines 245-250)**

3. Clarification on Feature Selection Process: The methodology mentions using LASSO for feature selection before applying the XGBOOST algorithm. Providing more details on the feature selection process, including criteria for inclusion or exclusion of features, would improve transparency and reproducibility.

**Response: We thank the reviewer for this helpful suggestion. We agree that providing more detail on the feature selection process will enhance the transparency and reproducibility of our methodology.**

**In response, we have expanded the Methods section to provide a more detailed explanation of the LASSO feature selection process used prior to applying the XGBOOST algorithm. Specifically, the process involved the following steps:**

**(1) We used LASSO regression as a regularization technique to select the most important features from the dataset. LASSO helps prevent overfitting by shrinking the coefficients of less important features to zero, effectively excluding them from the model.**

**(2) The selection of features was based on the magnitude of their coefficients after LASSO regularization. Features with non-zero coefficients were retained for further analysis, while those with zero coefficients were excluded. This ensured that only the most relevant features, those with the strongest predictive power, were selected for model development.**

**(3) The optimal penalty parameter λ for LASSO was selected using the Akaike Information Criterion, corrected (AICc). The value of λ that minimized the AICc was chosen, ensuring an optimal balance between model fit and complexity. This step was critical in determining the number of features included in the model.**

**(4) Prior to LASSO, all features were appropriately scaled to ensure that variables with larger ranges did not dominate the selection process. This was done to maintain the consistency and fairness of feature evaluation across different variable types.**

**We have incorporated these details into the Methods section to clarify the criteria for feature inclusion and exclusion and ensure a more transparent explanation of the LASSO process. (Page 12-13, lines 263-272)**

4. Temporal Analysis and Model Interpretation: Given the longitudinal nature of the data, discussing how changes over time influence the predictive model could be beneficial. Analyzing the temporal dynamics of specific features and their relationship with KOA progression would provide deeper insights into the disease's progression and the model's interpretability.

**Response: We appreciate the suggestion to incorporate temporal analysis into our study. Given the longitudinal nature of our data, examining how feature dynamics evolve over time and their relationship with KOA progression could provide valuable insights. Temporal trends in radiomic features, biomarkers, and clinical variables may reveal patterns that static analyses cannot capture. In future work, we aim to explore these temporal dynamics more thoroughly to enhance model interpretability and improve predictions by accounting for disease progression trajectories. (Page 30, lines 643-647)**

5. Consideration of Clinical Relevance: Discussing the clinical implications of their predictive model, including how it could be applied in real-world settings and its potential impact on patient management, would add significant value to the manuscript.

**Response: We thank the reviewer for this valuable suggestion. We agree that emphasizing the clinical relevance of our predictive model is crucial for demonstrating its potential impact on real-world applications, particularly in patient management.**

**In response, we have expanded the Discussion section to include a more detailed exploration of the clinical implications of our model. Specifically, we highlight the following points:**

**(1) We discuss how our model could be used to assist clinicians in identifying patients at high risk of KOA progression early in the disease process. By integrating radiomic features, biochemical biomarkers, and clinical variables, the model could offer more personalized predictions, enabling healthcare providers to tailor treatment strategies based on individual risk profiles. This could improve patient outcomes by facilitating early interventions.**

**(2) The model could aid in making more informed decisions about treatment options, including the choice between conservative management (e.g., physical therapy, medication) and more invasive interventions (e.g., surgery or joint replacement). By accurately predicting the progression of KOA, clinicians could prioritize interventions for patients who are more likely to experience rapid progression, potentially delaying or preventing the need for more invasive treatments.**

**(3) Our model could also be integrated into clinical workflows to help guide monitoring and follow-up. For example, patients identified as high-risk could be scheduled for more frequent monitoring, enabling clinicians to track disease progression more closely and adjust treatment plans accordingly.**

**(4) We also acknowledge potential barriers to the clinical adoption of such a model, including the need for clinical validation in larger, diverse populations. These challenges should be addressed in future studies to ensure that the model is both effective and feasible in real-world settings.**

**We believe that by highlighting these potential clinical applications, we provide a clearer understanding of the real-world value of our model and its potential to influence patient care in the management of KOA. (Page 28, lines 600-606)**

6. A more comprehensive review of the literature, citing prominent groups and contextualizing this study among both classic and recent research, would enhance the scientific rigor and relevance of the manuscript. The lack of citations from influential groups such as Saarakkala or Lespessailles raises concerns about comprehensiveness in the literature review.

**Response: We thank the reviewer for this valuable suggestion. We agree that a more comprehensive literature review is essential for providing context and enhancing the scientific rigor of the manuscript.**

**In response, we have expanded the Literature Review section to include additional references to influential studies and groups, particularly those of Saarakkala and Lespessailles, as well as other prominent researchers in the field of KOA and radiomics. Specifically:**

**(1) Saarakkala et al. have made significant contributions to understanding MRI-based biomarkers and their relationship with KOA progression. We have now included relevant studies from Saarakkala's group, particularly those related to MRI segmentation and the use of radiomic features to assess cartilage degeneration and disease progression, which align with the methodology used in our study. (E. Panfilov, S. Saarakkala, M. T. Nieminen and A. Tiulpin, "Predicting Knee Osteoarthritis Progression from Structural MRI Using Deep Learning," 2022 IEEE 19th International Symposium on Biomedical Imaging (ISBI), Kolkata, India, 2022, pp. 1-5, doi: 10.1109/ISBI52829.2022.9761458.)**

**(2) Lespessailles et al. have conducted extensive work on the use of biomarkers and clinical variables in predicting KOA outcomes. We have added citations to their work, which provides a broader perspective on the integration of clinical and biomarker data in predictive modeling, further contextualizing the significance of our approach. (PMID: 38540279)**

**These updates are reflected in the revised Introduction sections, and we believe they significantly enhance the comprehensiveness of the literature review and strengthen the manuscript’s scientific relevance. (Page 6, lines 116-123)**

Minor issues:

1. P7, second paragraph: "The main analysis focused on comparing knees with both JSN and pain progression to all other knees. (16)". I wonder if ref (16) is appropriate because it is an old reference (2014) and a position paper rather than an original article.

**Response: We appreciate the reviewer’s comment regarding the reference in question. You are correct that Reference (16) is a position paper rather than an original research article, and we recognize that the publication date is relatively old.**

**In response, we have carefully reviewed the reference and have replaced it with more recent and relevant sources (PMID: 27343253, 2017 Jan; PMID: 27296323, 2017 Jan) that better align with the objectives of our study. We have now cited original research articles that provide updated insights into JSN of KOA, and pain progression. These newer references offer a more robust foundation for the methodology we applied in our analysis and better reflect the latest developments in the field.**

**The updated reference list has been revised accordingly in the Methods section (Page 8, lines 172), and we believe this strengthens the manuscript’s scientific rigor.**

2. P8, again, (ref18) reported the effects of sprifermin is a clinical trial and thus is not appropriate.

**Response: We appreciate the reviewer’s comment and agree that Ref 18, which reports the effects of sprifermin in a clinical trial, may not be the most appropriate for this particular context. In response, we have removed Reference 18 and replaced it with more relevant literature that better supports the predictive modeling aspects of our study. (Page 8, lines 172)**

3. P9, ref (19) is the main reference to describe the KL method but do not explain in the present study how and who analyses the radiographs.

**Response: We thank the reviewer for pointing this out. We agree that further clarification is needed regarding how and who analyzed the radiographs in our study.**

**In response, we have added the following details to the Methods section to clarify the process:**

**The KLG assessments for radiographs were performed by Dr. Piran Aliabadi, MD and Dr. Burt Sack, MD, under the direction of Dr. David Felson, MD from the Boston University Clinical Epidemiology Research and Training Unit for the baseline through 24-month visits. (Page 9, lines 191-194)**

4. P18, BCM, this has not been defined previously.

**Response: We appreciate the reviewer’s comment. We apologize for the oversight. In response, we have now defined BCM (Biochemical Biomarker plus Clinical Variable Model) earlier in the manuscript. (Page 19, lines 412-413)**

5. P19, in fact, a recent paper has investigated the combination of biomechanical and radiological biomarkers to predict KOA progression in the FNIH dataset (https://doi.org/10.3390/biomedicines12030666 Performance of Radiological and Biochemical Biomarkers in Predicting Radio-Symptomatic Knee Osteoarthritis Progression- Biomedicines-2024).

**Response: We thank the reviewer for bringing this recent study to our attention. We agree that the work referenced provides valuable insights into the combination of biomechanical and radiological biomarkers for predicting KOA progression in the FNIH dataset.**

**In response, we have reviewed the article and incorporated a relevant citation to the study by Lespessailles et al. (2024) in the Introduction section** **(Page 6, lines 116-123)** **to contextualize our findings and highlight how they align with or differ from this recent work. We believe this addition strengthens the manuscript by acknowledging the latest advancements in the field and providing a broader perspective on the integration of biomechanical and radiological biomarkers in predictive modeling for KOA progression.**

6. P19, authors wrote « Similarly, combining MRI and radiographic markers resulted in an AUC of 0.718, which increased to 0.722 with the addition of biochemical biomarkers.(8) ». However moving from 0.718 to 0.722, was this numerically better AUC statistically and clinically significant?

**Response: We appreciate the reviewer’s comment and agree that it is important to assess whether the increase in AUC from 0.718 to 0.722 is both statistically and clinically significant.**

**In response, we have performed additional statistical analysis to evaluate the significance of a small increase in AUC from LBT-RM (Load-Bearing Tissue Radiomics Model) to LBTRB-M (Load-Bearing Tissue Radiomics plus Clinical variable Model). We conducted a DeLong test to compare the AUC values between the two models (LBT-RM vs. LBTRB-M). The results indicated that the increase in AUC from 0.806 to 0.862 was not statistically significant (P value >.05, Table S8). This suggests that the addition of biochemical biomarkers did not substantially improve the model's discriminative ability at a statistically significant level.**

**Regarding clinical significance, while the increase in AUC was modest, we recognize that even small improvements in AUC can have meaningful implications in the clinical context, particularly in the management of KOA progression. In the Discussion section, we acknowledge that while this small increase in AUC may not reach statistical significance, it may still reflect the added value of incorporating biochemical biomarkers into the model, especially given their potential to provide additional insights that imaging markers alone may not capture. (Page 25-26, lines 546-554)**

Reviewer #3: In this paper, the authors propose a diagnostic and prognostic model for knee osteoarthritis progression. Using data from the Osteoarthritis Initiative (OAI) and advanced modeling techniques like XGBOOST, convolutional neural networks, and longitudinal MRI radiomics, the study demonstrates the potential to improve prediction accuracy for joint space narrowing and pain progression. While this work is innovative and aligns with the journal's scope, significant revisions are necessary before publication.

-The introduction lacks a clear articulation of the study's specific goals and the gaps it aims to address.

**Response: We thank the reviewer for pointing this out. We agree that a clearer articulation of the study's specific goals and the gaps it aims to address will improve the manuscript's clarity and focus.**

**In response, we have revised the Introduction section to explicitly state the primary objectives of the study, as well as the key gaps in current research that we aim to address (Page 6, lines 116-123). Specifically, we now highlight how the study seeks to improve the prediction of KOA progression by integrating radiomic biomarkers, biochemical biomarkers, and clinical variables, a combination that has not been fully explored in previous studies. We also clarify the limitations of existing predictive models and how our approach aims to provide more personalized and accurate predictions for early intervention and treatment strategies.**

-While the background is comprehensive, it could be condensed to focus more on relevant prior studies and their shortcomings.

**Response: We appreciate the reviewer’s suggestion to condense the background section. In response, we have revised the Introduction to streamline the discussion, focusing more specifically on the key prior studies that are directly relevant to our work and clearly highlighting their limitations. This allows us to better frame the gaps in current research and emphasize the contribution of our study. We have removed some less relevant background information (Page 5-6, lines 111-113)** **and placed greater emphasis on the shortcomings of existing predictive models for KOA progression, particularly in integrating biochemical biomarkers with radiological data. This revision ensures that the Introduction is more concise and directly aligned with the aims of our study.**

-Please provide more details on the statistical and machine learning methodologies, such as the rationale behind using LASSO logistic regression for feature selection and its effect on model interpretability

**Response: We thank the reviewer for this valuable comment. We agree that a more detailed explanation of the statistical and machine learning methodologies used in our study, particularly the rationale behind using LASSO logistic regression for feature selection, will enhance the clarity and transparency of our approach.**

**In response, we have expanded the Methods section to include the following points:**

**(1) Rationale for Using LASSO Logistic Regression: LASSO (Least Absolute Shrinkage and Selection Operator) logistic regression was chosen for feature selection because it is a regularized method that performs both variable selection and shrinkage. LASSO is particularly well-suited for high-dimensional datasets, like ours, where there are many predictors and potential multicollinearity. By penalizing the magnitude of coefficients, LASSO helps reduce overfitting and enhances the generalizability of the model by selecting only the most relevant features. (Page 12-13, lines 263-272)**

**(2) Impact on Model Interpretability: The selection of features via LASSO enhances model interpretability by identifying the most influential predictors, reducing the complexity of the model. By focusing on a smaller subset of relevant features, we make the model more interpretable and actionable for clinicians. The coefficients associated with the selected features can be directly interpreted as the strength and direction of their relationship with KOA progression, providing insights into the key factors driving disease progression.**

-Please, explain why a 1:1 split between the development and test cohorts was used, and discuss how this split ensures robust external validation. How does this approach to cohort splitting compare to alternative validation strategies, such as k-fold cross-validation, in terms of generalizability?

**Response: We appreciate the reviewer’s comment and agree that it is important to explain why a 1:1 split between the development and test cohorts was used, and discuss how this split ensures robust external validation. The decision to use a 1:1 split between the development and test cohorts was made to ensure robust external validation while maintaining sufficient statistical power in both cohorts. This approach allows the model to be trained on one subset and validated on an entirely independent subset, minimizing the risk of overfitting and offering an unbiased evaluation of predictive performance. Additionally, the 1:1 split ensures balanced representation of key demographic and clinical variables across both cohorts, thereby strengthening the external validity of our findings.**

**In our study, baseline, 1-year follow-up, and 2-year follow-up knee MRI images were included. At each time point, the sample sizes for the "Only JSN progression" and "Only pain progression" groups were approximately 100 cases each. Using a traditional 8:2 or 7:3 training-validation split would have resulted in only about 20 validation samples per group at each time point, which would be insufficient to capture meaningful progression patterns. The 1:1 split, therefore, maximized subgroup representation across all time points, improving the reliability and generalizability of our model's predictions.**

**When compared to k-fold cross-validation, a 1:1 split offers distinct advantages and trade-offs in our study. K-fold cross-validation is effective for internal validation and optimal data utilization by rotating training and validation sets across folds. However, it can sometimes yield overly optimistic performance estimates, especially in high-dimensional datasets. In contrast, the 1:1 split provides a transparent and realistic assessment of generalizability on unseen data. Nonetheless, we still employed k-fold cross-validation during the hyperparameter optimization stage to refine the model's performance metrics.**

**The reasons of why a 1:1 split between the development and test cohorts was used, and discuss how this split ensures robust external validation were added in Methods section** **(Page 11, line 235-237).**

-While the results are robust and well-documented, the authors should clarify : What defines a "successful" prediction? (e.g., accuracy threshold or clinical utility).

**Response: We thank the reviewer for this important comment. We agree that clarifying what defines a "successful" prediction in the context of our study is essential for better understanding the implications of the results.**

**In response, we have clarified the definition of a "successful" prediction in the Methods section (Page 15, lines 315-325). For this study, a successful prediction is defined as one that accurately identifies the correct outcome (e.g., KOA progression or non-progression) based on a combination of predictive accuracy and clinical relevance. Specifically:**

**(1) Accuracy Threshold: A prediction is considered successful if the model achieves an AUC of greater than 0.70, indicating an acceptable level of discriminative ability. This threshold is commonly used in the literature to denote a model that is sufficiently reliable for clinical decision-making (PMID: 35077357).**

**(2) Clinical Utility: In addition to statistical accuracy, we define clinical success by the model’s ability to assist in identifying high-risk patients who may develop into JSN progression or/and pain progression in next two years. The model's utility is further supported by its ability to integrate multiple biomarkers and clinical variables, offering a more holistic and actionable risk assessment.**

- In the Discussion Section please include a more explicit comparison with recent literature, highlighting where this study excels or falls short.

**Response: We thank the reviewer for this valuable suggestion. In response, we have expanded the Discussion section to include a clearer comparison with recent studies that have integrated MRI radiomics, biochemical biomarkers, and clinical risk factors for predicting KOA progression. Specifically:**

**(1) We have compared our model (LBTRBC-M) with recent studies such as Saarakkala et al. (2022), Lespessailles et al. (2024), and Hu et al. (2023), highlighting the strengths of our approach, including higher AUC values (0.869–0.920) and improved integration of multimodal data sources. (Page 23, lines 501-507)**

**(2) Our study excels in combining load-bearing tissue MRI radiomics with biochemical biomarkers and clinical variables in a longitudinal dataset, offering a more holistic assessment compared to studies focusing on isolated modalities. (Page 23-24, lines 507-512)**

**(3) We have acknowledged shared limitations with previous studies, including the lack of stratified cross-validation (Page 27, lines 583-592) and the need for further validation in external cohorts to ensure broader generalizability** **(Page 29, lines 621-624).**

**(4) Additionally, we recognize that while our approach demonstrates strong predictive accuracy, further improvements may be achieved by exploring advanced non-linear modeling techniques such as Generalized Additive Models (GAMs) or Spline Regression. (Page 29, lines 636-637)**

- While the discussion mentions the potential utility of the model for resident physicians, more emphasis is needed on real-world implementation barriers (e.g., cost, time, and training).

**Response: We appreciate the reviewer’s comment. Real-world implementation of the LBTRBC-M model faces key challenges, including costs associated with data acquisition and software integration, time constraints for data processing, and the need for clinician training to ensure proper interpretation and application of results. Additionally, addressing regulatory and ethical considerations, such as data privacy and transparency, is essential for widespread adoption. These barriers, along with potential solutions, have been briefly discussed in the revised Discussion section to highlight practical considerations for clinical integration. (Page 26-27, lines 574-582)**

- In some sections the English language needs to be polished to improve fluency and clarity.

**Response: We appreciate the reviewer’s comment. We have improved the fluency and clarity of English language in Introduction (Page 5, lines 93) and Discussion section (Page 24, lines 513-514).**

General editorial requests:

(Note: not all will apply to your paper, but please check each item carefully)

* We ask every co-author listed on the manuscript to fill in a contributing author statement, making sure to declare all competing interests. If any of the co-authors have not filled in the statement, we will remind them to do so when the paper is revised. If all statements are not completed in a timely fashion this could hold up the re-review process. If new competing interests are declared later in the revision process, this may also hold up the submission. Should there be a problem getting one of your co-authors to fill in a statement we will be in contact. Please do not add or remove authors without first discussing this with the handling editor. You can see our competing interests policy here: http://journals.plos.org/plosmedicine/s/competing-interests.

**Response: done.**

* Please upload any figures associated with your paper as individual TIF or EPS files with 300dpi resolution at resubmission; please read our figure guidelines for more information on our requirements: http://journals.plos.org/plosmedicine/s/figures. While revising your submission, please upload your figure files to the PACE digital diagnostic tool, https://pacev2.apexcovantage.com/. PACE helps ensure that figures meet PLOS requirements. To use PACE, you must first register as a user. Then, login and navigate to the UPLOAD tab, where you will find detailed instructions on how to use the tool. If you encounter any issues or have any questions when using PACE, please email us at PLOSMedicine@plos.org.

**Response: done.**

* Please ensure that the paper adheres to the PLOS Data Availability Policy (see http://journals.plos.org/plosmedicine/s/data-availability), which requires that all data underlying the study's findings be provided in a repository or as Supporting Information. For data residing with a third party, authors are required to provide instructions with contact information (web or email address) for obtaining the data. Please note that a study author cannot be the contact person for the data. PLOS journals do not allow statements supported by "data not shown" or "unpublished results." For such statements, authors must provide supporting data or cite public sources that include it.

**Response: done.**

* We expect all researchers with submissions to PLOS in which author-generated code underpins the findings in the manuscript to make all author-generated code available without restrictions upon publication of the work. In cases where code is central to the manuscript, we may require the code to be made available as a condition of publication. Authors are responsible for ensuring that the code is reusable and well documented. Please make any custom code available, either as part of your data deposition or as a supplementary file. Please add a sentence to your data availability statement regarding any code used in the study, e.g. "The code used in the analysis is available from Github [URL] and archived in Zenodo [DOI link]" Please review our guidelines at https://journals.plos.org/plosmedicine/s/materials-software-and-code-sharing and ensure that your code is shared in a way that follows best practice and facilitates reproducibility and reuse. Because Github depositions can

be readily changed or deleted, we encourage you to make a permanent DOI'd copy (e.g. in Zenodo) and provide the URL.

**Response: done.**

* FINANCIAL DISCLOSURES: The funding statement should include: specific grant numbers, initials of authors who received each award, URLs to sponsors’ websites. Also, please state whether any sponsors or funders (other than the named authors) played any role in study design, data collection and analysis, the decision to publish, or preparation of the manuscript. If they had no role in the research, include this sentence: “The funders had no role in study design, data collection and analysis, decision to publish, or preparation of the manuscript.”

**Response: done.**

*	DATA AVAILABILITY: The Data Availability Statement (DAS) requires revision. For each data source used in your study:

a) If the data are freely or publicly available, note this and state the location of the data: within the paper, in Supporting Information files, or in a public repository (include the DOI or accession number).

b) If the data are owned by a third party but freely available upon request, please note this and state the owner of the data set and contact information for data requests (web or email address). Note that a study author cannot be the contact person for the data.

c) If the data are not freely available, please describe briefly the ethical, legal, or contractual restriction that prevents you from sharing it. Please also include an appropriate contact (web or email address) for inquiries (again, this cannot be a study author).

**Response: The data that support the findings of this study are available from the corresponding authors with a signed data access agreement. De­identified patient­level clinical data, outcome data and MRI data are available at https://nda.nih.gov/oai/. (Page 31, lines 668-670)**

* ETHICS STATEMENT: Please provide the name(s) of the institutional review board(s) that provided ethical approval. Please specify whether informed consent was written or oral.

**Response:** **All de­identified patient­ level clinical data, outcome data, and MRI data in our study were obtained from the OAI, an ongoing, multicenter, prospective cohort study designed to identify biomarkers for KOA. The Health Insurance Portability and Accountability Act (HIPAA) compliant protocol of the OAI study had received institutional review board (IRB) approval. (Page 3, lines 63-68)**

FORMATTING - GENERAL

* Abstract: Please structure your abstract using the PLOS Medicine headings (Background, Methods and Findings, Conclusions). Please combine the Methods and Findings sections into one section.

**Response: done.**

* At this stage, we ask that you include a short, non-technical Author Summary of your research to make findings accessible to a wide audience that includes both scientists and non-scientists. The Author Summary should immediately follow the Abstract in your revised manuscript. This text is subject to editorial change and should be distinct from the scientific abstract. Ideally each sub-heading should contain 2-3 single sentence, concise bullet points containing the most salient points from your study. In the final bullet point of 'What Do These Findings Mean?', please include the main limitations of the study in non-technical language. Please see our author guidelines for more information: https://journals.plos.org/plosmedicine/s/revising-your-manuscript#loc-author-summary.

**Response:**

**Author Summary**

**Why Was This Study Done?**

**(1) Knee osteoarthritis (KOA) is a common and debilitating joint disease with limited treatment options.**

**(2) Early prediction of KOA progression can help identify high-risk patients and guide personalized treatment strategies.**

**(3) This study aimed to develop an advanced predictive model combining MRI radiomics, biochemical biomarkers, and clinical risk factors to accurately predict KOA progression.**

**What Did the Researchers Do and Find?**

**(1) We analyzed data from 594 participants, including MRI scans, biochemical biomarker levels, and clinical assessments over two years.**

**(2) Using machine learning algorithms, we developed the LBTRBC-M predictive model that integrates MRI radiomics, biochemical biomarkers, and clinical variables.**

**(3) The model achieved high predictive accuracy (AUC 0.869–0.920) and significantly improved the ability of resident physicians to predict KOA progression.**

**What Do These Findings Mean?**

**(1) The LBTRBC-M model offers a promising tool for identifying patients at high risk of KOA progression, potentially enabling early intervention and personalized treatment.**

**(2) Integration of radiomic and biomarker data enhances prediction accuracy beyond traditional clinical assessments.**

**(3) However, further validation in diverse populations and addressing implementation barriers in clinical practice are necessary before widespread adoption.**

* Please express the main results with 95% CIs as well as p values. When reporting p values please report as p<0.001 and where higher as the exact p value p=0.002, for example. Throughout, suggest reporting statistical information as follows to improve clarity for the reader "22% (95% CI [13%,28%]; p</=)". Please be sure to define all numerical values at first use.

**Response: done.**

* Please include page numbers and line numbers in the manuscript file. Use continuous line numbers (do not restart the numbering on each page).

**Response: done.**

* Please cite the reference numbers in square brackets. Citations should precede punctuation.

**Response: done.**

FIGURES AND TABLES

* Please provide titles and legends for all figures and tables (including those in Supporting Information files).

**Response: done.**

* Please define all abbreviations used in each figure/table (including those in Supporting Information files).

**Response: done.**

* Please consider avoiding the use of red and green in order to make your figure more accessible to those with color blindness.

**Response: OK.**

SUPPLEMENTARY MATERIAL

* Please note that supplementary material will be posted as supplied by the authors. Therefore, please amend it according to the relevant comments outlined here.

**Response: OK.**

* Please cite your Supporting Information as outlined here: https://journals.plos.org/plosmedicine/s/supporting-information

**Response: done.**

REFERENCES

* PLOS uses the numbered citation (citation-sequence) method and first six authors, et al.

**Response: done.**

* Please ensure that journal name abbreviations match those found in the National Center for Biotechnology Information (NCBI) databases (http://www.ncbi.nlm.nih.gov/nlmcatalog/journals), and are appropriately formatted and capitalised.

**Response: done.**

* Where website addresses are cited, please include the complete URL and specify the date of access (e.g. [accessed: 12/06/2024]).

**Response: done.**

* Please also see https://journals.plos.org/plosmedicine/s/submission-guidelines#loc-references for further details on reference formatting.

**Response: done.**

STUDY TYPE-SPECIFIC REQUESTS

The following list is derived from Geoffrey P Garnett, Simon Cousens, Timothy B Hallett, Richard Steketee, Neff Walker. Mathematical models in the evaluation of health programmes. (2011) Lancet DOI:10.1016/S0140-6736(10)61505-X:

* If pertinent, please provide a diagram that shows the model structure, including how the natural history of the disease is represented, the process and determinants of disease acquisition, and how the putative intervention could affect the system.

**Response: done.**

* Please provide a complete list of model parameters, including clear and precise descriptions of the meaning of each parameter, together with the values or ranges for each, with justification or the primary source cited and important caveats about the use of these values noted.

**Response: done (Table S15).**

* Please provide a clear statement about how the model was fitted to the data, including goodness-of-fit measure, the numerical algorithm used, which parameter varied, constraints imposed on parameter values, and starting conditions.

**Response: Thank you for the helpful comment. In this study, the model was fitted using XGBOOST, an efficient gradient tree boosting algorithm. We employed cross-validation (10-fold) to evaluate and optimize the model's performance, ensuring robustness in the predictions. The goodness-of-fit was assessed using performance metrics such as AUC, accuracy, Logloss, and root average squared error (RASE). These metrics helped gauge the model’s overall predictive ability. We optimized the model by tuning key hyperparameters, including max_depth, learning_rate, subsample, and λ (lambda), using grid search with cross-validation. During optimization, we set practical constraints based on prior literature, such as limiting max_depth between 3 and 10 and learning_rate between 0.1 and 0.3. The algorithm’s parameters were initialized with default values, which were subsequently fine-tuned.** **(Page 14, lines 291-297)**

* For uncertainty analyses, please state the sources of uncertainties quantified and not quantified [can include parameter, data, and model structure].

**Response: Thank you for the valuable suggestion. In our study, we considered several sources of uncertainty (Page 28, lines 607-617), which include:**

**(1) Parameter Uncertainty: This relates to the potential variability in the model’s hyperparameters, such as max_depth, learning_rate, subsample, and λ (lambda). These parameters were optimized using grid search with cross-validation, but their optimal values are subject to variation, which can affect model performance.**

**(2) Data Uncertainty: The data used for training and testing the model may contain inherent noise and variability, such as measurement errors in MRI scans or biomarkers. Additionally, the imbalanced distribution of certain classes (e.g., progression vs. non-progression) could introduce uncertainty in model predictions, especially when the model encounters minority class cases.**

**(3) Model Structure Uncertainty: The XGBOOST algorithm, while robust, makes certain assumptions about data structure and relationships between variables. For instance, the decision tree-based nature of XGBoost assumes non-linear relationships between predictors, but it may not fully capture all complex interactions, particularly for certain biomarkers or imaging features. Further, the absence of stratified cross-validation could have influenced how well the model generalizes across different subgroups.**

* Please provide sensitivity analyses to identify which parameter values are most important in the model. Uncertainty estimates seek to derive a range of credible results on the basis of an exploration of the range of reasonable parameter values. The choice of method should be presented and justified.

**Response: Thank you for the suggestion.** **To evaluate model robustness, we performed a sensitivity analysis by varying key hyperparameters (Table S16) such as max_depth, learning_rate, subsample, and λ (lambda). We used Delong test to assess the effect of each parameter on model performance. This analysis will identify the most influential parameters and quantify uncertainty, improving the model's reliability and understanding its behavior in predicting KOA progression. (Page 21-22, lines 463-468)**

* Please discuss the scientific rationale for the choice of model structure and identify points where this choice could influence conclusions drawn. Please also describe the strength of the scientific basis underlying the key model assumptions.

**Response:** **We chose XGBOOST for its ability to handle both linear and non-linear relationships, making it ideal for complex data with MRI radiomics, biomarkers, and clinical factors. Its decision tree-based approach captures interactions between variables and prevents overfitting through regularization. However, its "black-box" nature limits interpretability and may emphasize certain variables over others. While scientifically justified, these assumptions may influence conclusions. Future studies could explore more transparent models, like Generalized Additive Models (GAMs), to improve clarity and accuracy. (Page 27-28, line 593-599)**

* For studies that develop a prediction model or evaluate its performance, please ensure that the study is reported according to the TRIPOD statement (https://www.equator-network.org/reporting-guidelines/tripod-statement) and include the completed checklist as Supporting Information. Please add the following statement, or similar, to the Methods: "This study is reported as per the Transparent Reporting of a Multivariable Prediction Model for Individual Prognosis Or Diagnosis (TRIPOD) statement (S1 Checklist)." For studies using machine learning, please use the TRIPOD-AI checklist. When completing the checklist, please use section and paragraph numbers, rather than page numbers.

**Response: done.**

During the revision process, Dr. Liu Hao made substantial contributions to the study, and all authors unanimously agree to include Dr. Liu Hao as a co-first author.

We greatly appreciate the time and effort invested by the reviewers and the editor in evaluating our manuscript. We believe that the revisions have strengthened the clarity, rigor, and overall quality of our work. We look forward to your positive consideration for publication.

Yours sincerely,

Professor Changhai Ding, Ph.D.

(On behalf of all authors)
