# Supplementary material for: Predicting knee osteoarthritis progression using neural network with longitudinal MRI radiomics, and biochemical biomarkers: A modeling study
Source: PLoS Med. 2025 Aug 21;22(8):e1004665. doi: 10.1371/journal.pmed.1004665 (PMC12370028; doi:10.1371/journal.pmed.1004665)
Supplement: Attachment — Submitted filename: PMEDICINE-D-24-02732R2-Response to Review.docx [file pmed.1004665.s040.docx]

Dear editors and reviewers,

Thank you for your detailed feedback on our manuscript entitled "Predicting KOA Progression: Integrating Neural Network, Longitudinal MRI Radiomics, and Biochemical Biomarkers" (PMEDICINE-D-24-02732R1). We appreciate the opportunity to revise our submission in accordance with the editorial requirements. Please find our responses below:

Reviewer #1: Thank you for thoroughly addressing all my comments and providing detailed clarifications in your revised manuscript. I appreciate the effort you have put into enhancing the rigor and transparency of your analyses. The manuscript now reflects a robust and valuable contribution to subject, I am happy to recommend this work for publication and look forward to seeing its positive impact in the field.

**Response: We sincerely appreciate your thoughtful and encouraging comments. Thank you for recognizing the revisions and the effort invested in addressing your suggestions. We are grateful for your recommendation for publication and are pleased that you found our work to be a valuable contribution to the field.**

Reviewer #2: R1- We thank the authors for clarifying their rationale for performing 25,000 repetitions of cross-validation. While ensuring stability in performance estimates is important, the justification for such an unusually high number remains unclear, especially since they acknowledge that predictive accuracy plateaued before reaching this level. Beyond that point, the additional computational burden becomes difficult to justify. A quantitative analysis demonstrating how variance decreases across iterations would strengthen their argument. Their plan to explore fewer iterations in future studies is good, but a more systematic justification for their current approach is essential to enhance the study's methodological transparency and rigor. So we request the authors to include this justification in the current manuscript.

**Response:** **We appreciate the reviewer’s thoughtful feedback on our use of 25,000 iterations for cross-validation. In our revised manuscript, we have reduced the number of iterations to 100 based on the observed plateau in model performance and computational efficiency.**

**To further address the reviewer’s concern and provide a more systematic justification, we conducted a comparative analysis of model performance across different iteration settings: 100, 300, 1000, and 25,000. The results demonstrated that AUC values showed only marginal differences across these settings. For example, for JSN and pain progression, the AUC was 0.880 (95% CI: 0.853–0.903) at 100 iterations and 0.868 (95% CI: 0.841–0.892) at 25,000 iterations (ΔAUC = 0.012, 95% CI: 0.002–0.021, p = 0.015). Similarly, differences in AUC for JSN progression, pain progression, and non-progression were statistically insignificant or modest (Table S18).**

**Moreover, the time consumption increased exponentially with more iterations—from 6 seconds (100 iterations) to over 4 hours (25,000 iterations), with only minimal improvements in performance metrics. These findings support the conclusion that 100 iterations are sufficient for stable and reliable model evaluation, aligning with the need to balance methodological rigor with computational feasibility. We have included this data and its analysis in the revised manuscript to enhance transparency and justify our approach.**

**We have now clarified this rationale in the revised manuscript (Page 12, lines 259-263, Model development and test section).**

R2- Once again, we thank the authors for recognizing the significance of clarifying the implications of their high number of iterations, especially with relation to computing feasibility. Although they use robustness to support their decision, further explanation is required to properly address methodological trade-offs and feasibility.

The computational feasibility is still unclear. Cross-validation with 25,000 iterations can be computationally challenging, particularly when dealing with high-dimensional data. To show that their method is practical and scalable, it would be beneficial if the authors provide information on hardware specs, runtime, and resource needs.

The authors also do not provide quantitative proof to support the need for 25,000 iterations, even though they claim that less iterations (such as 100-300) resulted in higher variability in performance indicators. A statistical analysis (such as a graphic showing the relationship between variability) would show clearly where performance metrics stabilize and whether this high number of iterations is justified.

**Response:** **We appreciate the reviewer’s comment regarding computational feasibility.** **The specifications of our hardware specs were Intel® Core™ i5-1135G7 CPU @ 2.40GHz, 16 GB RAM, and a 512 GB SSD (Page 12, lines 263-265, Model development and test section). Despite the relatively modest hardware, the model training at 100 iterations completed in approximately 6 seconds, and the most computationally intensive run with 25,000 iterations was completed in 4 hours and 12 minutes (15,152 seconds).**

**We have also added a summary table (Table S18) showing AUC stability across different iteration levels (100, 300, 1000, and 25,000) with corresponding time costs and statistical differences. As demonstrated, model performance stabilized after 100 iterations with minimal gains thereafter (e.g., ΔAUC = 0.012 for JSN and pain progression, p = 0.015), supporting our decision to use 100 iterations in the final analysis as a balanced trade-off between performance reliability and computational efficiency (Page 12, lines 259-263, Model development and test section).**

R3- We appreciate the authors' detailed clarification of the LASSO feature selection process, which greatly enhances the clarity of their methodology. As an optional improvement, we suggest including a comparison between the number of features initially considered and those retained after LASSO selection.

**Response: Thank you for your thoughtful suggestion. As requested,** **we have included a comparison of the number of features before and after LASSO selection. Initially, 2,947 features were considered in the model: 2,922 radiomic features, 18 biochemical biomarkers, and 7 clinical variables. After applying LASSO, 255 non-zero features were retained in the final model, including 236 radiomic features, 13 biochemical biomarkers, and 6 clinical variables. This comparison has been added to the Methods section to further clarify the dimensionality reduction process (Page 12-13, line 265-270).**

R4- Authors response acknowledges the limitation regarding the lack of temporal analysis of feature dynamics over time. Addressing it as a limitation in their discussion section is sufficient.

**Response: Thank you for recognizing our acknowledgment of this limitation. As noted in the Discussion section (Page 30, lines 652–656, limitation 12th), we have highlighted the lack of temporal analysis of feature dynamics over time as a limitation. We appreciate your understanding that this addition adequately addresses the concern.**

R5- We appreciate the authors' detailed response concerning the clinical relevance of their predictive model.

**Response:** **Thank you for your kind feedback. We are pleased that our explanation clarified the clinical relevance of our predictive model. We hope the integration of radiomic, biochemical, and clinical data will support future clinical applications in KOA management.**

R6- Although the authors have cited 3 recent papers from 3 different research teams, the conclusions drawn about their results are not entirely fair and balanced. Specifically, they state, 'Compared to these studies, our model (LBTRBC-M) achieved higher AUC values (0.869-0.920) by integrating load-bearing tissue MRI radiomics, biochemical biomarkers, and clinical variables. This suggests a more comprehensive risk assessment and superior predictive accuracy.' However, the results from their study and those from the other cited studies cannot be directly compared, as the datasets and methodologies used in those studies differ. Comparing AUC values is only relevant in the context of 'challenge' studies, where different teams use the same dataset and aim for the same goal, as seen in the KOA progression prediction challenge (KNOAP2020, Ref.1). Therefore, the sentence from Line 526 to Line 529 should be deleted.

Ref.1: Hirvasniemi et al., The KNee OsteoArthritis Prediction (KNOAP2020) challenge: An image analysis challenge to predict incident symptomatic radiographic knee osteoarthritis from MRI and X-ray images, Osteoarthritis Cartilage (2023), 31(1):115-125. doi:10.1016/j.joca.2022.10.001

**Response:** **Thank you for your constructive feedback. We agree that direct comparison of AUC values across studies with different datasets and methodologies can be misleading. In response to your comment, we have deleted the sentence from Line 526 to Line 529 in the revised manuscript, as suggested. We appreciate your guidance in helping us improve the scientific rigor and fairness of our comparisons.**

Furthermore, I have several additional concerns that the authors should address before I can give positive consideration for publication:

Major concerns:

1) Although authors indicate that both TUKEY'S honestly significant difference and Bonferroni correction were applied to control for type I error due to multiple comparisons, they should provide to the reader the nominal P value which is now considered as statistically significant in their work. Thus, the P value indicated line 375 in the statistical analysis section is probably wrong due to the numerous comparisons made in the present study.

**Response: Thank you for your insightful comment. We agree that clarification is needed regarding the adjusted significance threshold. In our revised manuscript, we have specified the corrected P value thresholds based on the multiple comparison methods used. For analyses where Tukey’s HSD was applied, the software automatically adjusted the critical value for pairwise comparisons. For Bonferroni correction, the adjusted significance threshold was calculated as 0.05 divided by the number of comparisons. We have now revised Line 375 in the statistical analysis section to reflect this correction and provided the specific adjusted *P* value thresholds where applicable to improve clarity and transparency (Page 17, line 364-368).**

2) While DeLong's test is widely recognized as a non-parametric method for comparing the AUCs of different models, it does not fully account for the shared variance between nested models, as in this study. This limitation can lead to biased or overly conservative results (Demler et al., 2012; Ref. 1). To address this issue, the authors should consider using alternative approaches, such as bootstrap resampling or permutation-based testing.

Ref.1: Demler et al., Misuse of DeLong test to compare AUCs for nested models. Statistics in medicine, 31.23 (2012): 2577-2587

**Response:** **Thank you for your insightful comment. We acknowledge the limitation of using DeLong’s test in the context of nested models. Although the FNIH dataset was originally designed as a nested case-control study, in our analysis, the original case-control pairing was disrupted during the random 1:1 split into the Total Development and Total Test cohort. This disruption reduces the dependency between the models and mitigates some concerns about shared variance. Nonetheless, we agree that more robust comparative methods could further enhance the reliability of our findings. The updated methodology has been described in the revised Model development and test section**

**Although the FNIH dataset was originally constructed as a nested case-control study with matched pairs, we disrupted the original matching during the cohort split. Specifically, participants were randomly assigned to the development and test cohorts, disregarding their original case-control pairings. This strategy allowed us to evaluate model performance in a setting more reflective of real-world clinical variability and supported the use of generalized learning approaches, rather than those relying on strict pairwise comparison (Page 12, line 245-251, Model development and test section).**

3) Line 563-566: Typically, the clinical relevance of results is discussed when a statistically significant difference is observed. However, if the effect size is limited, the clinical relevance may be considered poor. In this study, the authors take a different approach, suggesting that a non-statistically significant result may still hold clinical relevance. I find this reasoning inappropriate, as statistical significance is a fundamental criterion for establishing meaningful clinical interpretation. Moreover, if the authors wish to argue for clinical relevance despite the absence of statistical significance, they should provide additional supporting evidence through other clinical metrics or relevant justifications, like PPV and NPV.

**Response:** **Thank you for this insightful comment. We agree that statistical significance is an important standard in clinical research. In our revision, we now acknowledge that the observed 6.9% increase in AUC was not statistically significant and therefore must be interpreted cautiously** **(Page 27, lines 585-589).**

4) Lines 327-329: The authors chose a 25% threshold for displaying results in red font based on the output of the LBTRBC-M model. However, this threshold seems somewhat arbitrary without further justification. It would be beneficial for the authors to provide a rationale for selecting 25% as the cut-off point, as this decision could impact on the interpretation of the results.

**Response:** **Thank you for this insightful comment. We agree that the rationale for the 25% threshold needed further clarification. In our study, the outcome variable has four mutually exclusive classes. Therefore, if the model were making predictions by chance, the expected probability for each class would be 25%. Based on this, we used 25% as a baseline reference for random prediction. Probabilities exceeding this threshold were considered to indicate stronger model confidence and were therefore highlighted in red to help visually distinguish more confident predictions from lower-probability ones. We have now clarified this rationale in the revised manuscript (Page 15, lines 316-321).**

5) Line 599: The authors did not implement stratified cross-validation, which significantly affects the validity of their comparisons to other studies. Without stratification, the distribution of cases and controls across folds may be highly imbalanced, leading to misleading model performance metrics. In particular, folds with a much lower Case/Control ratio can artificially inflate the AUC, as the model may achieve high accuracy primarily by correctly predicting the majority class (controls) rather than effectively distinguishing progressors. This can create an overestimation of the model's discriminatory power and reduce the reliability of the results. To ensure a fair and meaningful comparison with other studies and to obtain robust performance estimates, the authors should implement stratified cross-validation before final acceptance. This will help maintain a consistent class distribution across folds, preventing potential bias in model evaluation. Acknowledging the lack of stratification in the cross validation process as the authors do in their revised manuscript is not sufficient for quality of this kind of study.

**Response:** **Thank you for raising this important concern regarding stratified cross-validation. We agree that imbalanced class distributions across folds could lead to biased model evaluation.**

**To ensure a balanced distribution of outcome types across datasets, we performed a stratified split of the total development and test cohort for the LBTRBC-M model, maintaining an approximate 2:1:1:2 ratio of JSN and pain progression, JSN progression, pain progression, and non-progression (Figure S16A-B). Prior to stratification, the AUCs (95% CI) in the total test cohort were: 0.880 (0.853-0.903) for JSN and pain progression, 0.913 (0.881-0.937) for JSN progression, 0.886 (0.856-0.910) for pain progression, and 0.909 (0.888-0.926) for non-progression. Following stratification, the corresponding AUCs (95% CI) were: 0.853 (0.826-0.877) for JSN and pain progression (ΔAUC (95% CI) = 0.027 [(-0.003)-0.058], p = 0.080), 0.860 (0.823-0.891) for JSN progression (ΔAUC = 0.053 (0.016-0.090), p = 0.005), 0.878 (0.843–0.906) for pain progression (ΔAUC = 0.008 [(-0.031)-0.047], p = 0.697), and 0.853 (0.824–0.877) for non-progression (ΔAUC = 0.056 (0.030-0.082), p < 0.001)(Figure S16C-D, Table S19). (Page 24, line 514-526, Result section)**

**To mitigate class imbalance, we applied a stratified cohort split in the LBTRBC-M model to ensure proportional representation of each KOA progression subtype. This improved evaluation fairness and revealed that stratification notably reduced AUC inflation in JSN progression and non-progression groups. These results underscore the necessity of stratified validation in multi-class modeling to enhance generalizability and avoid performance overestimation. (Page 29, line 5624-629, Discussion section)**

Minor concerns:

1) Throughout the paper, the authors should consistently specify that they are using "MRI radiomics". When 'radiomics' is mentioned without this clarification, readers may assume that X-ray radiomics was also studied, which is not the case.

**Response:** **Thank you for your helpful suggestion. We agree that clearly specifying the imaging modality is important for reader clarity. In the revised manuscript, we have carefully reviewed all instances of the term "radiomics" and consistently revised them to "MRI radiomics" throughout the text to avoid any ambiguity regarding the imaging source. We confirm that only MRI-based radiomic features were included in this study, and no X-ray radiomics were analyzed.**

2) The authors do not report the 95% confidence interval (CI) values for the AUC throughout the paper. Reporting the 95% CI is essential for assessing the precision and reliability of the AUC estimates. Without these intervals, it is difficult to evaluate the statistical significance and the robustness of the model's performance. I strongly recommend including the 95% CI for AUC to provide a more comprehensive interpretation of the results.

**Response: Thank you for your valuable suggestion. We agree that reporting the 95% confidence intervals (CI) for AUC values is important for evaluating the robustness and precision of model performance. In response to your comment, we have added the corresponding 95% CI values for all reported AUCs throughout the manuscript, including both the main text and supplementary materials (see revised lines). We believe this addition improves the clarity and statistical rigor of our results.**

3) Line 154: The period of KOA progression should be specified upon its first mention. For example, it should be stated as 'KOA progression over 4 years' or 'KOA progression within the subsequent 2 years.

**Response:** **Thank you for your thoughtful comment. We agree that specifying the period of KOA progression at its first mention improves clarity. In the revised manuscript, we have updated the relevant sentence on Page 7, line 138 to state “KOA progression** **within the subsequent 2 years” to accurately reflect the study's follow-up period.**

4) Line 171: It would be better to specify that the 194 cases refer to those related to JSN and pain cases.

**Response:** **Thank you for your insightful comment. We agree that clarification is needed. In the revised manuscript, we have specified that the 194 cases refer to knees with both JSN and pain progression. The sentence on Page 7, line 155-156 now reads: “The FNIH OA Biomarkers Consortium undertook a nested case-control study (194** **JSN and pain progression cases and 406 OA comparators)” to enhance clarity.**

5) Line 217: It would be necessary to specify which serum and/or urine biochemical markers were included or at least provide a reference. The authors mention the number of biochemical markers on line 322, but do not specify which markers were studied, and this information is not provided until after four pages.

**Response: Thank you for pointing this out. In response, we have added a clarification and appropriate reference earlier in the manuscript. Specifically, on Page 10, line 202-203, we now state:**

**“Seventeen types of serum and urine biochemical markers were included in the FNIH cohort study, as detailed in reference (12).”**

6) Lines 410, 508, 510 and 559: It is important to specify what type of progression this value refers.

**Response:** **Thank you for your valuable comment. We agree that the term "KOA progression" should be clarified to avoid ambiguity. To avoid ambiguity, we have revised the manuscript to replace the term "KOA progression" with "JSN and/or pain progression" at Lines 410, 508, 510, and 559. These clarifications have been made throughout the manuscript to ensure consistent and precise terminology for the reader.**

We greatly appreciate the time and effort invested by the reviewers and the editor in evaluating our manuscript. We believe that the revisions have strengthened the clarity, rigor, and overall quality of our work. We look forward to your positive consideration for publication.

Yours sincerely,

Professor Changhai Ding, Ph.D.

(On behalf of all authors)
