# Supplementary material for: Predicting knee osteoarthritis progression using neural network with longitudinal MRI radiomics, and biochemical biomarkers: A modeling study
Source: PLoS Med. 2025 Aug 21;22(8):e1004665. doi: 10.1371/journal.pmed.1004665 (PMC12370028; doi:10.1371/journal.pmed.1004665)
Supplement: Attachment — Submitted filename: respond to reviewer and editor.docx [file pmed.1004665.s041.docx]

Dear Dr. Tosun,

Thank you for your decision letter and for the opportunity to revise our manuscript titled “Predicting KOA Progression: Integrating Neural network, Longitudinal MRI Radiomics, and Biochemical Biomarkers” (Manuscript ID: PMEDICINE-D-24-02732R3). We are grateful to you and the reviewers for your continued guidance and thoughtful feedback throughout the review process.

Here is a detailed point-by-point response to the reviewer and editorial comments:

Comments from Reviewers:

Reviewer #2:

I would like to sincerely thank the author to have consistently addressed all my concerns or comment in their last revised manuscript.

I am glad to consider that with its revisions the paper is now recommendable for publication.

**Respond: We sincerely thank the reviewer for your thoughtful feedback and encouraging comments. We are grateful for your time and effort in reviewing our manuscript through multiple rounds. Your insights have significantly strengthened the quality and clarity of our work. We are pleased that the revised version meets your expectations and is now recommendable for publication.**

Requests from Editors:

GENERAL

* Please confirm that your title complies with to PLOS Medicine’s style. Your title must be nondeclarative and not a question. It should begin with main concept if possible. “Effect of” should be used only if causality can be inferred, i.e., for an RCT. Please place the study design (“A randomized controlled trial,” “A retrospective study,” “A modelling study,” etc.) in the subtitle (i.e., after a colon).

**Respond:** **Thank you for your guidance regarding title formatting. We have revised the manuscript title to ensure it complies with PLOS Medicine’s style. Specifically, we have made the title nondeclarative, placed the main concept at the beginning, and included the study design in the subtitle.**

**Revised Title:**

**“Predicting Knee Osteoarthritis Progression Using Neural Networks, Longitudinal MRI Radiomics, and Biochemical Biomarkers: A Modeling Study” (Page 1, line 2).**

**We hope this revised title aligns with the journal’s requirements. Please let us know if any additional changes are needed.**

* Statistical reporting: Please revise throughout the manuscript, including tables and figures.

- Please report statistical information as follows to improve clarity for the reader ““22% (95% CI [13,28]; p</=)””.

**Respond:** **Thank you for your guidance regarding statistical reporting. In the revised manuscript, we have updated all statistical information to follow the recommended format.**

- Please separate upper and lower bounds with commas instead of hyphens as the latter can be confused with reporting of negative values.

**Respond: Thank you for your guidance regarding statistical reporting. We have carefully revised the manuscript to ensure that all confidence interval ranges are now reported using commas between the lower and upper bounds, in accordance with the recommended format.**

- Please repeat statistical definitions (HR, CI etc.) for each set of parentheses.

**Respond: Thank you for your valuable guidance regarding statistical reporting. In the revised manuscript, we have carefully repeated all statistical definitions (e.g., hazard ratio [HR], confidence interval [CI]) for each set of parentheses to ensure clarity and consistency throughout the text, in accordance with the journal’s formatting standards.**

* Please ensure that all abbreviations are defined at first use throughout the text (including statistical abbreviations). Please also check figures and tables.

**Respond:** **Thank you for your helpful suggestion. In the revised manuscript, we have ensured that all abbreviations, including statistical abbreviations, are defined at their first occurrence in the main text, figures, and tables to improve clarity and consistency.**

* Please ensure that tables and figures, including those in supplementary files, are appropriately referenced in the main text.

**Respond:** **Thank you for your suggestion. In the revised manuscript, we have carefully reviewed the main text to ensure that all tables and figures, including those in the supplementary materials, are appropriately referenced at relevant points to enhance readability and coherence.**

* Please note that the funding statement should also include URLs to sponsors’ websites.

**Respond: Thank you for your suggestion. The OAI is a nationwide research study, sponsored by the National Institutes of Health (part of the Department of Health & Human Services), that will help us better understand how to prevent and treat knee osteoarthritis, one of the most common causes of disability in adults. URLs to sponsors’ websites: https://www.nih.gov/.**

* Data availability: Please clarify whether, once access to the repository is gained (via the link you provided), it will be clear to anyone interested in the data which dataset was used for this study.

**Respond: Thank you for your comment. We have clarified the Data Sharing section in the revised manuscript to indicate that, upon accessing the repository via the provided link, readers will be able to clearly identify the specific dataset used in this study. We have also included the dataset name and relevant accession details to facilitate transparency and reproducibility (Page 37, line 818-825).**

* Many abbreviations are used throughout the main text. Please check whether the number could potentially be reduced to improve readability. Please also carefully check whether abbreviations are defined at first use throughout the text (including statistical abbreviations). Please also check figures and tables.

**Respond:** **Thank you for your valuable feedback. In the revised manuscript, we have carefully reviewed the use of abbreviations throughout the main text, figures, and tables. We have reduced the number of abbreviations where possible to improve readability and have ensured that all remaining abbreviations-including statistical terms-are clearly defined at their first appearance in the text and captions.**

* Please consider whether removing some of the numerical results in main text would improve readability in cases where the numbers are easy to find in the relevant table/figure.

**Respond:** **Thank you for your thoughtful suggestion. In the revised manuscript, we have reviewed the presentation of numerical results in the main text and removed or streamlined those that are readily available in the corresponding tables or figures, in order to enhance readability and avoid redundancy.**

* The manuscript is still quite complex and, at times, difficult to follow, particularly considering that PLOS Medicine serves a broad medical audience. Please keep this in mind when revising the manuscript. Please streamline the manuscript and create easy-to-follow methods and results sections. These sections should enable readers who are unfamiliar with the topic to understand your research.

**Respond:** **Thank you for your constructive feedback. We fully acknowledge the importance of making the manuscript accessible to a broad medical audience, in line with the readership of *PLOS Medicine*. In the revised version, we have streamlined the manuscript by simplifying complex language, restructuring paragraphs for clarity, and refining the Methods and Results sections to ensure they are more concise and easier to follow. These revisions aim to improve overall readability and ensure that readers without specialized expertise can clearly understand the study’s design, findings, and implications.**

* Because Github depositions can be readily changed or deleted, we encourage you to make a permanent DOI'd copy in Zenodo and provide the URL.

**Respond: Thank you for your helpful recommendation. In response, we have archived the source code on Zenodo to ensure permanent access and reproducibility. The DOI for the archived version is https://doi.org/10.5281/zenodo.15680828, and this information has been added to the revised Data Availability statement (Page 37, line 818-825).**

ABSTRACT

* Please confirm that your abstract complies with our requirements, including providing all the information relevant to this study type https://journals.plos.org/plosmedicine/s/submission-guidelines#loc-abstract

**Respond:** **Thank you for your reminder. We have carefully reviewed the abstract and revised it to ensure full compliance with *PLOS Medicine*’s requirements for this study type, as outlined in the submission guidelines. The abstract now includes all relevant components, including background, methods, results, and conclusions, in accordance with the journal’s formatting standards.**

* Please ensure that all numbers presented in the abstract are present and identical to numbers presented in the main manuscript text.

**Respond:** **Thank you for your feedback. We have carefully cross-checked the abstract and the main manuscript to ensure that all numerical values presented are consistent and accurately aligned across both sections.**

* Please suggest changing the description of outcomes to the following: “Outcomes included 1) both Joint Space Narrowing (JSN) and pain progression, 2) only JSN progression, 3) only pain progression, and 4) non-progression (JSN or pain)”.

**Respond:** **Thank you for the suggestion. We have revised the description of outcomes as recommended to: “Outcomes included 1) both Joint Space Narrowing (JSN) and pain progression, 2) only JSN progression, 3) only pain progression, and 4) non-progression (JSN or pain).” This updated phrasing has been incorporated into the relevant sections of the manuscript (Page 5, line 107-109).**

* We don’t think it’s currently 100% clear what you mean with “with a ratio of 2:1:1:2”. Please revise for clarity. We suggest including the exact numbers in the parentheses of the development and the total test cohort.

**Respond: Thank you for your helpful feedback. To improve clarity, we have revised the description of the outcome groups to explicitly include the exact number of knee MRIs in both the development and total test cohorts. The previously stated ratio of 2:1:1:2 has been replaced with the corresponding sample sizes for each outcome category to avoid ambiguity. The description of the outcome groups changing into “Outcomes included 1) both Joint Space Narrowing (JSN) and pain progression (n=567), 2) only JSN progression (n=303), 3) only pain progression (n=295), and 4) non-progression (JSN or pain) (n=588), corresponding to an approximate ratio of 2:1:1:2.” (Page 5-6, line 107-115)**

* “A total of 1753 knee MRIs were included over a 2-year follow-up.”We suggest describing this more accurately, providing the numbers for each time point.

**Respond:** **Thank you for the suggestion. We have revised the sentence to more accurately describe the number of knee MRIs at each time point. The updated wording is as follows:**

**“****A total of 1,753 knee MRIs were included across the study period, comprising 594 at baseline, 575 at 1-year follow-up, and 584 at 2-year follow-up.” (Page 5, line 105-106)**

*We suggest adding the definitions of JSN progression and pain progression in the Abstract.

**Respond:** **Thank you for the suggestion. We have added the definitions of JSN progression and pain progression to the Abstract as follows:** **JSN progression was defined as a minimum joint space width (JSW) loss of ≥0.7 mm, and pain progression as a sustained (≥2 time points) increase of ≥9 points on the Western Ontario and McMaster Universities Osteoarthritis Index (WOMAC) pain subscale (0-100 scale). (Page 5-6, line 112-115)**

* Please include basic demographics of the participants, i.e. mean age, sex, ethnicity/race.

**Respond:** **Thank you for your comment. We have included the basic demographic characteristics of the study participants in the revised manuscript as follows: mean age 61.6 ± 8.9 years; female: 349 participants (58.8%); race/ethnicity: White or Caucasian 471 (79.3%), Black or African American 107 (18.0%), Asian or Other Non-white 16 (2.7%).(Page 5, line 102-105)**

* Please define MRI at first use.

**Respond:** **Thank you for your comment. We have defined MRI as “Magnetic Resonance Image”** **at its first use in the manuscript to ensure clarity for all readers. (Page 5, line 96-97)**

* Why do you only report the AUC of the test and not the development cohort?

**Respond: Thank you for your question. We focused on reporting the AUC of the test cohort to evaluate the model’s generalizability and real-world performance on unseen data, which is a standard practice in predictive modeling. While the development cohort was used for training and internal tuning, its AUC values may be overly optimistic due to potential overfitting. So, we only reported the AUC of the development cohort.**

* Please include the number of resident physicians.

**Respond: Thank you for your comment. We have revised the manuscript to include the number of resident physicians involved in the study. A total of seven resident physicians participated. (Page 6, line 124)**

* In the last sentence of the Abstract Methods and Findings section, please describe the main limitation(s) of the study’s methodology.

**Respond:** **Thank you for your suggestion. We have revised the last sentence of the Abstract’s Methods and Findings section to briefly describe the main methodological limitations of the study. The main limitations include the use of a non-routine MRI sequence, the lack of external validation in independent cohorts, and limited incorporation of all knee joint structures in radiomic feature extraction. (Page 6, line 124-126)**

* Abstract Conclusions:

- Please address the study implications without overreaching what can be concluded from the data; the phrase ““In this study, we observed ...”” may be useful.

**Respond:** **Thank you for your thoughtful feedback. We have revised the discussion of the study implications to ensure they are well-aligned with the data and do not overstate the findings. We have adopted more measured language such as “In this study, we observed…” to clearly distinguish between observed results and broader interpretations. (Page 6, line 127-130)**

- Please interpret the study based on the results presented in the abstract, emphasizing what is new without overstating your conclusions.

**Respond: Thank you for your valuable suggestion. We have revised the interpretation of the study in the Abstract to ensure it is directly based on the results presented, highlights the novel aspects of our findings, and avoids overstating the conclusions. The updated wording emphasizes the contribution of longitudinal MRI radiomic features while maintaining a balanced and evidence-based tone.**

**Rewritten Conclusion:**

**In this study, we observed that longitudinal MRI radiomic features of load-bearing knee joint tissues provide novel and potentially informative markers for predicting knee osteoarthritis progression. These findings may help guide future efforts toward early risk stratification and personalized management of KOA. (Page 6, line 127-130)**

- Please avoid vague statements such as ““these results have major implications for policy/clinical care””. Mention only specific implications substantiated by the results.

**Respond:** **Thank you for your insightful feedback. We avoid vague or broad statements regarding implications for policy or clinical care.**

**Rewritten Conclusion:**

**In this study, we observed that longitudinal MRI radiomic features of load-bearing knee joint tissues provide novel and potentially informative markers for predicting knee osteoarthritis progression. These findings may help guide future efforts toward early risk stratification and personalized management of KOA. (Page 6, line 127-130)**

- Please avoid assertions of primacy (““We report for the first time…””)

**Respond:** **Thank you for your comment. We avoid any assertions of primacy such as “We report for the first time...”.**

AUTHOR SUMMARY

* It seems that the Author Summary of the previous version was not incorporated into the main text. Please revise. The Author Summary should follow the Abstract.

**Respond:** **Thank you for your comment. We have revised the manuscript to ensure that the Author Summary is properly incorporated immediately after the Abstract, as required by the journal. The Author Summary has been formatted using bullet points and written in accessible language to clearly communicate the study’s purpose, methods, key findings, and implications to a broad medical audience.**

* In the author summary, please revise formatting and ensure you use bullet points.

**Respond:** **Thank you for your suggestion. We have revised the Author Summary to follow the correct formatting guidelines and have presented the content using bullet points, as requested, to enhance clarity and readability for a broad medical audience.**

METHODS AND RESULTS

* Please consider reducing the number of subheadings.

**Respond:** **Thank you for your suggestion. We have reviewed the manuscript structure and reduced the number of subheadings to improve readability and maintain a more streamlined presentation of the content.**

* Ethics: Please clarify whether the need for ethical approval was waived for your study and if so, why. Please provide the name(s) of the institutional review board(s) together with the approval numbers that provided ethical approval.

**Respond: Thank you for your comment. Our study used only publicly available, de-identified data from the Osteoarthritis Initiative (OAI), an ongoing, multicenter, prospective cohort study. As such, no additional ethical approval was required for our secondary analysis.** **The original OAI study protocol was approved by the institutional review boards of all participating centers, including the coordinating center at the University of California, San Francisco (IRB number: 10-00532). The study was conducted in accordance with the Health Insurance Portability and Accountability Act (HIPAA), and all participants provided informed consent. (Page 4, line 70-73)**

* “The KLG assessments for radiographs were performed by Dr. Piran Aliabadi, MD and Dr. Burt Sack, MD, under the direction of Dr. David Felson, MD from the Boston University Clinical Epidemiology Research and Training Unit for the baseline through 24-month visits.”-We think it would be useful to add descriptions of job level and experience.

**Respond:** **Thank you for the suggestion. We have revised the manuscript to include descriptions of the professional roles and experience of the individuals involved in the KLG assessments.** **Specifically, Dr. Piran Aliabadi and Dr. Burt Sack are board-certified radiologists with extensive experience in musculoskeletal imaging, and the assessments were conducted under the supervision of Dr. David Felson, a senior rheumatologist and epidemiologist with decades of expertise in osteoarthritis research at the Boston University Clinical Epidemiology Research and Training Unit. (Page 13, line 285-289)**

* “Seventeen types of serum and urine biochemical markers were included in the FNIH cohort study, as detailed in reference (12).”we suggest describing these in detail here instead of using a reference only.

**Respond: Thank you for your suggestion. We have revised the manuscript to include a full list and description of the 18 serum and urine biochemical markers analyzed in the study, rather than referring to a single citation. This provides readers with immediate access to the relevant biomarker information and enhances the clarity and completeness of the Methods section.**

**Eighteen serum biomarkers included Cartilage Oligomeric Matrix Protein Cartilage Oligomeric Matrix Protein (sCOMP), Hyaluronic Acid (sHA), Type IIA Procollagen Amino-terminal Propeptide (sPIIANP), Type I Collagen C-terminal Telopeptide (sCTX-I), Aggrecan Chondroitin Sulfate 846 Epitope (sCS846), Matrix Metalloproteinase-3 (sMMP-3), Cleavage neoepitope of type II collagen (sC2C), Type II Collagen Neoepitope (sC1,2C), C-Propeptide of type II collagen (sCPⅡ), N-terminal Telopeptide of type I collagen (sNTX-Ⅰ), and Nitrated triple helix of type II collagen (sColl2-1NO2). Urine biomarkers included C-terminal cross-linked telopeptide of type Ⅰ collagen (α-isomer)(uCTX-Ⅰα), C-terminal cross-linked telopeptide of type I collagen (β-isomer)(uCTX-Ⅰβ), N-terminal cross-linked telopeptide of type I collage (uNTX-Ⅰ), Cleavage neoepitope of type II collagen (uC2C), Type II collagen neoepitope (uC1,2C), Nitrated triple helix of type II collagen (uColl2-1NO2), and C-telopeptide fragment of type II collagen (uCTX-Ⅱ). These markers reflect processes such as cartilage degradation and synthesis, bone turnover, and joint inflammation. (Page 13, line 292-304)**

* l.320: Please clarify whether the clinical variable was ‘race’, ‘ethnicity’ or ‘race/ethnicity’.

**Respond:** **Thank you for your comment. In our study, the clinical variable was recorded as “race” based on self-reported data in the Osteoarthritis Initiative (OAI) dataset. We have clarified this in the revised manuscript to avoid ambiguity.**

**In our study, the racial distribution was 79.3% White or Caucasian, 18.0% Black or African American, and 2.7% Asian or other non-White. (Page 5, line 102-105)**

* “At the baseline visit, 178 (61%) females were included in the development cohort 1, while 171 (57%) females were included in the test cohort 1 (Table S2).” – The description you have provided seems misleading. Please revise. Suggestion: “At the baseline visit, 293 (178/293, 61% females) individuals were included in the development cohort 1, while 301 (171/301, 57% females) individuals were included in the test cohort 1 (Table S2).”

**Respond:** **Thank you for your helpful suggestion. We have revised the sentence for clarity as follows:**

**“At the baseline visit, 293 individuals (178/293, 61% females) were included in development cohort 1, while 301 individuals (171/301, 57% females) were included in test cohort 1 (Table S2).” (Page 22, line 488-490)**

* The terms gender and sex are not interchangeable (as discussed in https://www.who.int/health-topics/gender#tab=tab_1 ); please use the appropriate term.

**Respond:** **Thank you for your comment. We have reviewed the manuscript and revised the terminology to ensure appropriate and consistent use of the term “sex” when referring to biological differences, in accordance with WHO guidelines. (Page 22, line 496)**

* l.493: “This analysis will identify the most influential parameters and quantify uncertainty, improving the model's reliability and understanding its behavior in predicting KOA progression.” -The use of future tense seems wrong-please revise.

**Respond:** **Thank you for your observation. We have revised the sentence to reflect the correct tense. The updated version now reads:**

**“This analysis aimed to identify the most influential parameters and quantify uncertainty, thereby improving the model’s reliability and enhancing understanding of its behavior in predicting KOA progression.” (Page 27, line 606)**

* Please check that any use of statistical terms (such as trend or significant) are supported by the data, and if not please remove them.

**Respond:** **Thank you for your comment. We have carefully reviewed the manuscript to ensure that all uses of statistical terms such as “trend” and “significant” are supported by appropriate statistical analyses. Any instances not directly supported by the data have been revised or removed to maintain accuracy and clarity.**

* Figure 1: Please consider splitting the figure into two separate figures.

**Respond: Thank you for your suggestion. We have revised Figure 1 by splitting it into two separate figures to improve clarity and visual interpretation. The updated figures and legends are now presented as Figure 1 and Figure 2 in the revised manuscript.**

* Figure 3: “The colored dotted line of yellow, orange, purple, green, blue, teal, and red represented the predictive performance change of Liu, Zhao, Cao, J Li, Chen, X Wang, Dang, and M Zhang, respectively.” – is it necessary to be able to identify the performer? We suggest removing the names and simply stating that each color belongs to one of the test individuals.

**Respond:** **Thank you for your suggestion. We agree that naming individual test performers is not essential to interpreting the figure. We have revised the legend to remove personal identifiers and now refer to each colored dotted line as representing a distinct test individual. This change improves clarity and aligns with best practices for anonymized reporting. (Page 43, line 1017-1018)**

* Figure 3: For H and I, why is there no heading (total test cohort)?

**Respond:** **Thank you for pointing this out. We have revised Figure 3 to include the missing heading “Total Test Cohort” for panels H and I to ensure consistency and clarity across all subfigures.**

DISCUSSION

* Pleas remove all subheadings.

**Respond:** **Thank you for your suggestion. We have removed all subheadings from the manuscript to ensure consistency with the journal’s formatting requirements.**

* We don’t think that it’s necessary to cite the ORs of other studies in parentheses throughout the discussion.

**Respond:** **Thank you for your feedback. We have revised the discussion section to remove the in-text citation of ORs from other studies in parentheses. Where relevant, we now summarize comparative findings more narratively to maintain clarity and focus on the interpretation of our own results.**

“Our predictive model shows clinical potential in managing KOA progression by integrating MRI radiomic features, biomarkers, and clinical data.” – Based on the discussion and its limitations, do you think this statement is supported, or would you rather say that the model is a step toward developing a model that could be used in a clinic? We feel it would be better to tone down any statement about clinical utility.

**Respond:** **Thank you for your thoughtful comment. We agree that the original statement may overstate the clinical applicability of our findings. In the revised manuscript, we have toned down the language to more accurately reflect the study’s scope and limitations. The statement now reads:**

**“****Our predictive model represents a step toward developing tools that could support clinical decision-making for KOA progression, pending further validation in diverse populations and clinical settings.” (Page 34, line 747-749)**

------------------------------------------------------------

General Editorial Requests

1) We ask every co-author listed on the manuscript to fill in a contributing author statement. If any of the co-authors have not filled in the statement, we will remind them to do so when the paper is revised. If all statements are not completed in a timely fashion this could hold up the re-review process. Should there be a problem getting one of your co-authors to fill in a statement we will be in contact. YOU MUST NOT ADD OR REMOVE AUTHORS UNLESS YOU HAVE ALERTED THE EDITOR HANDLING THE MANUSCRIPT TO THE CHANGE AND THEY SPECIFICALLY HAVE AGREED TO IT.

**Respond:** **Thank you for the reminder. We confirm that all listed co-authors have been informed and are in the process of completing their contributing author statements. We understand that failure to complete these statements in a timely manner may delay the re-review process. Additionally, we acknowledge that no authors will be added or removed without prior approval from the handling editor.**

2) Please ensure that the paper adheres to the PLOS Data Availability Policy (see http://journals.plos.org/plosmedicine/s/data-availability), which requires that all data underlying the study's findings be provided in a repository or as Supporting Information. For data residing with a third party, authors are required to provide instructions with contact information for obtaining the data. PLOS journals do not allow statements supported by “data not shown” or “unpublished results.” For such statements, authors must provide supporting data or cite public sources that include it.

**Respond:** **Thank you for your guidance. All original data used in our study were obtained from the publicly available Osteoarthritis Initiative (OAI) database. The OAI is a third-party, publicly funded project managed by the U.S. National Institutes of Health. The data are accessible through the National Institute on Aging (NIA) data archive at https://nda.nih.gov/oai. Researchers may access the data by registering for an account and agreeing to the data use terms. We have updated the Data Availability Statement in the manuscript accordingly to ensure full compliance with the PLOS Data Availability Policy.**

3) Please review your reference list to ensure that it is complete and correct. If you have cited papers that have been retracted, please include the rationale for doing so in the manuscript text, or remove these references and replace them with relevant current references. Any changes to the reference list should be mentioned in the rebuttal letter that accompanies your revised manuscript.

**Respond:** **Thank you for your comment. We have carefully reviewed the reference list to ensure it is complete, accurate, and up to date. No retracted papers have been cited in the current version of the manuscript. Any necessary updates or corrections to references have been made, and these changes are documented in the rebuttal letter accompanying the revised submission.**

In compliance with data protection regulations, you may request that we remove your personal registration details at any time. (Use the following URL: https://www.editorialmanager.com/pmedicine/login.asp?a=r). Please contact the publication office if you have any questions.

**Respond:** **Thank you for the information. We acknowledge that we may request the removal of personal registration details at any time in accordance with data protection regulations. Should the need arise, we will use the provided URL or contact the publication office directly with any questions.**

We sincerely thank the editor and reviewers for their thorough and insightful comments, which have significantly improved the clarity, rigor, and overall quality of our manuscript. We have carefully addressed all suggestions and concerns, and we believe the revised version better reflects the strength and relevance of our findings. We respectfully submit the revised manuscript for your further consideration and hope it meets the requirements for publication in PLOS Medicine. Please do not hesitate to contact us if any further clarification or revision is needed.

Thank you for your time and consideration.

Sincerely,

Dr. Shengfa Li (on behalf of all co-authors)
